# The catalytic mechanism of vitamin K epoxide reduction in a cellular environment

**DOI:** 10.1074/jbc.RA120.015401

**Published:** 2020-12-10

**Authors:** Guomin Shen, Weidong Cui, Qing Cao, Meng Gao, Hongli Liu, Gaigai Su, Michael L. Gross, Weikai Li

**Affiliations:** 1 Henan International Joint Laboratory of Thrombosis and Hemostasis, School of Basic Medical Science, Henan University of Science and Technology, Luoyang, Henan, China; 2 Department of Biochemistry and Molecular Biophysics, Washington University School of Medicine, St Louis, Missouri, USA; 3 Department of Chemistry, Washington University in St Louis, St Louis, Missouri, USA

**Keywords:** vitamin K epoxide reductase, vitamin K epoxide, quantitative mass spectrometry, covalent intermediate, warfarin, thiol oxidoreductase, mixed inhibition, integral membrane enzyme, DTT, dithiothreitol, ER, endoplasmic reticulum, GGCX, vitamin K–dependent γ-glutamyl carboxylase, hVKOR, human VKOR, K, vitamin K quinone, KH_2_, vitamin K hydroquinone, KO, vitamin K epoxide, KOH, 3-hydroxyl vitamin K, LC-MS/MS, liquid chromatography combined with tandem mass spectrometry, MS, mass spectrometry, MW, molecular weight, NEM, N-ethylmaleimide, PBS, phosphate buffered saline, SDS-PAGE, sodium dodecyl sulfate-polyacrylamide gel electrophoresis, TCEP, tris-(2-carboxyethyl) phosphine, TM, transmembrane helix, VKDP, vitamin K–dependent protein, VKOR, vitamin K epoxide reductase, W, warfarin, WT, wildtype

## Abstract

Vitamin K epoxide reductases (VKORs) constitute a major family of integral membrane thiol oxidoreductases. In humans, VKOR sustains blood coagulation and bone mineralization through the vitamin K cycle. Previous chemical models assumed that the catalysis of human VKOR (hVKOR) starts from a fully reduced active site. This state, however, constitutes only a minor cellular fraction (5.6%). Thus, the mechanism whereby hVKOR catalysis is carried out in the cellular environment remains largely unknown. Here we use quantitative mass spectrometry (MS) and electrophoretic mobility analyses to show that KO likely forms a covalent complex with a cysteine mutant mimicking hVKOR in a partially oxidized state. Trapping of this potential reaction intermediate suggests that the partially oxidized state is catalytically active in cells. To investigate this activity, we analyze the correlation between the cellular activity and the cellular cysteine status of hVKOR. We find that the partially oxidized hVKOR has considerably lower activity than hVKOR with a fully reduced active site. Although there are more partially oxidized hVKOR than fully reduced hVKOR in cells, these two reactive states contribute about equally to the overall hVKOR activity, and hVKOR catalysis can initiate from either of these states. Overall, the combination of MS quantification and biochemical analyses reveals the catalytic mechanism of this integral membrane enzyme in a cellular environment. Furthermore, these results implicate how hVKOR is inhibited by warfarin, one of the most commonly prescribed drugs.

The vitamin K cycle sustains the functions of vitamin K–dependent proteins (VKDPs) in a variety of physiological and cellular processes, including blood coagulation, bone and soft tissue mineralization, signal transduction, and cell proliferation (1, 2). During this cycle, vitamin K undergoes redox conversions that are catalyzed by two enzymes, vitamin K epoxide reductase (VKOR) and γ-glutamyl carboxylase (GGCX). GGCX couples the epoxidation of vitamin K hydroquinone (KH_2_) to the γ-carboxylation of specific glutamic acid residues in VKDPs, a posttranslational modification required for their activity. To maintain the γ-carboxylase activity, VKOR reduces vitamin K epoxide (KO) to the quinone form (K) and then back to the hydroquinone, KH_2_, thereby completing the vitamin K cycle (1, 3).

The vitamin K cycle occurs in the endoplasmic reticulum (ER), where VKOR and GGCX are both embedded in the ER membrane. Their active site faces the ER lumen, where the posttranslational modification of VKDPs takes place. The active site of human VKOR (hVKOR) contains a pair of cysteines, Cys132 and Cys135; this CXXC motif forms the redox center that directly reacts with substrates, as in other thiol oxidoreductases (4, 5). Sequence alignment of VKOR homologs shows that Cys132/Cys135 and another cysteine pair, Cys43/Cys51, are absolutely conserved (6). The crystal structures of a VKOR homolog suggests that, for hVKOR, Cys43/Cys51 are located in a loop region in the ER lumen, and Cys132/Cys135 are at the luminal end of a transmembrane helix (7, 8). During the hVKOR catalysis, the substrate reduction is coupled to the oxidization of Cys132 and Cys135 that form a disulfide (Fig. 1*A*). To regain the catalytic activity, these cysteines can be reduced through a sequential electron-transfer process mediated by Cys43 and Cys51 (Fig. S1) (7, 8, 9). This process, however, has not been taken into consideration by studies of hVKOR catalysis done previously using chemical modeling (10) and quantum mechanics calculations (11, 12). In addition, these previous studies assumed that the KO reduction by hVKOR starts from reduced Cys132/Cys135 at the active site. These cysteines in hVKOR, however, are known to undergo a redox equilibrium between reduced and oxidized states owing to the relatively oxidized environment in the ER lumen (13).

**Figure 1:**
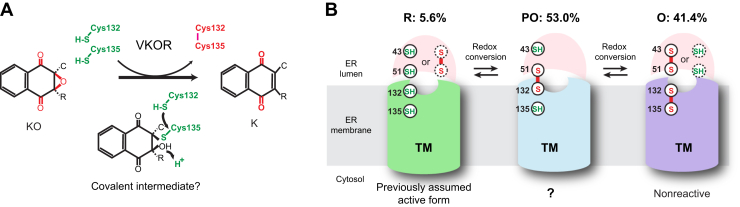
**Discrepancy between previous catalytic model of hVKOR and its cellular redox states.***A*, conventional model of hVKOR catalysis (11). Previous studies assume that KO reduction initiates with reduced Cys132/Cys135. The reduction results in the oxidation of Cys132/Cys135 to form a disulfide. The catalysis involves an intermediate state in which Cys135 is covalently linked to 3-hydroxyl vitamin K. Electron transfer from Cys132-SH resolves this covalent complex. Subsequently, the 3-hydroxyl group is protonated. The leaving of water completes the KO to K reduction. *B*, the cellular redox states of hVKOR. SH: reduced cysteine, S–S: disulfide bond. Quantitative MS shows that the state with reduced Cys132/Cys135 (R state) constitutes only 5.6% of the total cellular hVKOR. The remainder of hVKOR contains either a partially oxidized (PO state; Cys132 forms a disulfide with Cys51 and Cys135 reduced) or fully oxidized (O state; Cys132-Cys135 disulfide) active site. The TM region of hVKOR is shown as *green*, *blue*, and *purple* cyclinders for the R, PO, and O states, respectively. The luminal region is shown in *pink hemisphere*. Cysteines circled in dashed line indicate minor fraction of a certain state. The catalytic activity of partially oxidized hVKOR is unclear from previous studies.

Using live-cell mass spectrometry (MS)-based quantification (13, 14), we previously showed that only 5.6% of the cellular hVKOR carries reduced Cys132/Cys135 (hereafter named as the R state), which is the catalytically active form assumed in previous studies (Fig. 1*B*). On the other hand, 53.0% of cellular hVKOR is partially oxidized (PO state) with a Cys51–Cys132 disulfide and reduced Cys43/Cys135, and 41.4% of cellular hVKOR has an oxidized active site (O state) with the Cys132–Cys135 disulfide (Fig. 1*B*) (13). The O state is incapable of substrate reduction, whereas the R state can provide two electrons, Cys132-SH and Cys135-SH, to reduce KO to K. The PO state can also provide two electrons, Cys43-SH and Cys135-SH. If this PO state is catalytically active, it requires additional steps of electron transfer to reduce the substrates (Fig. S1). As mentioned above, the cellular abundances of hVKOR in R and PO states are much different, and their relative contributions to the cellular hVKOR activity are unclear. In addition, previous studies predicted that KO reduction involves a covalent reaction intermediate (Fig. 1*A*) (11), but the presence of this intermediate lacks experimental evidence.

Here we show that the covalent intermediate is potentially trapped with a Cys43Ala mutant that mimics the PO state but blocks the electron transfer. Comparison of the cellular activity and cellular cysteine status indicates that the PO and R states, despite of their different abundance, have similar contribution to the overall activity of hVKOR in cells. Because warfarin inhibits these two states with much different efficacy, warfarin may behave as a mixed inhibitor (15, 16).

## Results

### A covalent intermediate during KO reduction is potentially captured with a Cys43Ala mutant

Because hVKOR catalysis relies on electron transfer to proceed (Fig. S1), blocking the transfer may allow the capture of reaction intermediates during KO reduction. To investigate this possibility, we mutated each of the four cysteines involved in the electron-transfer process: Cys43Ala and Cys51Ala block the electron transfer to Cys132 at the active site, Cys132Ala blocks the electron transfer to Cys135, and Cys135Ala blocks the electron transfer to KO. We added KO to the cell lines stably expressing the cysteine mutants and wild-type hVKOR. After the KO incubation, the cells were treated with N-ethylmalemide (NEM) to block reduced cysteines, thereby preventing free cysteines to resolve reaction intermediates. Subsequently, the cell lysates were applied to a non-reducing SDS-PAGE to preserve cysteine links, and hVKOR species were subsequently detected by immunoblots.

**Figure 2:**
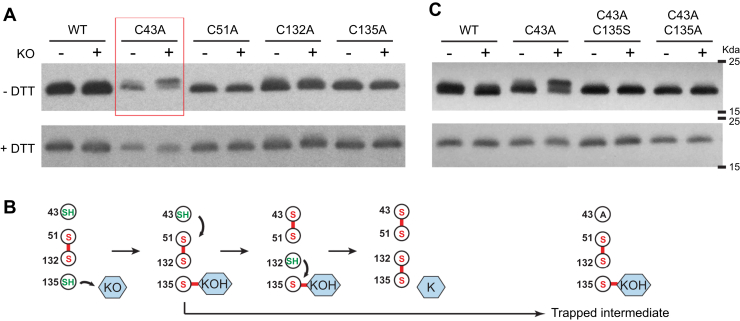
**Capture of a covalent reaction intermediate using a Cys43Ala mutant.***A*, *Top*, the anti-flag immunoblot under the nonreducing condition (-DTT) shows that KO (10 μM) treatment increases the apparent MW of the Cys43Ala mutant. *Bottom*, under the reducing condition (+DTT, 100 mM), KO treatment cannot induce this MW change of Cys43Ala. *B*, explanation of trapping of Cys43Ala by the covalent complex. *Left*, a proposed catalytic pathway with hVKOR in PO state. Cys135 attacks KO to form the covalent intermediate that Cys135 is linked to 3-hydroxyl vitamin K (KOH). Electron transfer from Cys43 generates a reduced Cys132 that subsequently resolves the covalent complex to generate the reaction product, K. *Right*, the Cys43Ala mutation blocks the electron-transfer process required for resolving the covalent complex. *C*, trapping the covalent complex requires the free thiol group of Cys135. The additional Cys135Ser or Cys135Ala mutation abolishes the KO-induced mobility change of Cys43Ala. The immunoblots are conducted in the same way as in *A*.

Interestingly, we find that the Cys43Ala mutant shows a slower electrophoretic mobility after the KO treatment under the nonreducing condition (Fig. 2*A*, upper panel). The slight increase in the apparent molecular weight of the Cys43Ala mutant is consistent with the attachment of KO (467 Da) to the hVKOR protein. To our surprise, such mobility change is not observed for the Cys132 mutant after the KO treatment; according to previous models, this mutant may trap the Cys135–KO intermediate by stopping the Cys132 attack (Fig. 1*A*). The wild-type hVKOR or other cysteine mutants also do not show the mobility change after the KO treatment. Moreover, Cys43Ala loses the mobility change after DTT reduction (Fig. 2*A*, lower panel), which can effectively resolve cysteine-linked reaction intermediates, but cannot change the irreversible NEM labeling. Taken together, these results suggest that the Cys43Ala mutant, after KO treatment, may form a cysteine-linked adduct. This intermediate is stable under the denaturation condition of SDS-PAGE, indicating that disrupting the substrate-binding interactions in the native protein does not affect the cysteine link. Instead, the cysteine link is formed on the protein polypeptide chain; a covalent bond can explain this preserved link after the protein denaturation.

Using MS quantification, we previously analyzed the cellular cysteine status of the Cys43Ala mutant in the absence of KO. We found that Cys51 and Cys132 are mainly oxidized and Cys135 is mainly reduced (13). Cys135 is the second cysteine in the CXXC motif and is expected to directly pass electrons to the substrate, as for other thiol oxidoreductases (17). Thus, the free thiol of Cys135 is capable of reacting with KO, consistent with the formation of the covalent adduct. Since the electrophoretic mobility of the Cys43Ala mutant suggests the trapping of this adduct, we propose that an electron-transfer process involving Cys43 is required to resolve this Cys135-linked reaction intermediate (Fig. 2*B*, left) and that Cys43Ala blocks this process (Fig. 2*B*, right).

To provide further evidence that Cys135 forms the covalent reaction intermediate, we analyzed the electrophoretic mobility of two double mutants, Cys43Ala/Cys135Ala and Cys43Ala/Cys135Ser (Fig. 2*C*). As expected, we find that these additional Cys135 mutations abolish the mobility change of Cys43Ala after the KO treatment, indicating that Cys135 is required to form the covalent link. In addition, although Cys135Ser may provide a hydrogen-bonding interaction to KO, no mobility change was observed for the Cys43Ala/Cys135Ser double mutant. Instead, forming the stable covalent intermediate requires the free thiol of Cys135 that acts as a reactive nucleophile.

The potential presence of this Cys135-linked covalent complex is further supported by our recently published crystal structures and NMR data (18). Crystal structure of hVKOR Cys43Ser mutant with KO shows that the distance between the sulfur atom of Cys135 and the C2 atom of the naphthoquinone ring is ∼2 Å, consistent with distance of a covalently bonded thioether adduct. For NMR analysis, we used KO carrying a ^13^C isotope label on its 2-methyl group, which allows the use of two-dimensional ^1^H-^13^ C HSQC spectrum to distinguish the thioether adduct (*i.e.*, mercapto 3-OH K) from KO and K, because the ^13^C 2-methyl group in these compounds adopts different bond angles relative to the naphthoquinone ring, whose ring current changes the chemical shift of the ^13^C 2-methyl group. After protease K digestion of the Cys43Ser mutant protein incubated with KO, we observed signals consistent with the thioether adduct, suggesting that a Cys135-linked covalent complex is retained.

### Formation of the potential covalent intermediate depends on the cellular cysteine status of hVKOR

**Figure 3:**
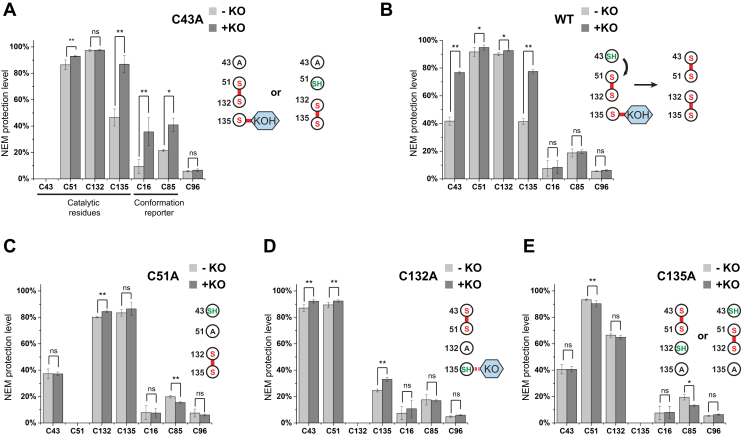
**Quantitative MS analysis of the cellular cysteine status suggests covalent complex formation and hVKOR conformational change.** The NEM protection level of cysteines is compared for wild-type and mutant hVKOR with and without KO (10 μM) treatment. *A*, for the Cys43Ala mutant, Cys135 can be protected from NEM modification when Cys135 forms a covalent complex after the KO treatment (*inset*). Alternatively, Cys135 may be protected owing to the formation of the Cys132–Cys135 disulfide. Cys16 and Cys85 become protected owing to the reduced structural flexibility of hVKOR with the covalently bound complex; similar protection was observed with warfarin binding (13). *B*, wild-type hVKOR (WT). Cys43 and Cys135 become protected after KO treatment owing to the formation of the Cys43–Cys51 and Cys132–Cys135 disulfide bonds (*inset*). With excess KO driving the reaction and electron transfer from Cys43, most of the covalently complex is resolved in the wild-type hVKOR. *C*–*E*, control experiments with other cysteine mutants. See text for explanations. Eror bars are standard deviations. Two-way Student's *t*-test (∗∗*p* < 0.01, ∗*p* < 0.05, ns, not significant) was performed for each cysteine from samples with and without KO treatment. The errors are calculated from a combination of three biological repeats and MS analyses of different peptides containing the same cysteine (six peptides for Cys16; three peptides for Cys43 and Cys51; and one peptide for Cys85, Cys96, Cys132, and Cys135). Detailed analysis of the MS data are presented in Tables S1 and S2, Fig. S2, and the Supplementary Excel File.

The live-cell quantitative MS method that we recently developed allows the monitoring of each cysteine in hVKOR for their accessibility and reactivity by NEM labeling, reporting the changes of protein conformation and cysteine status, respectively (13, 14, 19). To investigate these changes during the hVKOR catalysis, we compared the NEM modification levels before and after KO treatment of the wild-type hVKOR and cysteine mutants (Fig. 3, Tables S1 and S2 and Fig. S2).

For the Cys43Ala mutant, 87% of Cys135 is protected from NEM modification after the KO treatment (Fig. 3*A*). The increased protection is consistent with a covalent complex formed between KO and reduced Cys135, which becomes unreactive to NEM after forming this complex. In addition, 93% of Cys51 and 98% Cys132 are protected from NEM modification, suggesting that their major state involves forming a Cys51–Cys132 disulfide bond. An alternative state is that Cys132/Cys135 forms a disulfide bond and Cys135 is not linked to KO (Fig. 3*A*, inset), a state rendering Cys51 free for NEM modification. Because quantitative MS shows that only 7% of Cys51 is free after the KO treatment, this state constitutes a minor cellular fraction of the Cys43Ala mutant. On the other hand, the Cys135–KO complex appears to be a major cellular state, at least under experimental conditions for which KO is provided in large excess. Presence of this major species is consistent with the observation that, in presence of KO, Cys43Ala shows slower electrophoretic mobility through potentially forming a covalent adduct (Fig. 2).

For the wild-type hVKOR, we find that both Cys43 and Cys135 become more protected from NEM modification after the KO treatment (Fig. 3*B*). In the wild-type, the PO state is a major form, and the electron transfer from Cys43 can readily resolve this Cys135-linked intermediate (Fig. 3*B*, inset). Resolving this intermediate results in the formation of the Cys132–Cys135 disulfide. With the excess of KO driving the electron transfer, Cys43/Cys51 also form a disulfide bond. In other words, the two cysteine pairs in wild-type hVKOR become fully oxidized after the KO treatment. Thus, both Cys43 and Cys135 become more protected from NEM modification.

Cys51Ala mutation blocks the electron transfer from Cys43, and this experiment can be viewed as a control. Consistently, we find that Cys43 status remains unchanged after the KO treatment (Fig. 3*C*). Because the Cys51Ala mutation also favors the formation of Cys132–Cys135 disulfide, 83% of Cys135 is NEM protected in the absence of KO. Because most Cys135 is oxidized and unreactive, KO treatment results in an insignificant increase in the protection level of Cys135, indicating nearly no Cys135–KO complex formation. As another control is the observation that the Cys132Ala mutant (Fig. 3*D*) after KO treatment shows also a small increase (∼8.5%) in the Cys135 protection, indicating that a small level of the Cys135-KO complex is being formed. The Cys132Ala mutation is expected to trap this complex because previous models predict that the catalysis by free Cys132/Cys135 proceeds *via* the Cys135–KO intermediate (Fig. 1*A*). The small level of this complex, however, indicates that its formation is unfavored in the Cys132Ala mutant, probably because this mutation breaks the Cys51–Cys132 disulfide that is critical to maintain the substrate binding pocket. Consistently, we did not observe the change of electrophoretic mobility for the Cys132Ala mutant (Fig. 2*A*). Finally, the Cys135Ala mutant is no longer reactive to KO. As expected, KO treatment of Cys135Ala mutant shows basically no change in the status of other catalytic cysteines (Fig. 3*E*). Taken together, these control experiments are consistent with the observations from the electrophoretic mobility assay, reinstating that free Cys135 is required to form the proposed KO covalent complex, as part of the catalytic pathway.

### Formation of the proposed stable intermediate stabilizes the hVKOR protein conformation

**Figure 4:**
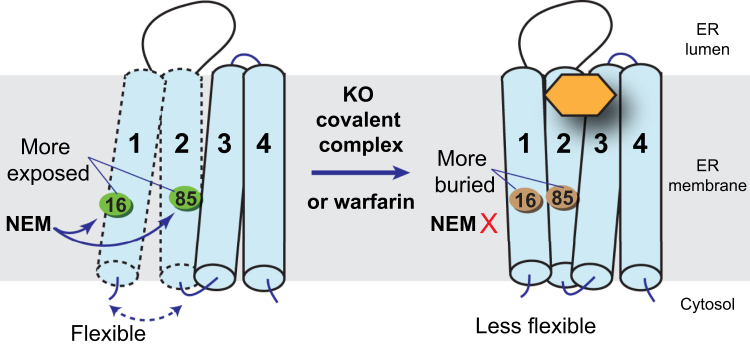
**Formation of the covalent complex reduces conformational flexibility of the hVKOR protein.***Left*, the TMs of hVKOR are flexible in absence of a bound ligand. Owing to this structural flexibility, Cys16 on TM1 and Cys85 on TM2 can be readily labeled by NEM. *Right*, upon warfarin binding or formation of the covalent complex with KO (*orange hexagon*), the TMs become less flexible, and Cys16 and Cys85 become protected from NEM modification.

Besides the four cysteines involved in the catalysis, hVKOR contains three additional cysteines: Cys16, Cys85, and Cys96. We previously found that Cys16 and Cys85 are important reporters for the hVKOR conformational change (13). When the hVKOR structure is stabilized, its transmembrane helices (TMs) become less mobile. Consequently, Cys16 on TM1 and Cys85 on TM2 become less exposed for NEM modification (Fig. 4). We observed that Cys16 and Cys85 are protected from NEM modification after warfarin treatment, indicating that the tightly bound warfarin stabilizes the hVKOR structure (13, 14). Remarkably, we also find that KO treatment of the Cys43Ala mutant results in a similar protection of Cys16 and Cys85 (Fig. 3*A*), suggesting that the reaction intermediate also stabilizes the hVKOR conformation (Fig. 4). Binding of this stable intermediate is a reminiscence of the tight binding of warfarin to hVKOR (20), which has a subnanomolar binding affinity and is essentially irreversible. Given that the K_*M*_ of the KO substrate is known to be in the μM concertation, the Cys43Ala mutant seems to increase the apparent KO affinity by a few magnitudes and to a similar level as warfarin, consistent with the formation of a covalent complex. In contrast, we do not observe increased Cys16 and Cys85 protection in the wild-type or other cysteine mutants (Fig. 3, *B*–*E*). These observations are consistent with the electrophoretic mobility results (Fig. 2 and Table 1), showing that only the Cys43Ala mutation can significantly enrich the potential covalent complex after KO treatment.

**Table 1:** Summary of the redox status, NEM protection, and activity of wild-type hVKOR and cysteine mutants

Protein	WT	C43A	C51A	C132A	C135A
Possible disulfide bonds					
C43–C51	+	-	-	+	+
C51–C132	+	+	-	-	+
C132–C135	+	+	+	-	-
C132/C135 pattern (%)^a^					
O	41.4	45.3	75.8	N/A	N/A
PO	**53.0**^b^	53.1	14.8	N/A	N/A
R	**5.6**	**1.6**	**9.4**	N/A	N/A
Activity (%)^c^	**100**	**8.4**	**77.5**	1.6^d^	1.2^d^
NEM protection −/+ KO (%)					
C43	41.7/76.8	N/A	37.3/37.2	87.1/92.3	40.7/40.7
C51	91.6/94.8	86.4/93.0	N/A	89.4/92.4	93.3/90.4
C132	90.0/92.6	97.3/97.6	80.1/84.5	N/A	66.5/65.0
C135	41.3/77.5	46.5/86.9	83.4/86.4	24.6/33.1	N/A
C96	4.4/6.3	5.9/6.6	7.5/6.1	4.8/5.9	5.4/6.4
C16	7.5/8.4	**9.4/35.7**^b^	7.9/7.54	7.4/10.8	7.6/8.08
C85	18.7/19.5	**21.5/40.9**	19.9/15.5	17.5/17.0	19.5/13.3
Gel mobility shift	No	**Yes**	No	No	No

a Cys132 and Cys135 can be both oxidized (O), both reduced (R), or partially oxidized (PO).

b Shaded areas and bold letters indicate correlation between the PO/R levels and the enzymatic activity, and correlation between the gel mobility shift and the increase of Cys16/Cys85 protection by NEM modification.

c The cellular carboxylation activities of the cysteine mutants are normalized to that of the wild-type hVKOR.

d Baseline activity is observed. There is an additional vitamin K reductase in the double-knockout cells that can convert vitamin K to vitamin K hydroquinone to support carboxylation of the reporter protein (20, 23, 30). This VKR can use the small amount of vitamin K in the fetal bovine serum, which is used for cell culture (21). As a result, the carboxylation assay shows a small level of activity even with the dead mutants of hVKOR.

### Wild-type hVKOR is active in both reduced and partially oxidized states

**Figure 5:**
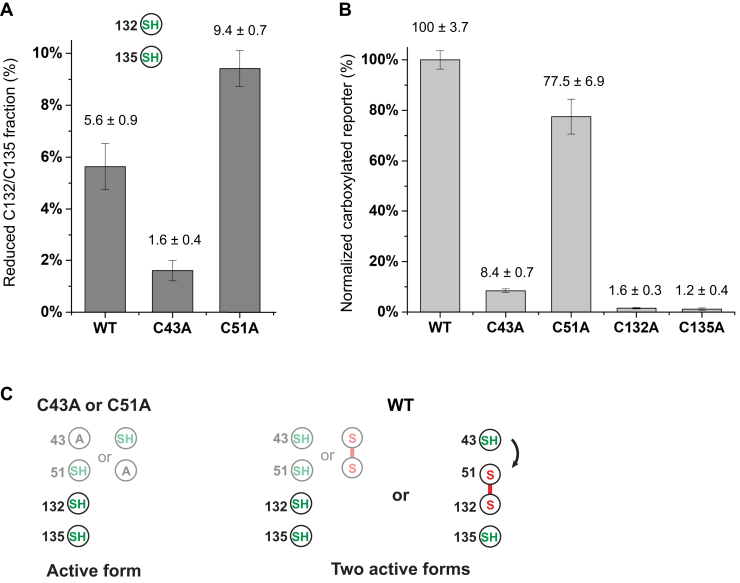
**Correlation between the cellular fraction of reduced Cys132/Cys135 and the cellular activity of hVKOR.***A*, cellular fraction of reduced Cys132/Cys135 in wild-type hVKOR and cysteine mutants. The cellular fraction of reduced Cys132/Cys135 is measured by quantitative MS analyses of peptides containing both cysteines. This fraction cannot be measured for Cys132Ala and Cys135Ala owing to these mutations. *B*, cellular activity of wild-type hVKOR and cysteine mutants. The activity of Cys43Ala and Cys51Ala roughly correlates with their content of reduced Cys132/Cys135 (shown in *A*). Compared with the Cys51Ala mutant, wild-type hVKOR has higher activity but lower content of reduced Cys132/Cys135. *C*, model explaining the cellular activity and cysteine status. Because Cys43Ala and Cys51Ala block the electron transfer *via* Cys43/Cys51, the fraction of reduced Cys132/Cys135 determines the activity of these mutants. In contrast, both the reduced Cys132/Cys135 and the partially oxidized state contribute to the cellular activity of wild-type hVKOR.

To investigate the activity of hVKOR in different redox states, we analyzed the correlation between the cellular activity and cellular cysteine status of wild-type hVKOR and the mutants (Fig. 5, *A*–*B*). For the Cys43Ala mutant, quantitative MS shows that hVKOR in R state constitutes only 1.6% of the total cell fraction (Fig. 5*A*); this state was assumed to be the reactive state in previous studies (10, 11, 12). For the Cys51Ala mutant, the fraction of R state is 9.4% (Fig. 5*A*), which is approximately sixfold greater than that for the Cys43Ala mutant. Remarkably, the cellular activity of Cys51Ala is approximately ninefold greater than Cys43Ala; Cys43Ala and Cys51Ala show 8.4% and 77.5% of the wild-type hVKOR activity (Fig. 5*B*), respectively. This rough correlation (Table 1) argues that the cellular level of R state largely determines the overall activity of these mutants. As a control, the Cys132Ala and Cys135Ala mutants are essentially inactive; their residual activities (1–2%) are within the baseline activity of the carboxylation assay due to other interfering factors (21). Furthermore, although the overall fraction of R state is low, this species can be highly reactive. For instance, R state constitutes merely 9.4% of the cellular fraction of the Cys51Ala mutant, but the activity of this mutant is close (77.5%) to that of the wild-type hVKOR.

More interestingly, in the wild-type hVKOR, the fraction of R state is only 5.6% (Fig. 5*A*). In other words, wild-type hVKOR in the R state is 1.7-fold lower than Cys51Ala mutant in the R state, but the wild-type shows approximately 1.3-fold higher activity (Fig. 5*B*). This discrepancy can be explained by the existence of a second reactive form in wild-type hVKOR. As explained above, the PO state is also capable of complete substrate reduction (Fig. 2*B*). Although less wild-type hVKOR is in the R state, 53% of wild-type is in the PO state (Fig. 1*B*) that can reduce substrate through the electron transfer. In contrast, the PO state is dysfunctional in the Cys43Ala or Cys51Ala mutant because the electron transfer is blocked (Fig. 5*C*).

## Discussion

In this study, we have thoroughly analyzed the hVKOR catalysis under a cellular setting, complementing previous theories of the catalytic chemistry (10, 11, 12). These previous chemical modeling and quantum mechanics studies assumed that the catalysis of hVKOR starts from the R state. Our quantitative MS results, however, show that this state constitutes only a minor cellular fraction (5.6%) of the wild-type hVKOR. Instead, most cellular hVKOR forms (53%) have their active site in the PO state, in which only Cys135 is reduced, and the KO reduction requires electron transfer from Cys43 (Fig. 2*B*). The catalytic activity of this PO state, however, has not been demonstrated before, and its contribution to the overall cellular activity of hVKOR was unknown.

**Figure 6:**
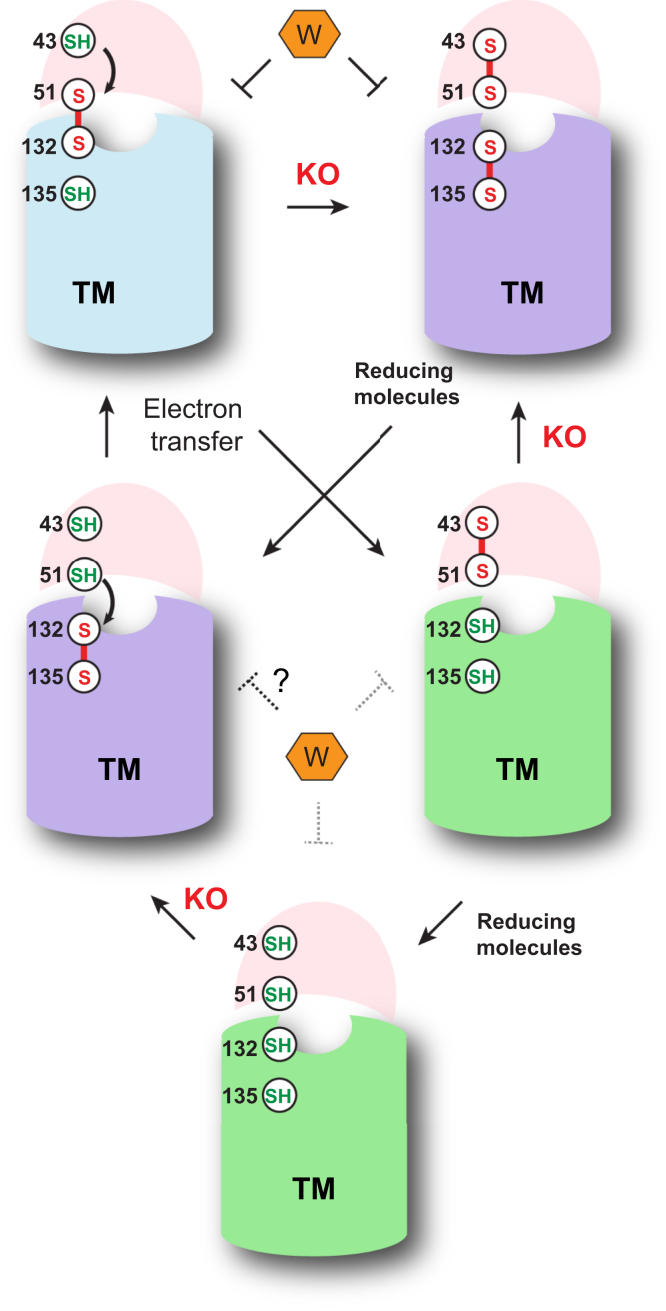
**Mechanisms of hVKOR catalysis and warfarin inhibition in the cellular environment.** The TM barrel is shown in *green*, *blue*, and *purple* for the hVKOR active site in R, PO, and O states, respectively. These states can be interconverted by electron-transfer steps *via* thiol-disulfide relays (*curved arrows*), by oxidation after reacting with KO, or by reduction with the involvement of other molecules. Identity of the reducing molecules remains unclear, which can be either partner proteins of hVKOR (29) or small molecules abundant in the ER, such as reduced glutathione (20). The hVKOR catalysis with KO and warfarin (W) inhibition occur at different redox states. The KO reduction is carried out with R and PO states, but not in O state. In contrast, warfarin preferably inhibits the PO and O states. The inhibition of the R state is known to be weak (13, 27). Because KO and warfarin target different states, warfarin shows mixed inhibition kinetics (16).

To resolve these issues of hVKOR catalysis, we analyzed the cysteine status of wild-type hVKOR and cysteine mutants in cells (Table 1). Using the Cys43Ala and Cys51Ala mutants allowed us to examine the activity of the R state, because these mutants abolish the activity of hVKOR in the PO state that requires electron transfer (Fig. 2*B*). With the electron transfer blocked, the relative abundance of R state in these mutants determines their cellular activity. In contrast, the total activity of wild-type hVKOR comes from both R and PO states (Fig. 5*C*). The relative contribution of these states can be approximated. The Cys51Ala activity is solely from R state, and this mutant has 77.5% of the wild-type hVKOR activity. For the wild-type, the fraction of R state is approximately 60% of Cys51Ala mutant. Therefore, the R state contributes to about 46% of the total activity of wildtype. The remaining 54% wild-type activity comes from its PO state with reduced Cys43/Cys135. These relative activities of R and PO states correspond to their 5.6% and 53% cellular fraction, respectively. Thus, the R state has approximately eightfold higher specific activity than the PO state. Taken together, although the R state is of low abundance, it is more active than the PO state in the cellular environment. This behavior can be understood because, in the R state, the electron is directly passed from Cys132 to resolve the Cys135–KO reaction intermediate (Fig. 1*A*). In contrast, in the PO state, a protein conformational change seems to be required for Cys43 to pass the electron to the Cys51–Cys132 disulfide (Fig. 2*B*). The reduced Cys132 in turn resolves the Cys135–KO covalent intermediate. This process is less efficient because it takes additional electron-transfer steps and requires a protein conformational change. Taken together, reduction of KO by hVKOR can initiate with either an R or a PO state (Fig. 6), and these states contribute about equally to the hVKOR activity in a cellular environment.

Whether the activity of hVKOR is maintained through the electron-transfer pathway has puzzled the field for years (8, 9, 13, 22, 23, 24, 25). Although Cys43 and Cys51 are predicted to mediate the electron transfer, only Cys43Ala abolishes the hVKOR activity in the cellular assay, whereas Cys51Ala shows largely retained activity. Here we find that this apparent paradox is because, compared with Cys43Ala, Cys51Ala induces the formation of much more R form (Fig. 5*A*). In other words, the high activity of Cys51Ala simply comes from the raised level of R form, regardless of the electron-transfer pathway that is blocked in both mutants. Moreover, the higher cellular fraction of R form in the Cys51Ala mutant may reflect an alternative electron-transfer process that bypasses Cys43 and Cys51. This bypassing may be artificially enhanced because small reducing molecules, such as reduced glutathione that is abundant in the ER (20), may gain direct access to Cys132 when Cys51 is mutated. This mutation breaks the Cys51–Cys132 disulfide bond that anchors a loop region to the transmembrane domain; according to our structural prediction, this region forms a cap covering the active site (13, 19), and breaking the stabilizing disulfide may render the Cys132 artificially exposed for small reducing molecules. In the wild-type hVKOR, however, the fraction of R form is only 5.6%, and a large part of this can be generated also by electron transfer from Cys43/Cys51. Thus, the contribution of the bypassing pathway is expected to be minor in the wild-type hVKOR.

Evidences for a potential covalent Cys135–KO complex suggest that this is a necessary step for hVKOR to catalyze KO reduction. This covalent complex is enriched for the Cys43Ala mutation, indicating that blocking the electron transfer from Cys43 disrupts a major reactive pathway that proceeds through the PO state, which contributes to about half of the wild-type hVKOR activity according to our above analysis. Taken together, the electron transfer through Cys43/Cys51 dominates the cellular activity of hVKOR.

Interestingly, we find that formation of the covalent complex reduces the structural flexibility of hVKOR, similar to the effect induced by warfarin binding (Fig. 4). Indeed, warfarin binding, although not forming a covalent bond, is known to be nearly irreversible (20, 26), much similar to the effect of the covalent bond being formed with the reaction intermediate. This similarity suggests that warfarin binding may mimic that of the covalent reaction intermediate, and these two events may induce a similar change in the hVKOR protein conformation. Warfarin is known to inhibit oxidized hVKOR including the PO and O forms (13, 27). Warfarin, however, poorly inhibits hVKOR in the R state (Fig. 6) (13, 27). In contrast, hVKOR catalysis is achieved by the R and PO states. Therefore, warfarin should be competitive with substrates at the PO state, but not competitive at the O or R states. This difference explains the previous observation that warfarin is a mixed inhibitor showing a combination of noncompetitive and competitive kinetics (15, 16).

In sum, hVKOR catalysis in cells involves two cellular redox states (R and PO) that contribute about equally to the overall hVKOR activity (Fig. 6). Furthermore, we provide experimental evidences that KO reduction may involve a stable covalent intermediate, whose binding is resembled by warfarin binding. Unlike hVKOR catalysis, warfarin inhibition preferably targets oxidized states (PO and O) but not the R state, explaining its mixed inhibition kinetics. Our study clarifies the catalytic mechanism of hVKOR in the cellular environment by a combination of biochemistry with quantitative MS, a method broadly applicable to study other oxidoreductases.

## Experimental procedures

### Electrophoretic mobility assay

Wild-type and mutant hVKOR proteins, with a C-terminal flag tag and ER retention signal, were expressed using stable cell lines (13). The cells were grown in six-well plates until 90% confluence, and KO (10 μM final concentration) was added to the medium. After 5 h incubation, the cells were washed once with ice-cold PBS. Subsequently, 120 μl of labeling buffer (20 mM NEM, 10 μM KO, 1% Triton X-100, 150 mM NaCl, 50 mM Tris-HCl pH 7.5, and protease inhibitor cocktail) was added to each well. The plates were left on ice with shaking for 20 min. Subsequently, the cell lysate was collected and centrifuged at 12,000*g* for 15 min at 4 °C. The supernatant was applied to SDS-PAGE under nonreducing or reducing (with DTT, 100 mM) conditions. Western blot was carried out with the use of anti-flag M2 antibody (Sigma).

### Preparation of sample of KO treatment for MS quantification

Samples for MS quantification were prepared as previously described (13, 14). Cells stably expressing wild-type and mutant hVKOR were grown on 15 cm plates, and each MS analysis required the number of cells from approximately five plates. At 90 to 100% cell confluence, KO (10 μM final concentration) was added to the medium and incubated with the cells for 5 h. The cells were washed once with ice-cold PBS buffer. Subsequently, 1.8 ml labeling buffer (20 mM NEM, 1% Triton X-100, 150 mM NaCl, 50 mM Tris-HCl pH 7.5, and protease inhibitor cocktail) was added. The plates were left on ice for 20 min with frequent shaking to facilitate the NEM reaction with free cysteines. The cell lysate was collected in a new tube, and 1 M DTT (100 mM final concentration) was added to quench the NEM reaction. This mixture was centrifuged at 12,000*g* for 15 min at 4 °C. The supernatant was loaded onto a G-25 desalting column to remove DTT and NEM. The wild-type or mutant hVKOR proteins were purified with anti-flag M2 resin (Sigma), precipitated by trichloroacetic acid, and applied to reducing SDS-PAGE. The hVKOR protein band was excised for in-gel digestion.

### In-gel digestion and LC-MS/MS analysis

In-gel digestion and LC-MS/MS analysis were performed as previously described (13). Briefly, hVKOR was in-gel reduced with 5 mM tris-(2-carboxyethyl) phosphine (TCEP) for 30 min at 56 °C. After acetone washing, the gel pieces were incubated with 10 mM NEM-*d*_*5*_ for 60 min at room temperature to label the cysteines not blocked by NEM. The isotopically labeled protein was subjected to in-gel digestion by chymotrypsin. The peptides released from the in-gel digestion were directly used for LC-MS/MS analysis.

The chymotryptic peptides were analyzed by using an Eksigent 2D nano LC system (Eksigent Technologies) and a Thermo LTQ Orbitrap XL mass spectrometer. The parameters were modified from those described previously (13). Briefly, the peptides were loaded onto a trap column (Acclaim PepMap100, 100 μm × 2 cm, C18, 5 μm, 100 Å, Thermo Scientific Dionex) at a flow rate of 4.5 μl/min with 0.1% formic acid in water. Subsequently, the peptides were separated on a reversed-phase column (75 μm × 180 mm) custom-packed by using Michrom Magic C18 material (5 μm particle size and 200 Å pore size). An LC gradient of solvent B (0.1% formic acid in acetonitrile) was delivered at a flow rate of 260 nl/min for 0 to 10% in 5 min and 10 to 40% in 95 min. Mass spectra (*m/z* range 350–2000) of the eluted peptides were acquired at high mass resolving power (50,000 for ions of *m/z* 400). The seven most abundant ions were selected for fragmentation by collision-induced dissociation in the linear ion trap without charge state rejection.

### Peptide identification and quantification measurements

The raw MS data were searched by Mascot MS/MS ions search against a customer database containing the hVKOR sequence. The search parameters were set as the following. Taxonomy: *Homo sapiens*; enzyme: no enzyme cleavage specificity; allow up to 0 missed cleavages; variable modifications: NEM (Cys), NEM+water (Cys), NEM-*d*_*5*_ (Cys), NEM-*d*_*5*_+H_2_O (Cys), Cys-mutation-Ala; peptide tolerance ± 15 ppm; # ^13^C: 0; MS/MS tolerance ± 0.8 Da; data format: Mascot generic; instrument: ESI TRAP.

Cysteine-containing peptides with an identification score of 20 and higher were selected for quantification measurements, and the product-ion spectra were manually validated. All the MS and MS/MS spectra of such peptides are summarized in Fig. S2. Peak areas of the two versions of the same peptide differentially labeled with NEM and NEM-*d5* were analyzed by the extracted ion chromatogram (XIC) for the corresponding *m/z* in Thermo Xcalibur Qualbrowser (Tables S1 and S2 and Supplementary Excel File). For peptides containing one cysteine, the NEM protection level of each peptide was calculated with the peak area of NEM-*d*_5_ modified peptide divided by the summed peak areas of the NEM and NEM-*d*_5_ modified peptides (*i.e.*, NEM-*d*_5_/(NEM-*d*_5_ + NEM)). For peptides containing Cys132/Cys135, the fractions of different states are calculated with the peak areas in the following ways: R state = 2NEM/(2NEM + NEM&NEM-*d*_*5*_ + 2NEM-*d*_*5*_); PO state = NEM&NEM-*d*_*5*_/(2NEM + NEM&NEM-*d*_*5*_ + 2NEM-*d*_*5*_); O state = 2NEM-*d*_*5*_/(2NEM + NEM&NEM-*d*_*5*_ + 2NEM-*d*_*5*_), in which 2NEM, NEM&NEM-*d*_*5*_, and 2NEM-*d*_*5*_ stand for that Cys132/Cys135 are both labeled by NEM, one labeled by NEM and another by NEM-*d*_*5*_, and both labeled by 2NEM-*d*_*5*_, respectively. All the data can be found in Table S2 and the Supplementary Excel File.

### Activity assay

Assays of the hVKOR activity were performed as previously described^11^ using a cell line established with a chimeric FIXgla-Protein C gene as the γ-carboxylation reporter and with both *VKOR* and *VKORL* genes knocked out. Wild-type and mutant hVKOR clones along with a luciferase gene were transfected into this double-knockout cell line. The carboxylation level of secreted FIXgla-PC was measured by a sandwich ELISA by using the cell-culture medium, with luciferase activity serving as an internal control for transfection efficiency. In this double-knockout cell line, there is an additional vitamin K reductase that can convert vitamin K to vitamin K hydroquinone to support carboxylation of the reporter protein (21). This vitamin K reductase can use the small amount of vitamin K in the fetal bovine serum used for cell culture. As a result, there is a small level of basal activity (∼1%) in this carboxylation reporter assay.

## Data availability

The mass spectrometry data have been deposited to the ProteomeXchange Consortium through PRIDE partner repository (28) (dataset identifier: PXD020676). All other relevant data are contained within the article and accompanying supporting information.

## Conflict of interest

The authors declare that they have no conflicts of interest with the contents of this article.

## Supporting information

Supplementary Figures and Tables

Supplementary Excel File
