# Supplementary material for: The catalytic mechanism of vitamin K epoxide reduction in a cellular environment
Source: J Biol Chem. 2020 Dec 10;296:100145. doi: 10.1074/jbc.RA120.015401 (PMC7895805; doi:10.1074/jbc.RA120.015401)

**SUPPORTING INFORMATION APPENDIX**

**The catalytic mechanism of vitamin K epoxide reduction in a cellular environment**

Guomin Shen^1,2†^, Weidong Cui^3^, Qing Cao^1^, Meng Gao^1^, Hongli Liu^1^, Gaigai Su^1^, Michael L. Gross^3^, Weikai Li^2†^

^1^ Institute of Homeostasis and Thrombosis, School of Basic Medical Science, Henan University of Science and Technology, Luoyang, Henan 471023, P. R. China.

^2^ Department of Biochemistry and Molecular Biophysics, Washington University School of Medicine, St. Louis, MO 63110, USA.

^3^ Department of Chemistry, Washington University in St. Louis, St. Louis, MO 63130, USA.

† Correspondence authors:

Guomin Shen, Email: shenba433@163.com or shenguomin@haust.edu.cn

Weikai Li, E-mail: [weikai@wustl.edu](mailto:weikai@wustl.edu)

*Running title: Mechanism of vitamin K epoxide reduction in cells*

**LIST OF SUPPORTING INFORMATION MATERIAL**

- **Table S1.** Summary of assigned peptides using Mascot™.
- **Table S2.** Representative quantification measurements for peptides containing one or two cysteines. Quantification measurements of all samples and repeats are reported in a separate excel file.
- **Figure S1.** The electron-transfer pathway of hVKOR in cells.
- **Figure S2.** MS and MS/MS spectra of the cysteine-containing peptides.

**Table S1. Summary of assigned peptides using Mascot™.**

| **Peptide** | **Peptide Sequence** | ***m/z*** | ***z*** | **Score** | **Modification** |
| --- | --- | --- | --- | --- | --- |
| 6 – 22 | GSPGWVRLAL**C**LTGLVL | 940.5271 | 2 | 36 | NEM |
| 6 – 22 | GSPGWVRLAL**C**LTGLVL | 943.0427 | 2 | 20 | NEM-*d_5_* |
| 14 – 20 | AL**C**LTGL | 815.4332 | 1 | 26 | NEM |
| 14 – 20 | AL**C**LTGL | 820.4646 | 1 | 26 | NEM-*d_5_* |
| 14 – 20 | AL**C**LTGL | 833.4437 | 1 | 22 | NEM+H_2_O |
| 14 – 20 | AL**C**LTGL | 838.4751 | 1 | 22 | NEM-*d_5_*+H_2_O |
| 16 – 22 | **C**LTGLVL | 843.4645 | 1 | 39 | NEM |
| 16 – 22 | **C**LTGLVL | 848.4959 | 1 | 35 | NEM-*d_5_* |
| 6 – 25 | GSPGWVRLAL**C**LTGLVLSLY | 1122.117 | 2 | 22 | NEM |
| 6 – 25 | GSPGWVRLAL**C**LTGLVLSLY | 1124.6324 | 2 | 20 | NEM-*d_5_* |
| 14 – 22 | AL**C**LTGLVL | 1027.5857 | 1 | 28 | NEM |
| 14 – 22 | AL**C**LTGLVL | 1032.6171 | 1 | 12 | NEM-*d_5_* |
| 16 – 20 | CLTGL | 631.3120 | 1 | 25 | NEM |
| 16 – 20 | CLTGL | 636.3434 | 1 | 21 | NEM-*d_5_* |
| 40 – 48 | RAL**C**DVGTA | 515.7532 | 2 | 52 | NEM |
| 40 – 48 | RAL**C**DVGTA | 518.2689 | 2 | 53 | NEM-*d_5_* |
| 40 – 48 | RAL**C**DVGTA | 524.7585 | 2 | 37 | NEM+H_2_O |
| 40 – 48 | RAL**C**DVGTA | 527.2742 | 2 | 37 | NEM-*d_5_*+H_2_O |
| 40 – 49 | RAL**C**DVGTAI | 572.2952 | 2 | 44 | NEM |
| 40 – 49 | RAL**C**DVGTAI | 574.8109 | 2 | 42 | NEM-*d_5_* |
| 40 – 49 | RAL**C**DVGTAI | 581.3005 | 2 | 48 | NEM+H_2_O |
| 40 – 49 | RAL**C**DVGTAI | 583.8162 | 2 | 57 | NEM+H_2_O |
| 43 – 48 | **C**DVGTA | 690.2763 | 1 | 29 | NEM+H_2_O |
| 43 – 48 | **C**DVGTA | 695.3077 | 1 | 29 | NEM-*d_5_*+H_2_O |
| 48 – 55 | AIS**C**SRVF | 504.2529 | 2 | 22 | NEM |
| 48 – 55 | AIS**C**SRVF | 506.7685 | 2 | 36 | NEM-*d_5_* |
| 48 – 55 | AIS**C**SRVF | 513.2581 | 2 | 20 | NEM+H_2_O |
| 48 – 55 | AIS**C**SRVF | 515.7738 | 2 | 23 | NEM-*d_5_*+H_2_O |
| 49 – 55 | IS**C**SRVF | 468.7343 | 2 | 24 | NEM |
| 49 – 55 | IS**C**SRVF | 471.2500 | 2 | 33 | NEM-*d_5_* |
| 49 – 55 | IS**C**SRVF | 480.2553 | 2 | 36 | NEM-*d_5_*+H_2_O |
| 50 – 55 | S**C**SRVF | 412.1923 | 2 | 22 | NEM |
| 50 – 55 | S**C**SRVF | 414.7079 | 2 | 30 | NEM-*d_5_* |
| 50 – 55 | S**C**SRVF | 421.1975 | 2 | 20 | NEM+H_2_O |
| 50 – 55 | S**C**SRVF | 423.7132 | 2 | 23 | NEM-*d_5_*+H_2_O |
| 84 – 88 | G**C**IFY | 727.3120 | 1 | 20 | NEM |
| 84 – 88 | G**C**IFY | 732.3434 | 1 | 21 | NEM-*d_5_* |
| 89 – 97 | TLQLLLG**C**L | 1098.6228 | 1 | 39 | NEM |
| 89 – 97 | TLQLLLG**C**L | 1103.6542 | 1 | 38 | NEM-*d_5_* |
| 130 – 134 | DF**C**IV | 721.3226 | 1 | 22 | NEM-*d_5_* |
| 130 – 134 | DF**C**IV | 726.3540 | 1 | 22 | NEM-*d_5_* |
| 135 – 139 | **C**ITTY | 725.3175 | 1 | 22 | NEM |
| 135 – 139 | **C**ITTY | 730.3489 | 1 | 20 | NEM-*d_5_* |
| 130 – 135 | DF**C**IV**C** | 954.4107 | 1 | 20 | NEM; NEM-*d_5_* |
| 130 – 136 | DF**C**IV**C**I | 1067.4948 | 1 | 24 | NEM; NEM-*d_5_* |
| 130 – 137 | DF**C**IV**C**IT | 584.7752 | 2 | 33 | NEM; NEM-*d_5_* |

**Table S2. Representative quantification measurements for peptides containing one or two cysteines.** Quantification measurements of all samples and repeats are reported in a separate Excel file. The wildtype hVKOR without KO treatment is used as an example here. For each peptide selected for quantification measurements, the cutoff identification score is 20 and the product-ion spectra were manually validated. Extracted ion chromatogram of each peptide *m/z* was analyzed to obtain the peak area.

| **Residue** | **Peptide sequence** | | **Peak Area** | | | | | | | | | |
| --- | --- | --- | --- | --- | --- | --- | --- | --- | --- | --- | --- | --- |
|  |  |  | **M+NEM** | | **M+NEM+H_2_O** | | **M+NEM*-d_5_*** | **M+ NEM*-d_5_*+H_2_O** | | | **% protection ^a^** | |
| Cys16 | GSPGWVRLALCLTGLVL (6-22) | | 3292783 | | n.a | | 109316 | n.a | | | 3.2 | |
|  | ALCLTGL (14-20) | | 1.12E+09 | | 3.39E+08 | | 2.19E+08 | 21144637 | | | 13.8 | |
|  | CLTGLVL (16-22) | | 3.93E+08 | | n.a | | 22979691 | n.a | | | 5.5 | |
|  | GSPGWVRLALCLTGLVLSLY (6-25) | | 1755893 | | n.a | | 459204 | n.a | | | 20.2 | |
|  | ALCLTGLVL (14-22) | | 1.4E+08 | | 10209142 | | 11006952 | 356893 | | | 6.6 | |
|  | CLTGL (16-20) | | 8.34E+08 | | 56170318 | | 75688120 | 3029520 | | | 8.1 | |
| Cys43 | RALCDVGTA (40-48) | | 2.67E+08 | | 87548185 | | 2.38E+08 | 46925006 | | | 44.6 | |
|  | RALCDVGTAI (40-49) | | 4.3E+08 | | 1.64E+08 | | 3.79E+08 | 81529776 | | | 43.7 | |
|  | CDVGTA (43-48) | | 91233622 | | n.a | | 64251867 | n.a | | | 41.3 | |
| Cys51 | RALCDVGTA (40-48) | | 18770748 | | 6868521 | | 1.86E+08 | 60135759 | | | 90.6 | |
|  | RALCDVGTAI (40-49) | | 59420218 | | 22843431 | | 5.54E+08 | 2.41E+08 | | | 90.6 | |
|  | CDVGTA (43-48) | | 67536207 | | 27898357 | | 6.8E+08 | 2.89E+08 | | | 91.0 | |
| Cys85 | GCIFY (84-88) | | 9.12E+08 | | n.a | | 2.1E+08 | n.a | | | 18.7 | |
| Cys96 | TLQLLLGCL (89-97) | | 57214715 | | n.a | | 3475965 | n.a | | | 5.73 | |
| Cys132 | DFCIV (130-134) | | 7453193 | | n.a | | 77196051 | n.a | | | 91.2 | |
| Cys135 | CITTY (135-139) | | 27002597 | | n.a | | 17923351 | n.a | | | 39.9 | |
| **Residue** | **Peptide sequence** | **Peak area** | | | | | | | **State (%)** | | | |
|  |  | **M+2NEM** | | **M+NEM+NEM-*d_5_*** | | **M+2NEM-*d_5_*** | | | **R** | **PO** | | **O** |
| Cys132/  Cys135 | DFCIVC (130-135) | 2404166 | | 21556153 | | 16509856 | | | 5.94 | 53.26 | | 40.80 |
|  | DFCIVCI (130-136) | 786995 | | 6835996 | | 5207193 | | | 6.13 | 53.28 | | 40.59 |
|  | DFCIVCIT (130-137) | 1605952 | | 16652684 | | 13687583 | | | 5.03 | 52.13 | | 42.85 |

^a^ % of protection of one cysteine residue is calculated from the peak areas using the following equation: ((M+NEM-*d_5_*) + (M+NEM-*d_5_*+H_2_O)) / ((M+NEM) + (M+NEM+H_2_O) + ((M+NEM-*d_5_*) + (M+NEM-*d_5_*+H_2_O)).

^b^ Fractions of Cys132/Cys135 in different states are calculated from the peak areas using the following equations.

R state = (M+2NEM) / ((M+2NEM) + (M+NEM+NEM-*d_5_*) + (M+2NEM-*d_5_*)).

PO state = (M+NEM+NEM-*d_5_*) / ((M+2NEM) + (M+NEM+NEM-*d_5_*) + (M+2NEM-*d_5_*)).

O state = (M+2NEM-*d_5_*) / ((M+2NEM) + (M+NEM+NEM-*d_5_*) + (M+2NEM-*d_5_*)).

In which:

M+2NEM: Cys132/Cys135 both labeled by NEM.

M+NEM+NEM-*d_5_*: Cys132/Cys135 one labeled by NEM and another by NEM-*d_5_*.

M+2NEM-*d_5_*: Cys132/Cys135 both labeled by NEM-*d_5_*,

**
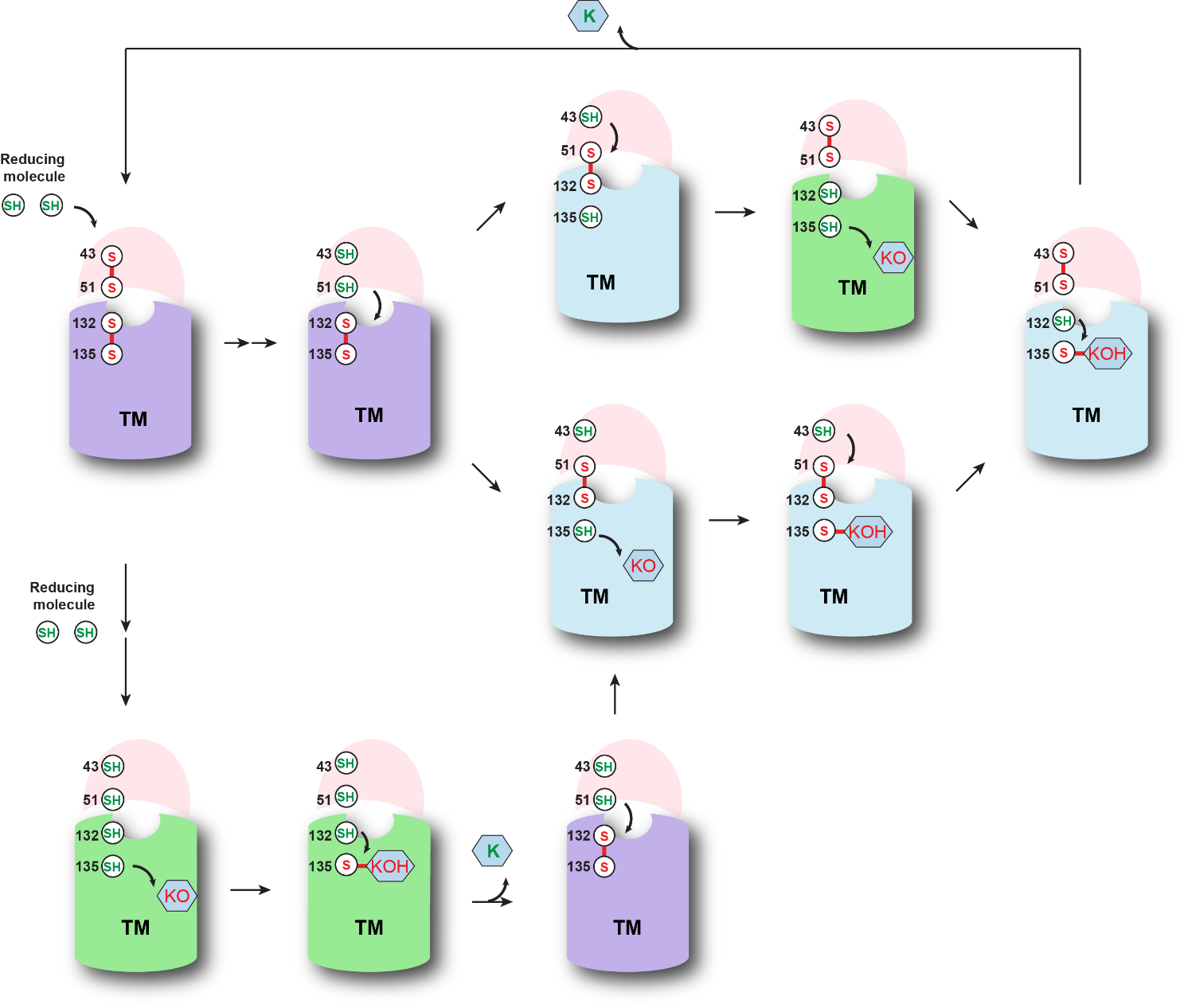
**

**Figure S1. The electron-transfer pathway of hVKOR in cells.** The TM barrel is shown in green, blue and purple for the hVKOR active site in fully reduced, partially oxidized and fully oxidized state, respectively (as in Fig. 6). Curved arrows indicate electron transfer. Only representative pathways are shown here, and other redox states may exist during the electron-transfer process in cells.

**Fig. S2. MS and MS/MS spectra of the cysteine-containing peptides.**


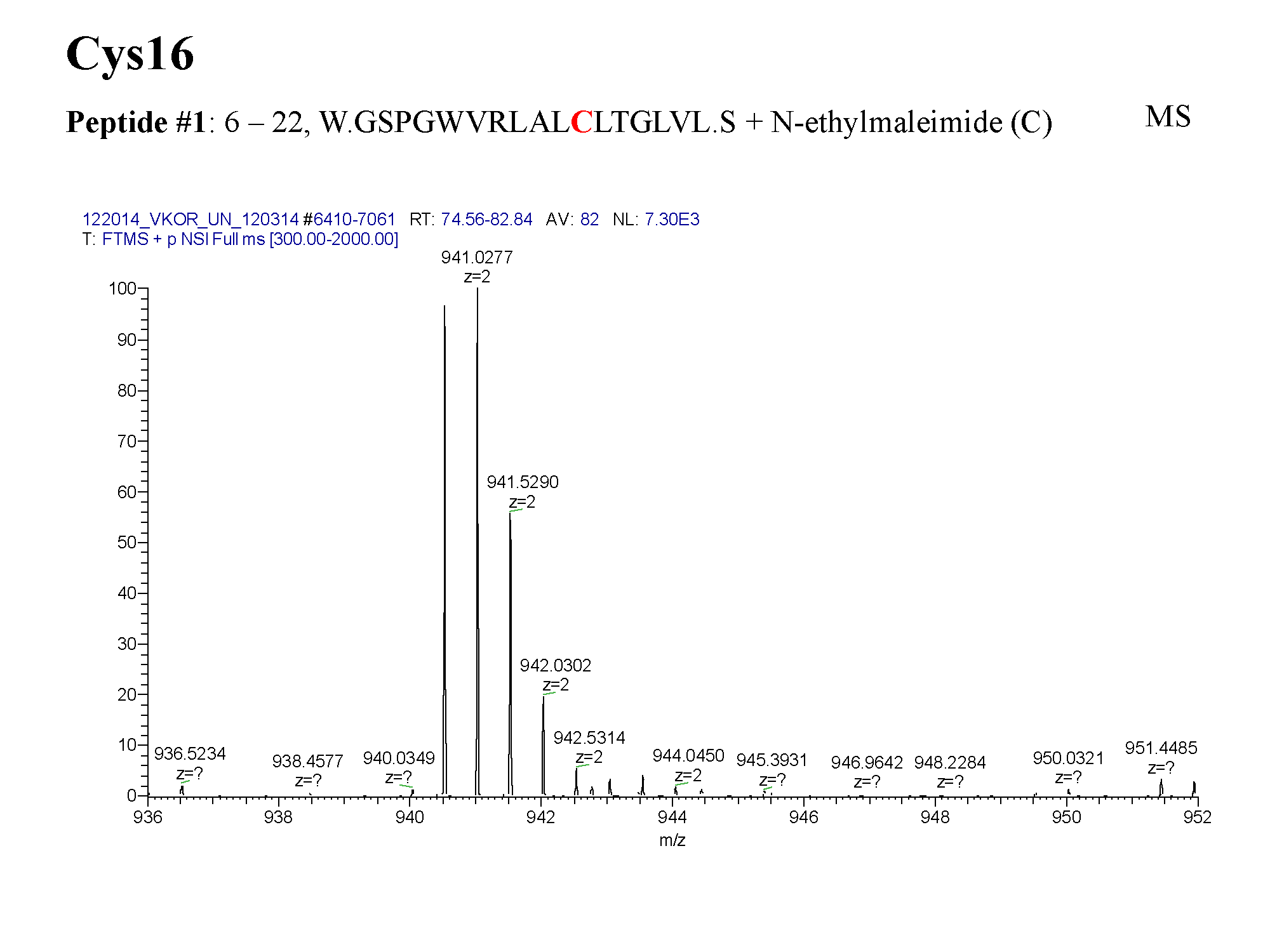

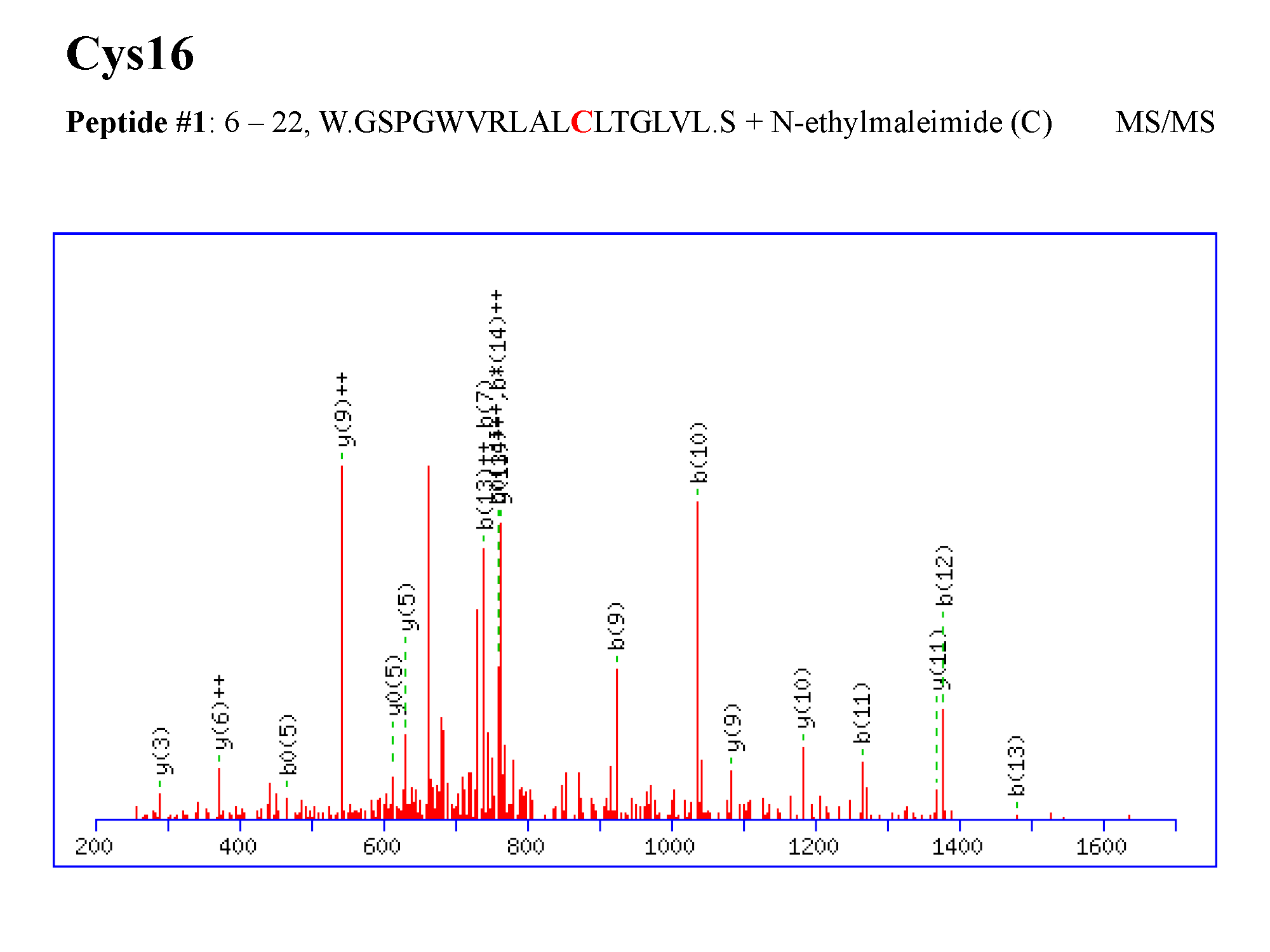

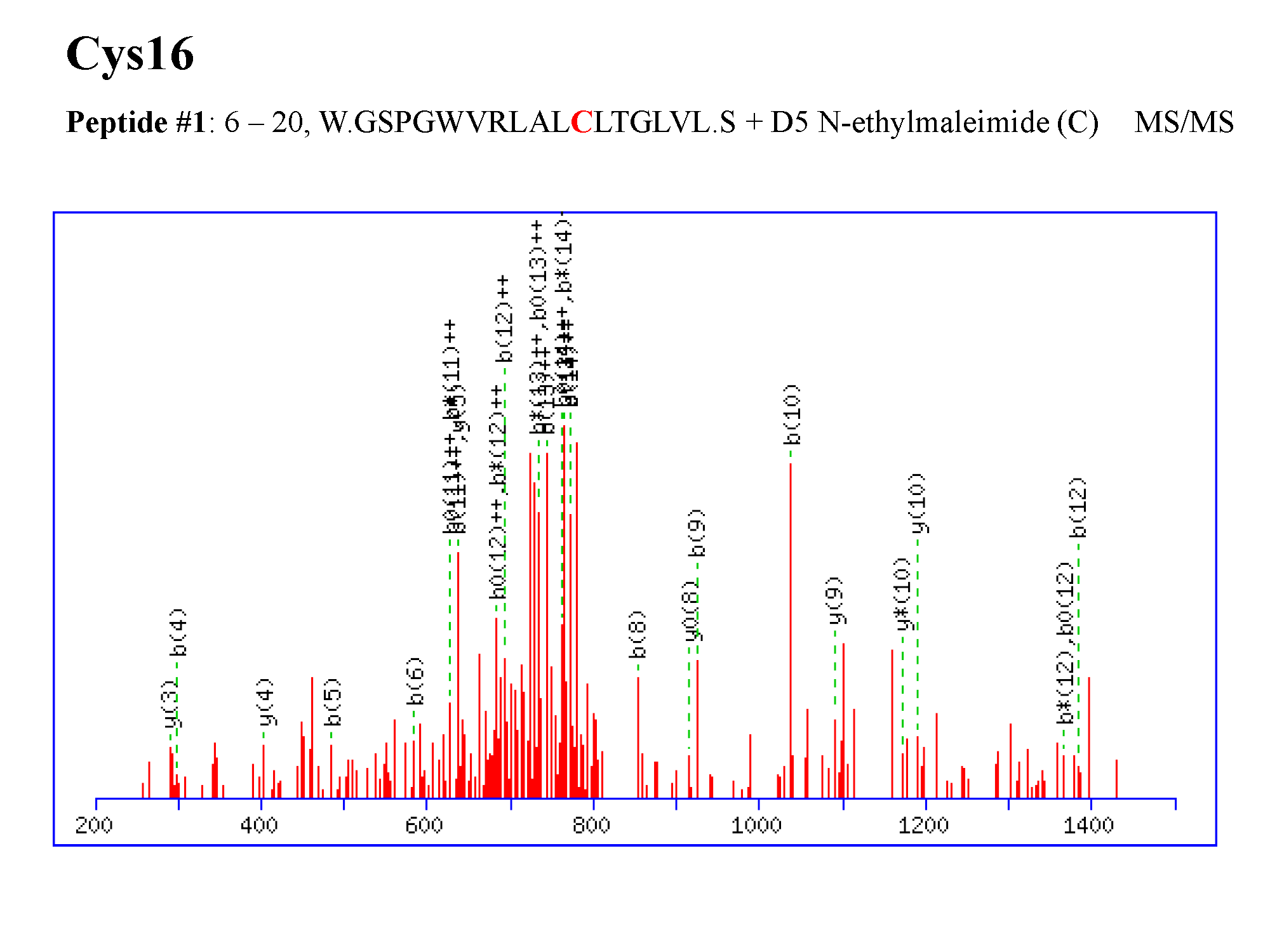

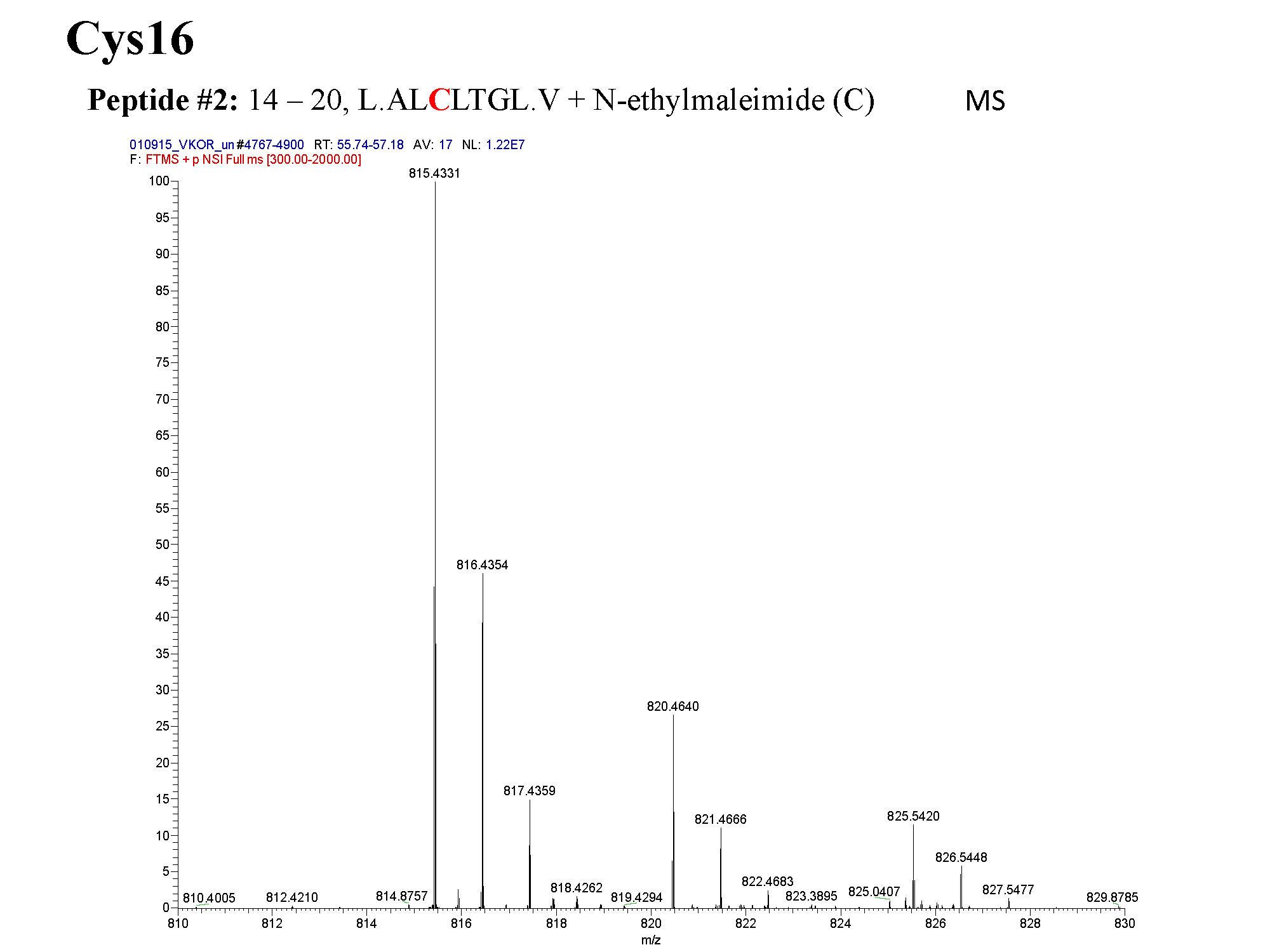

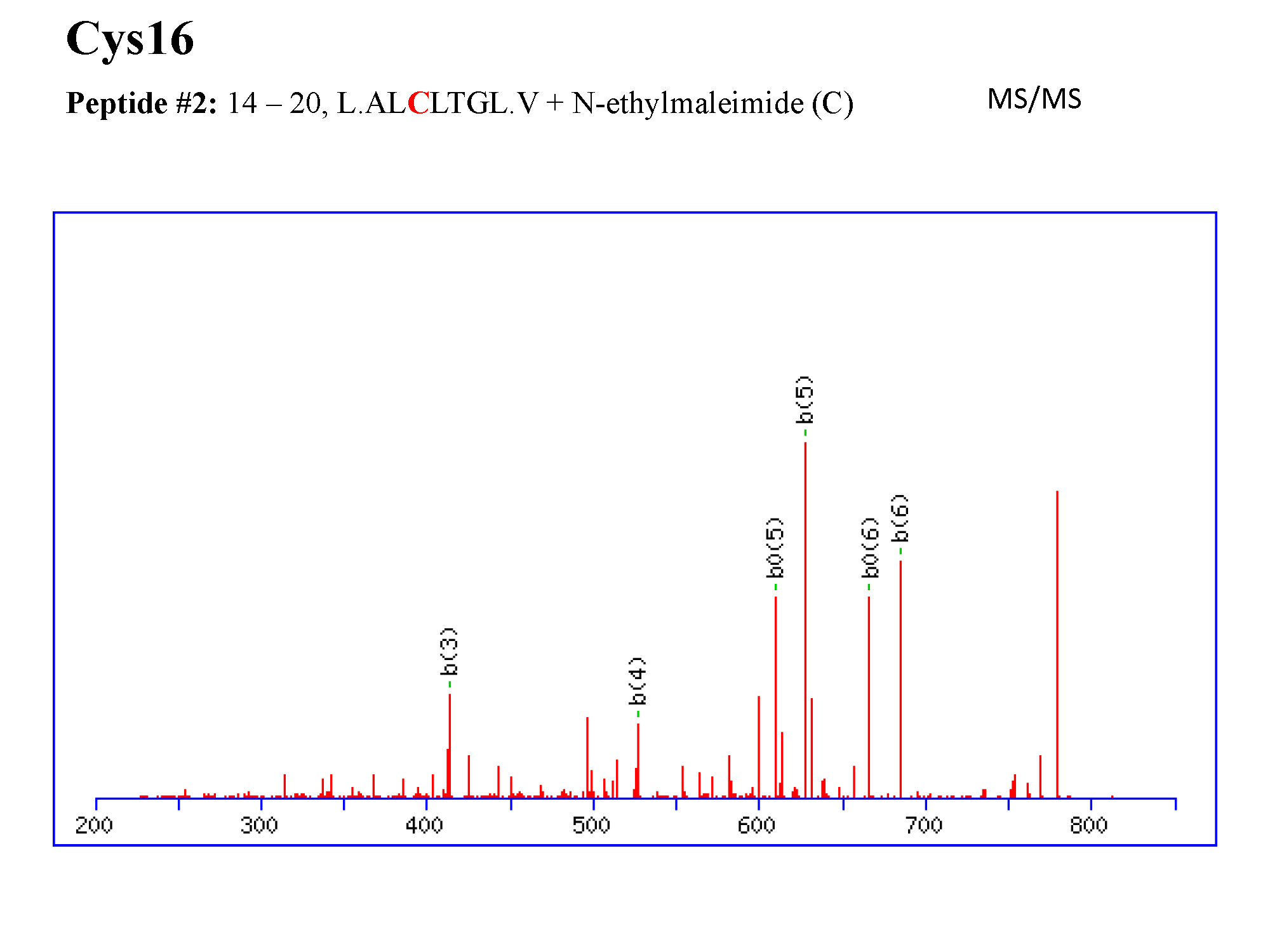

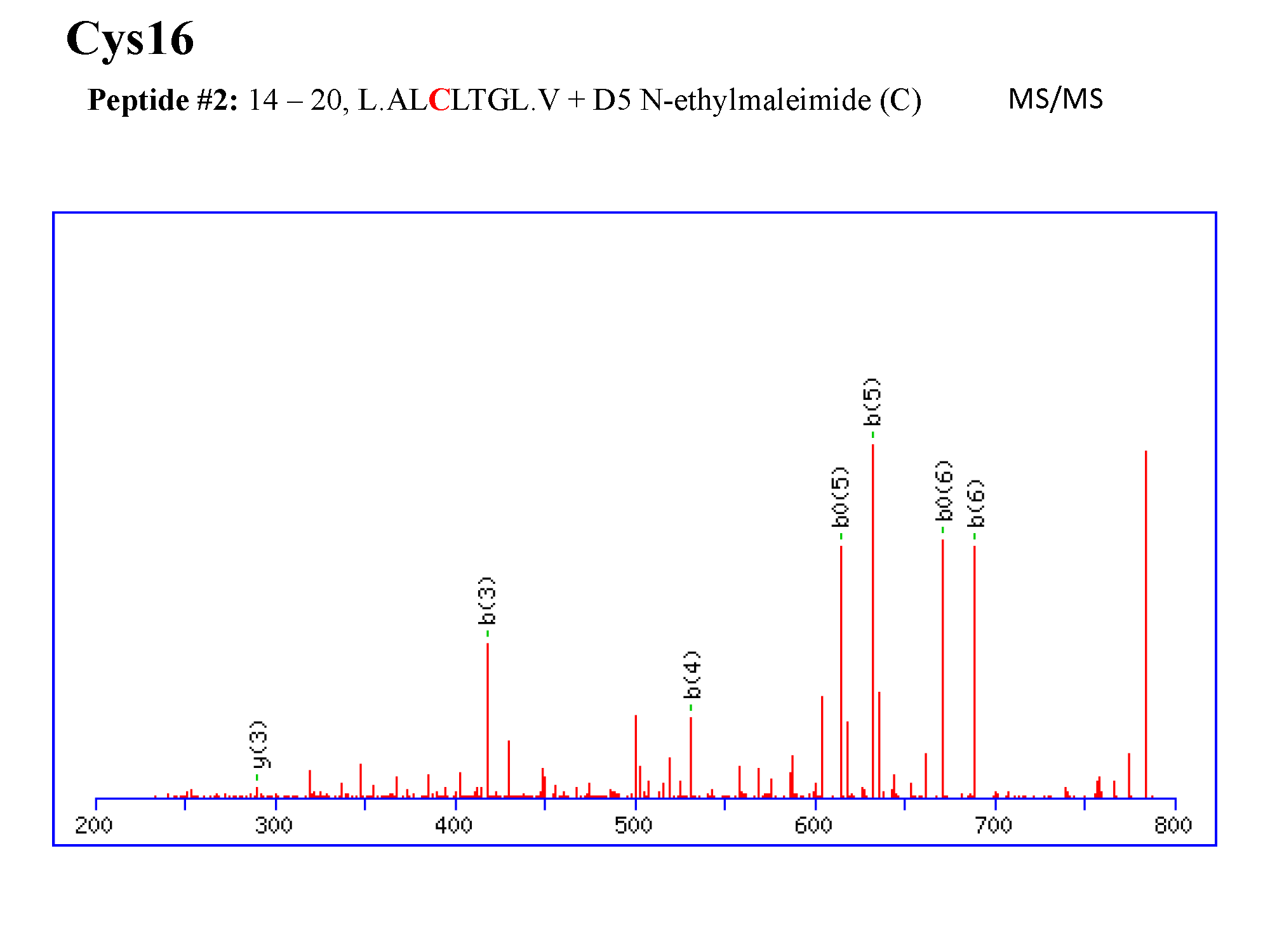

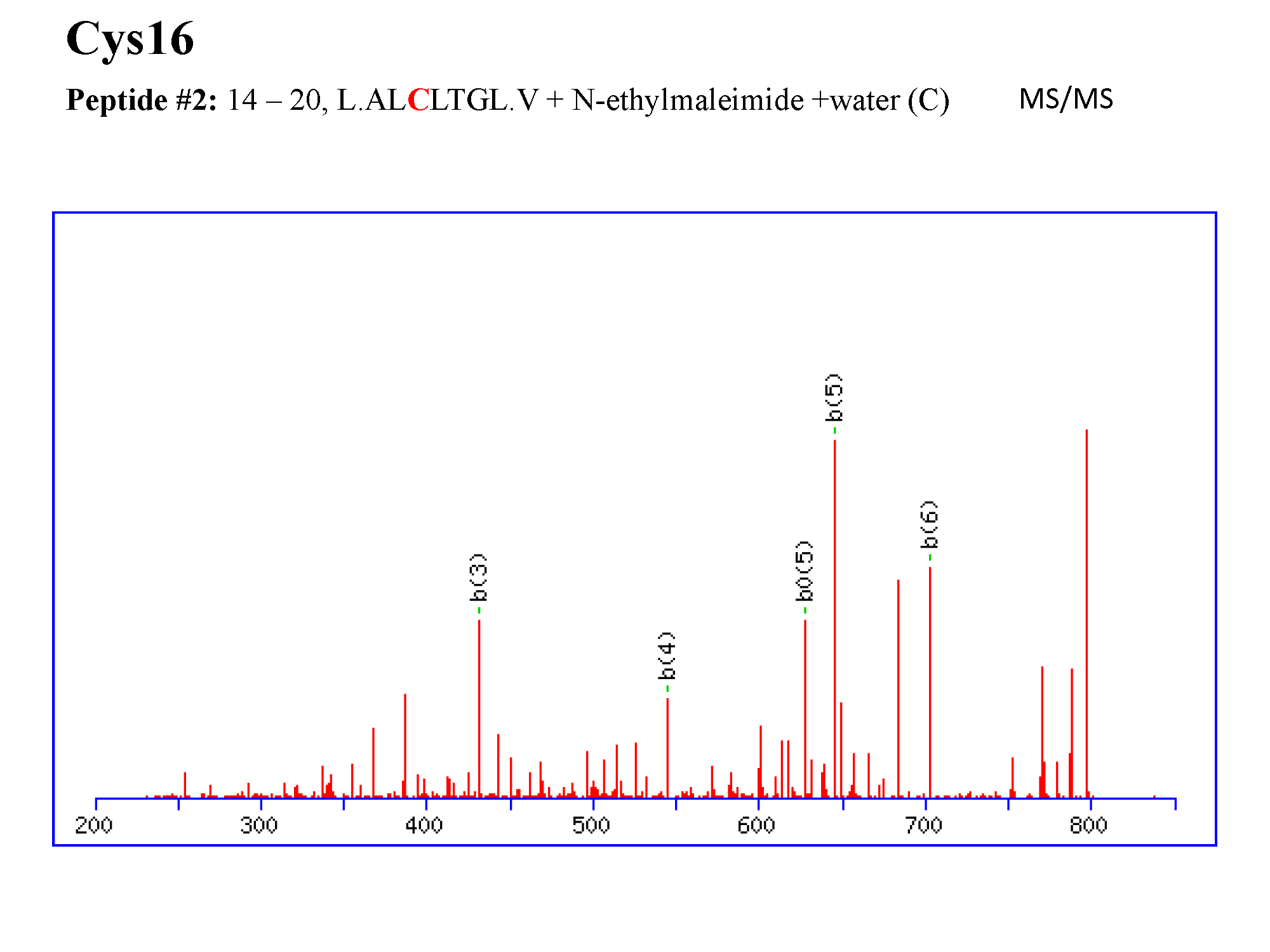

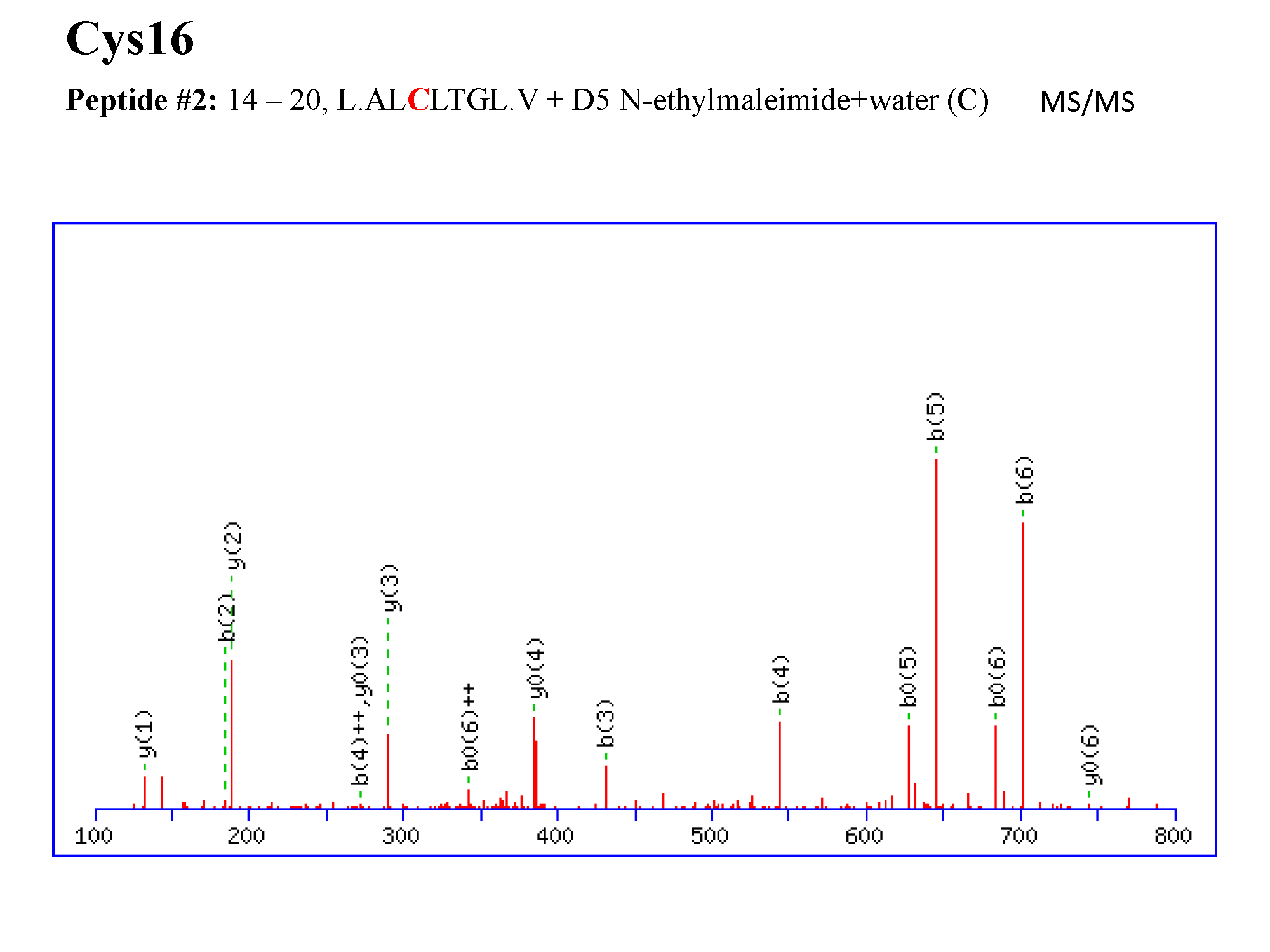

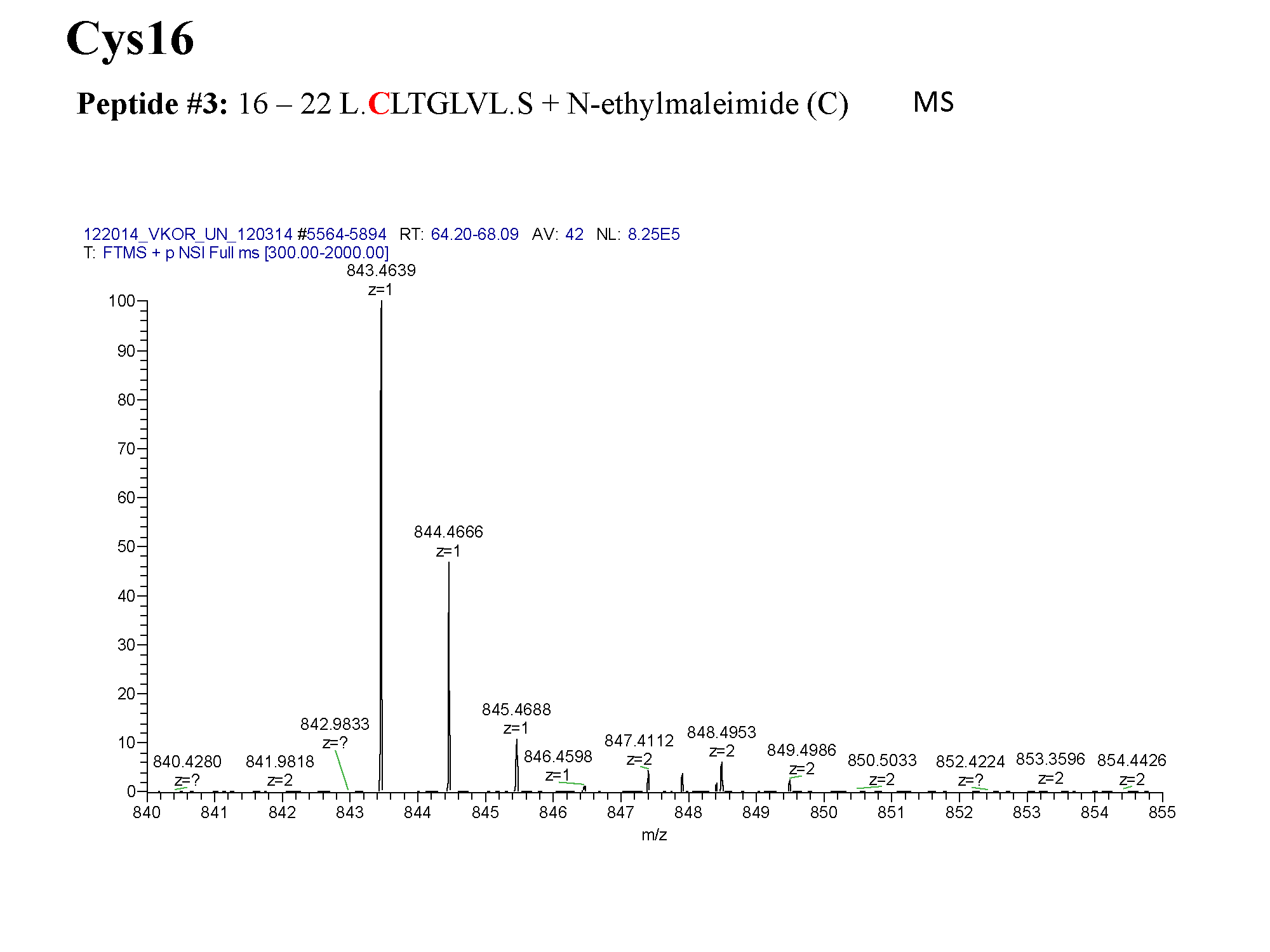

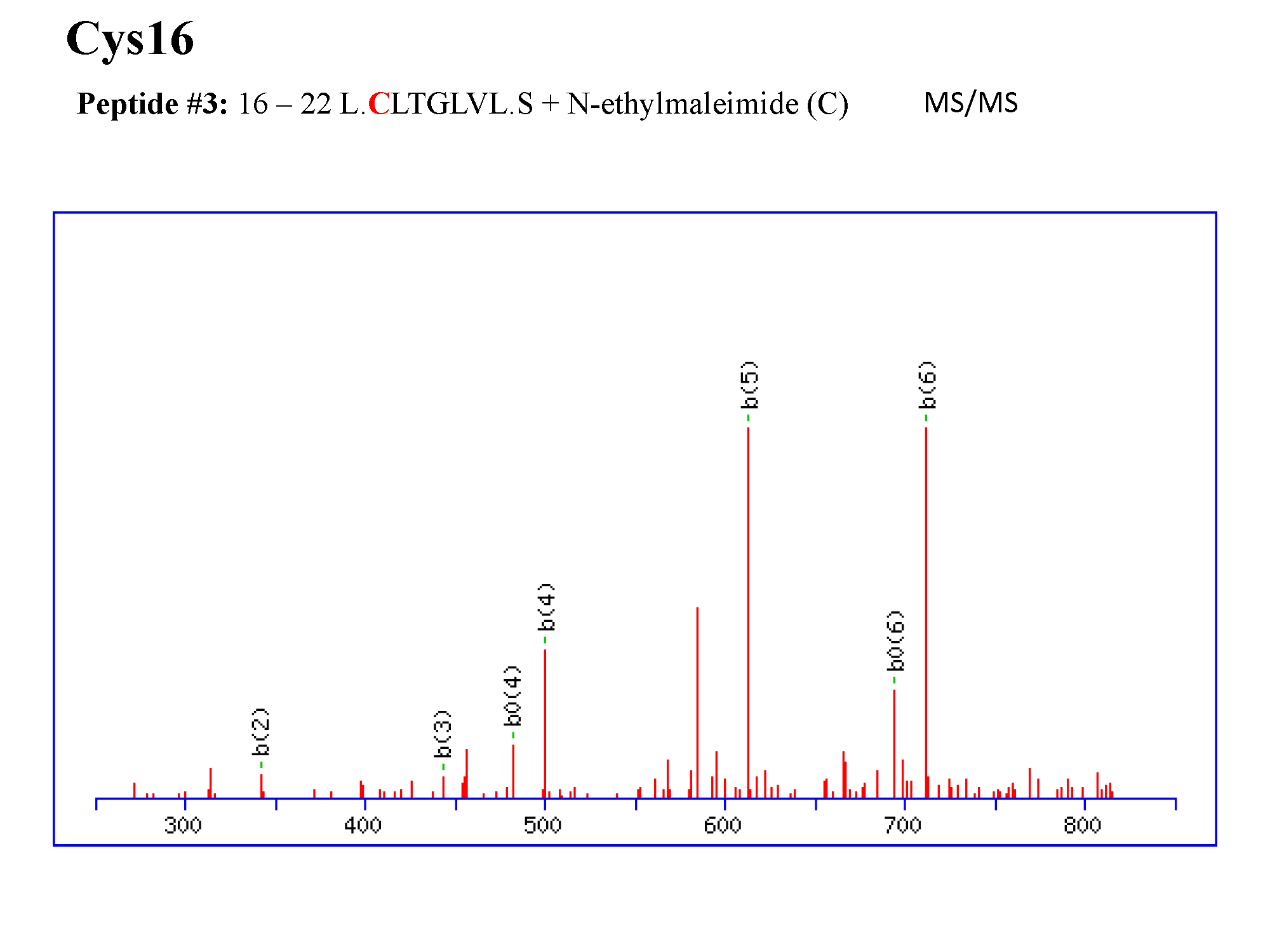

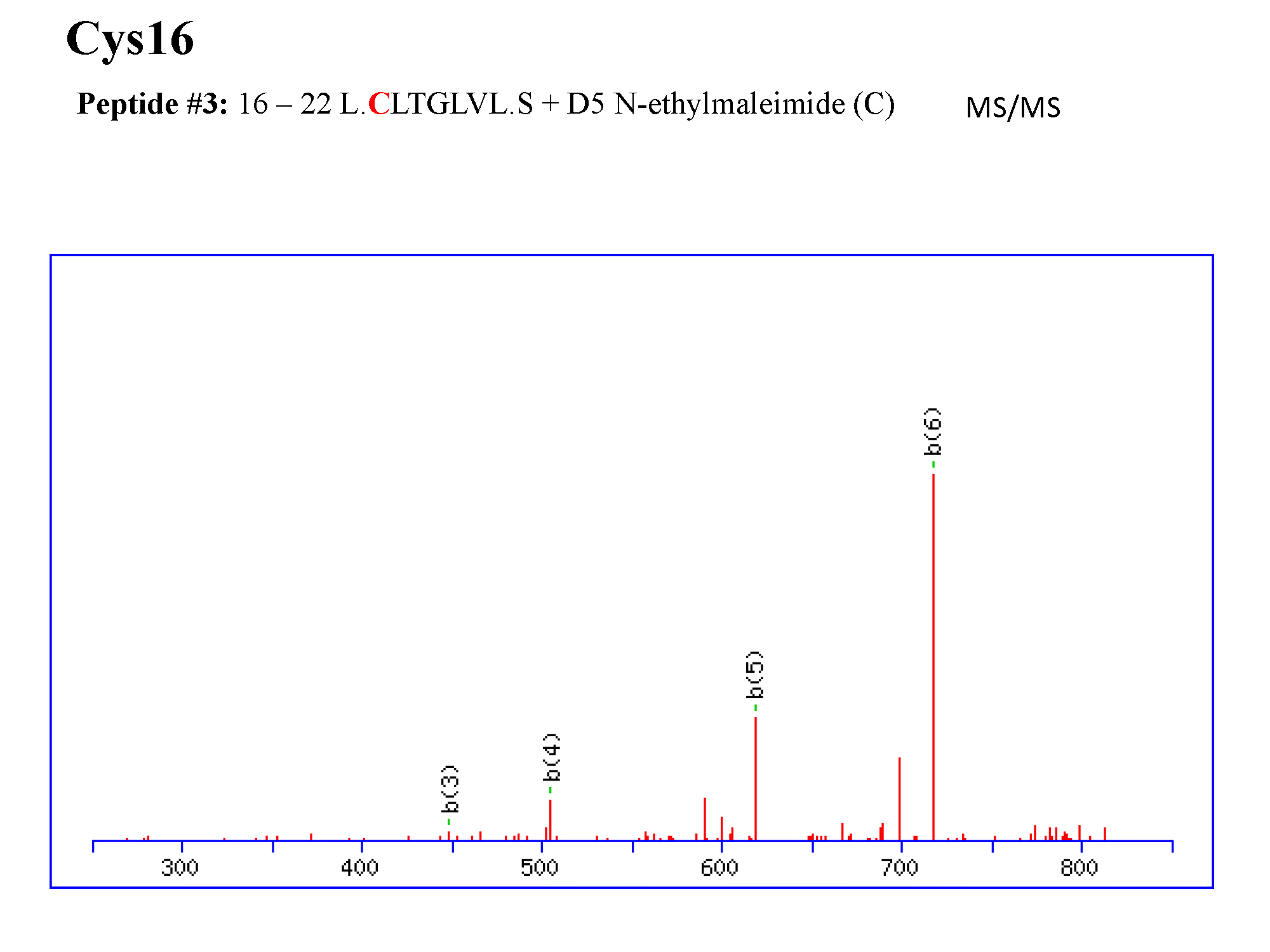

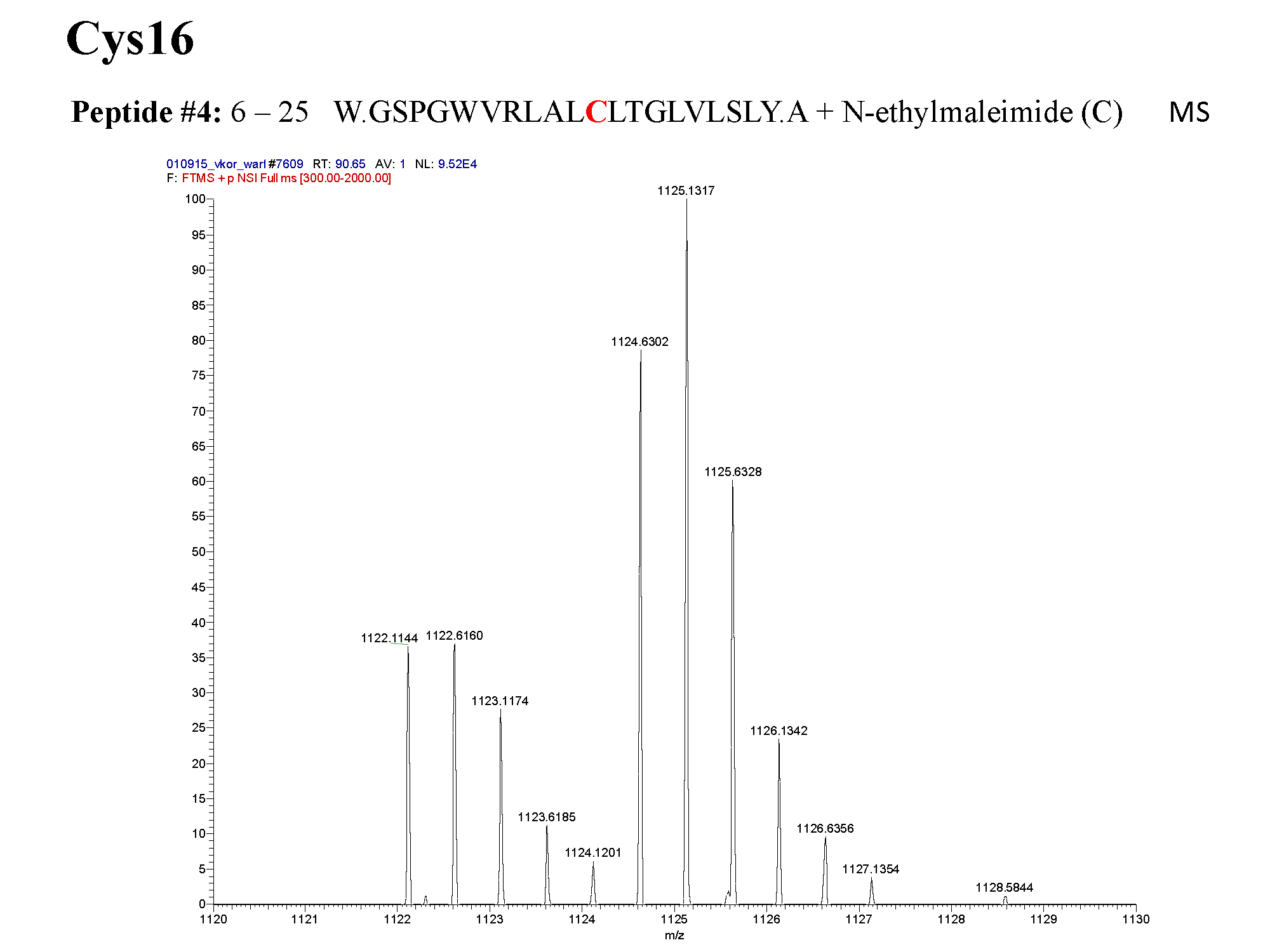

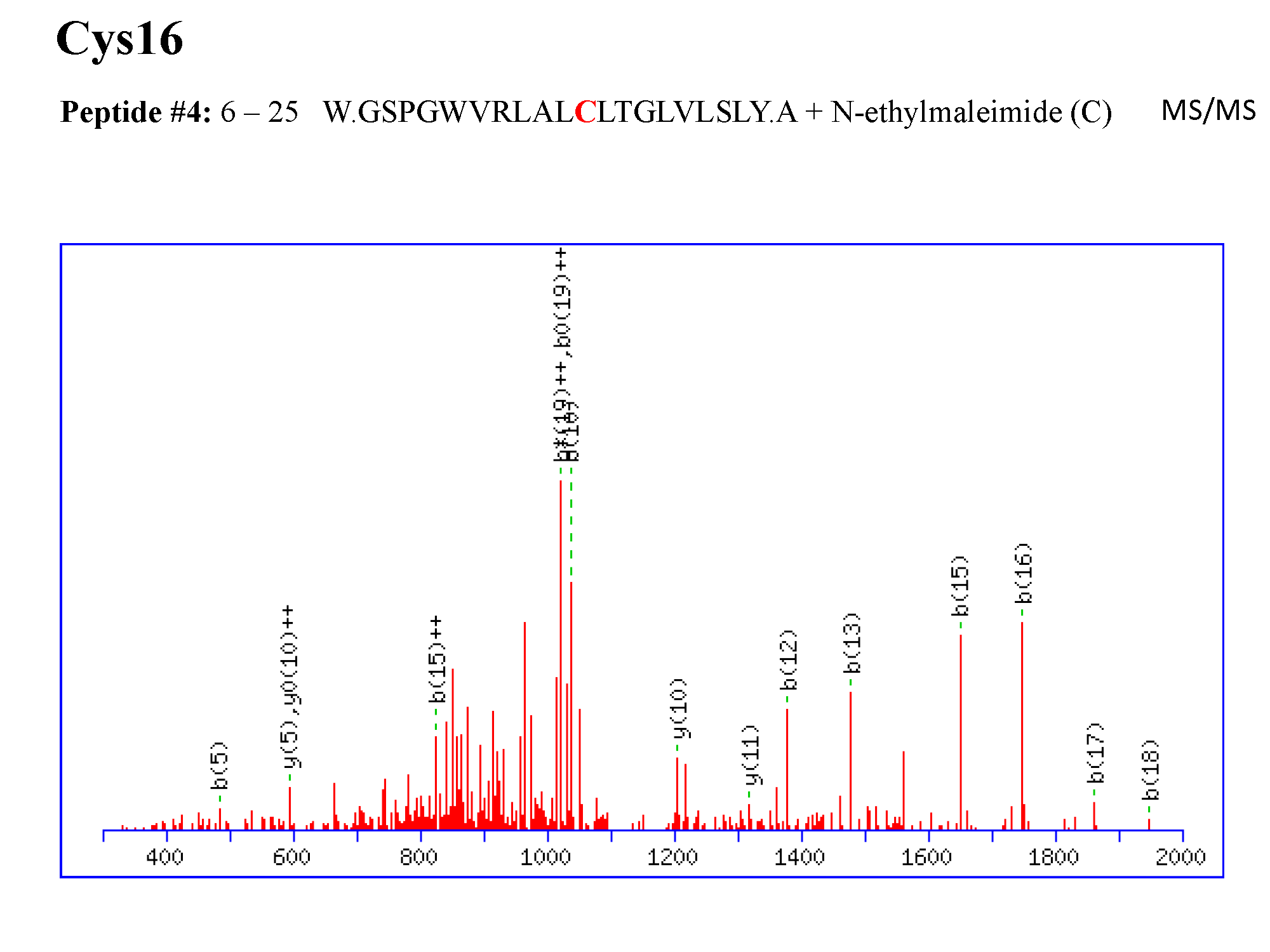

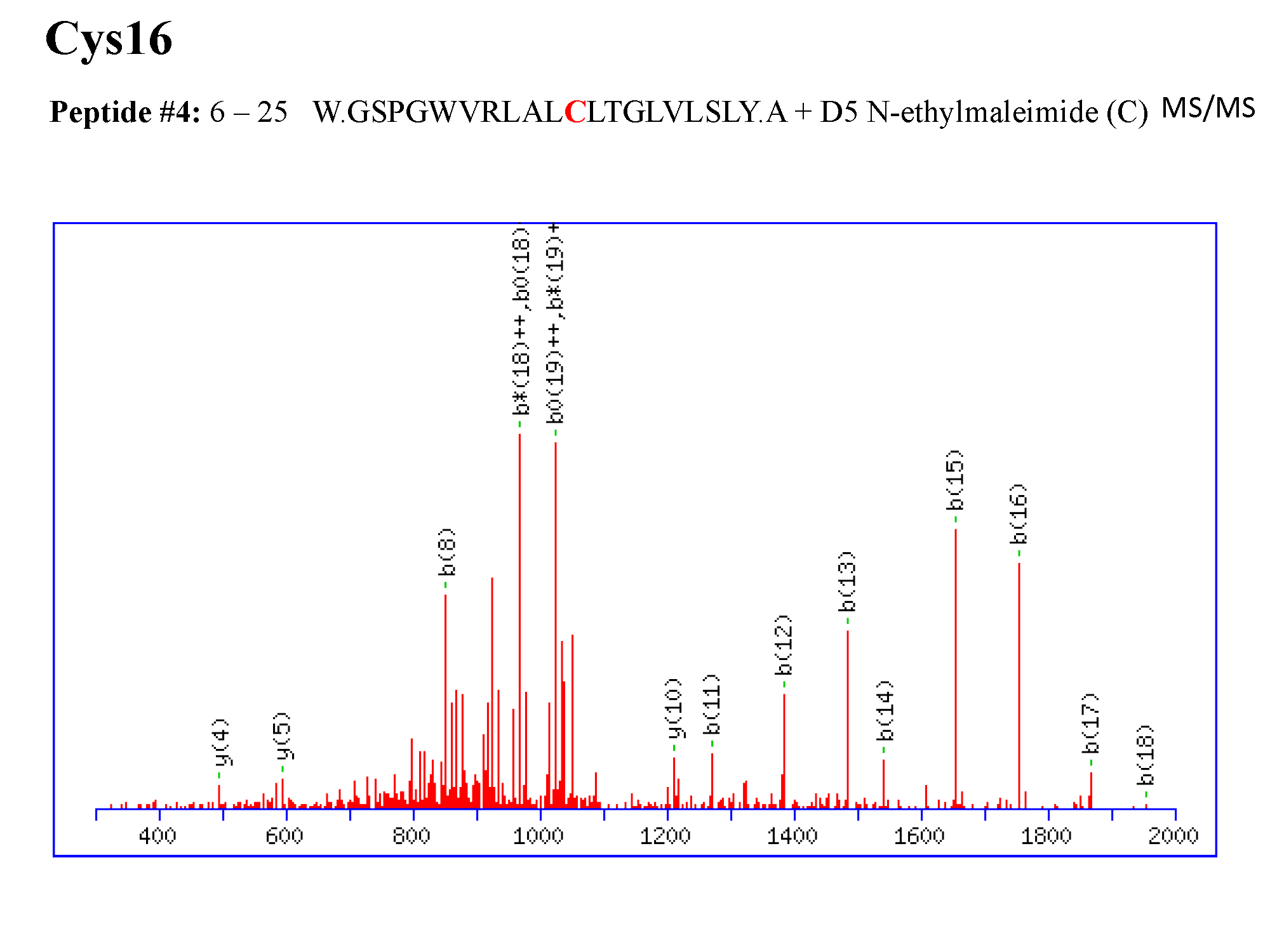

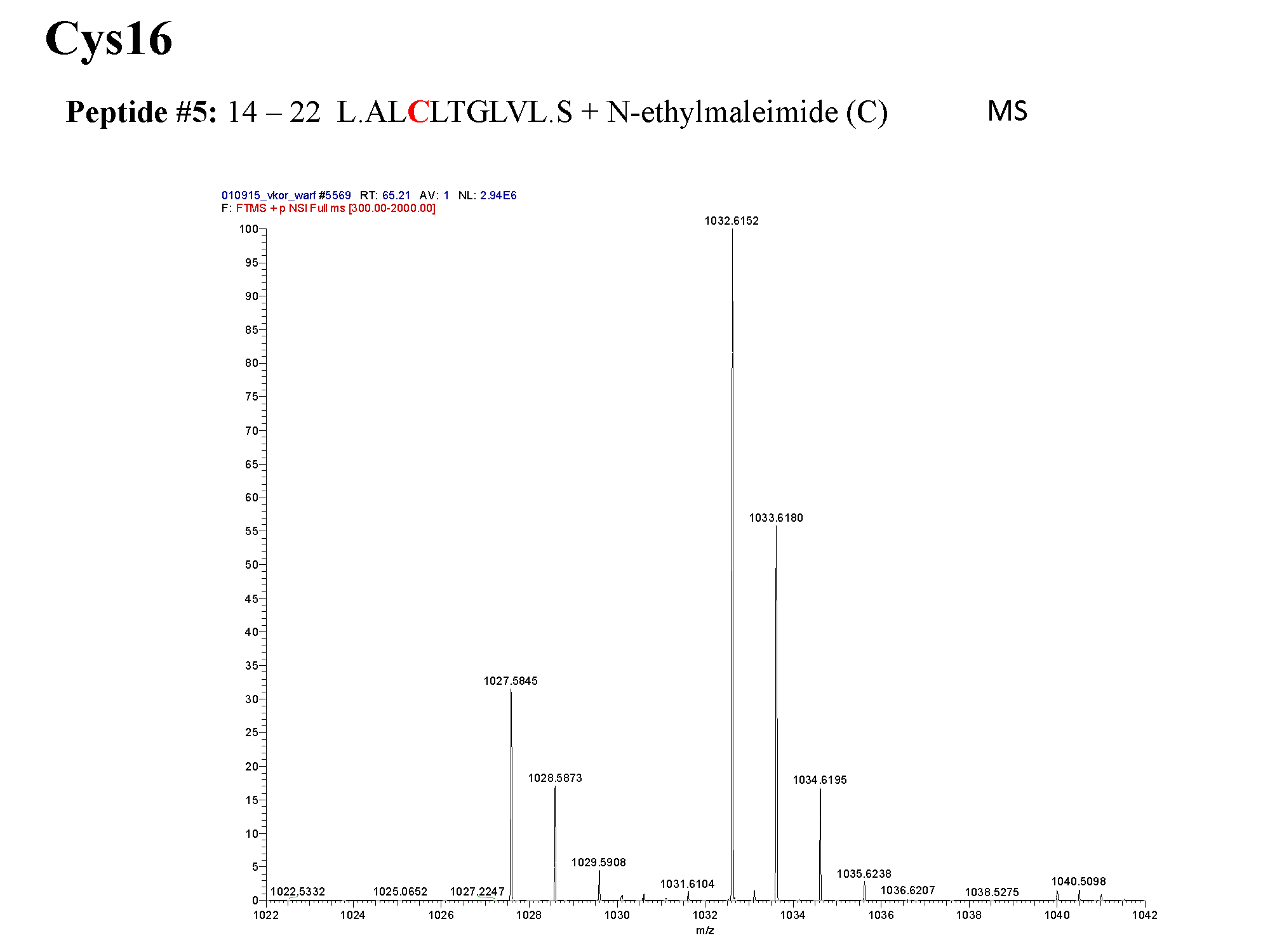

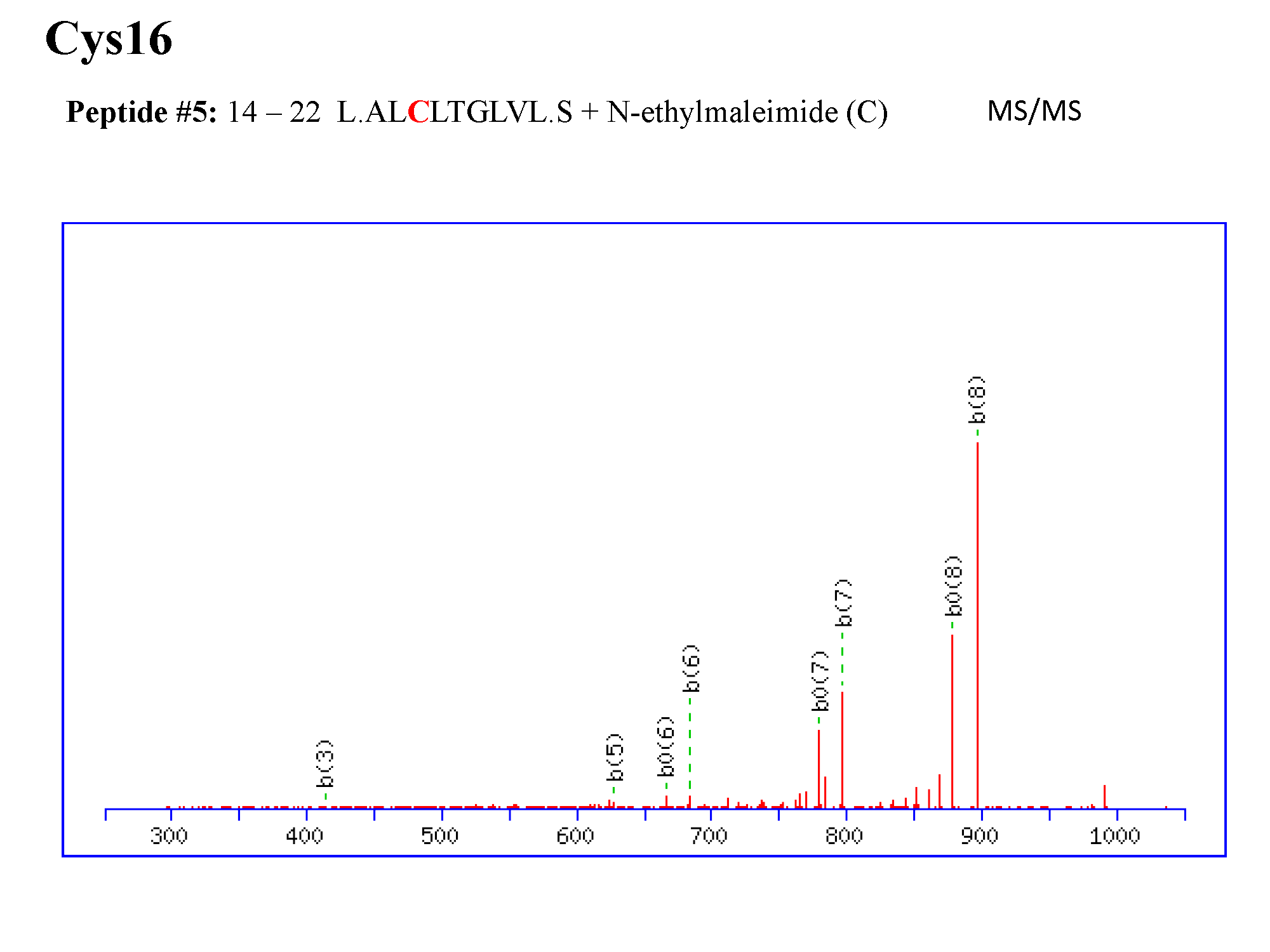

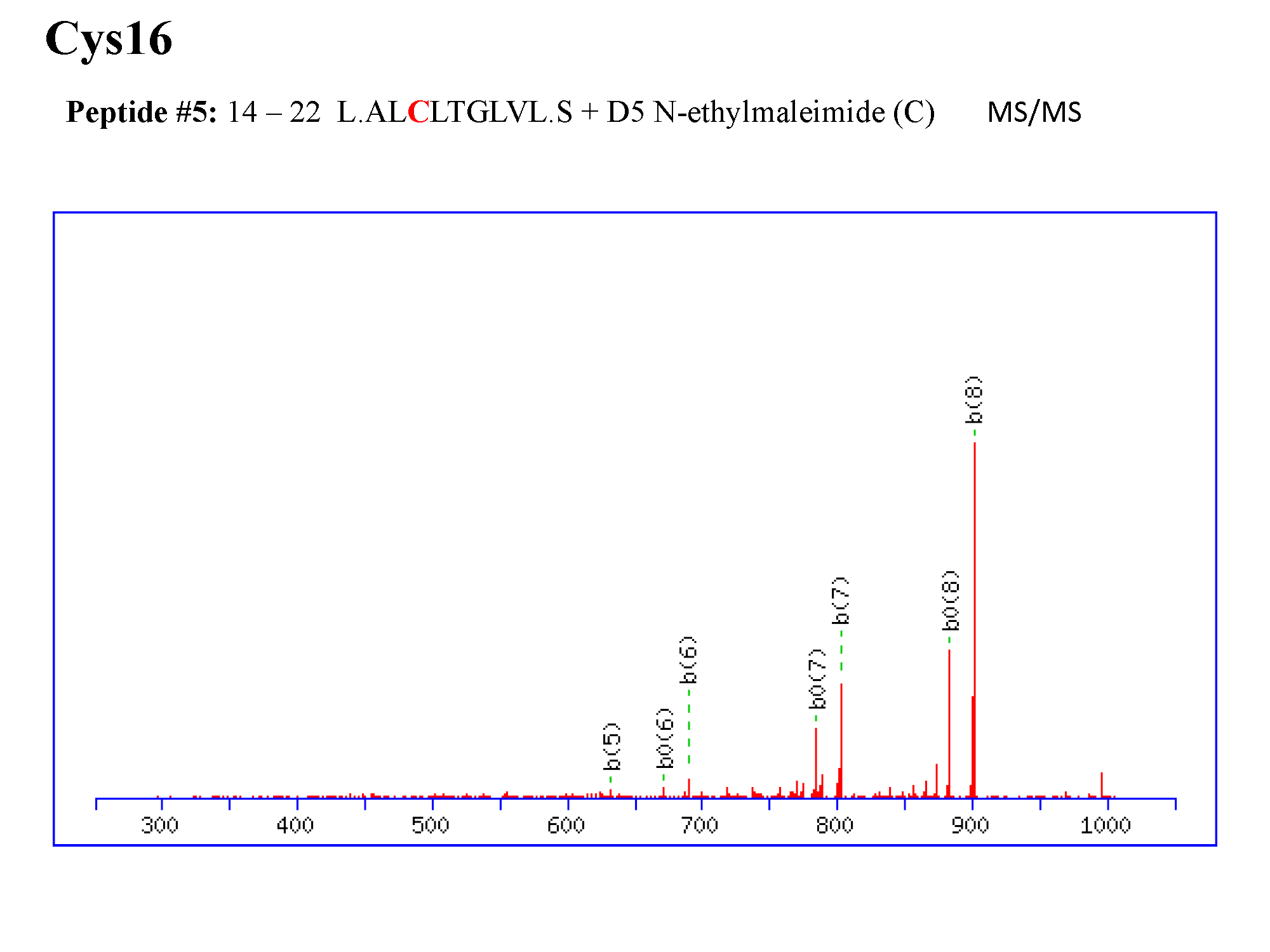

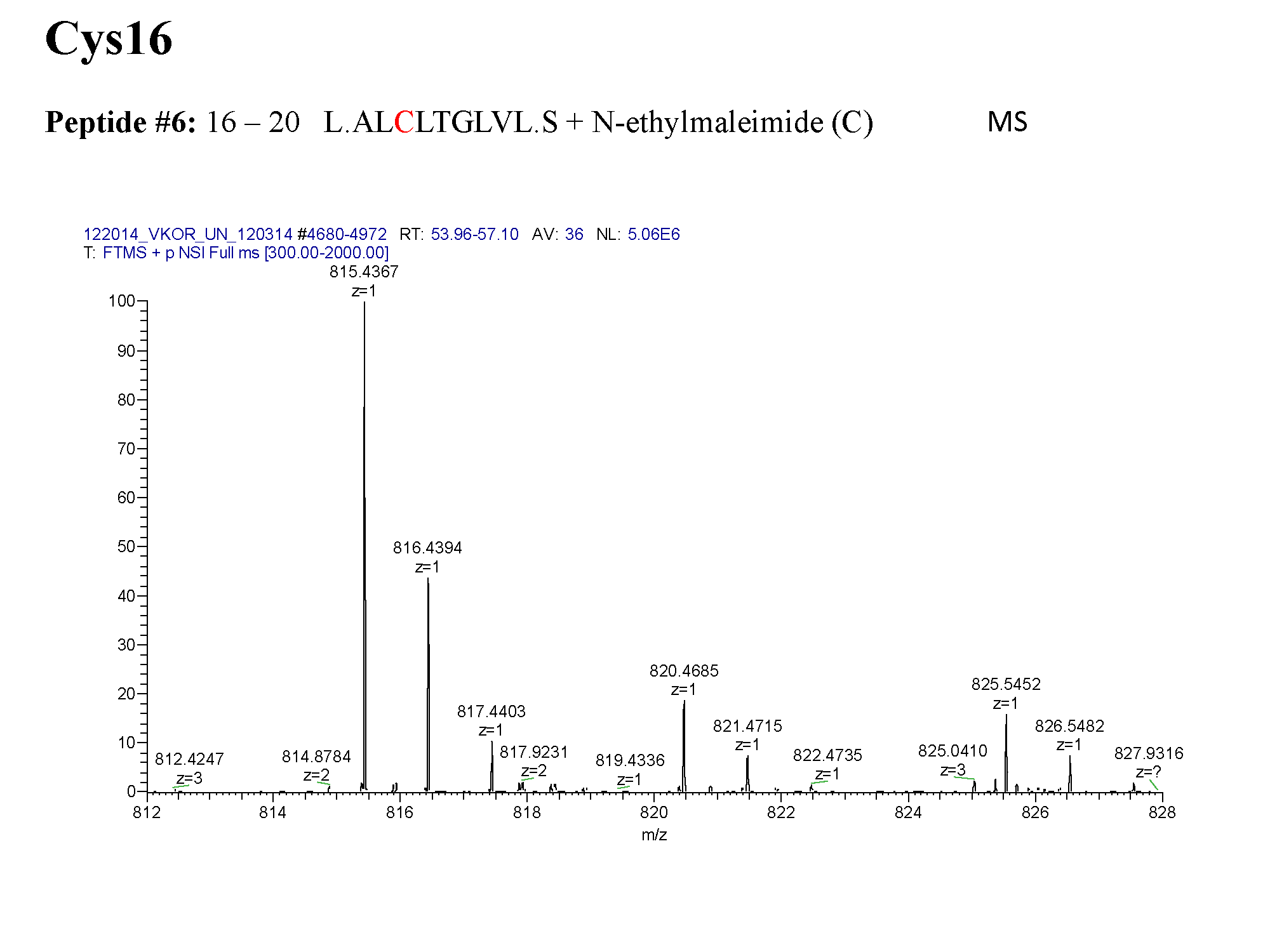

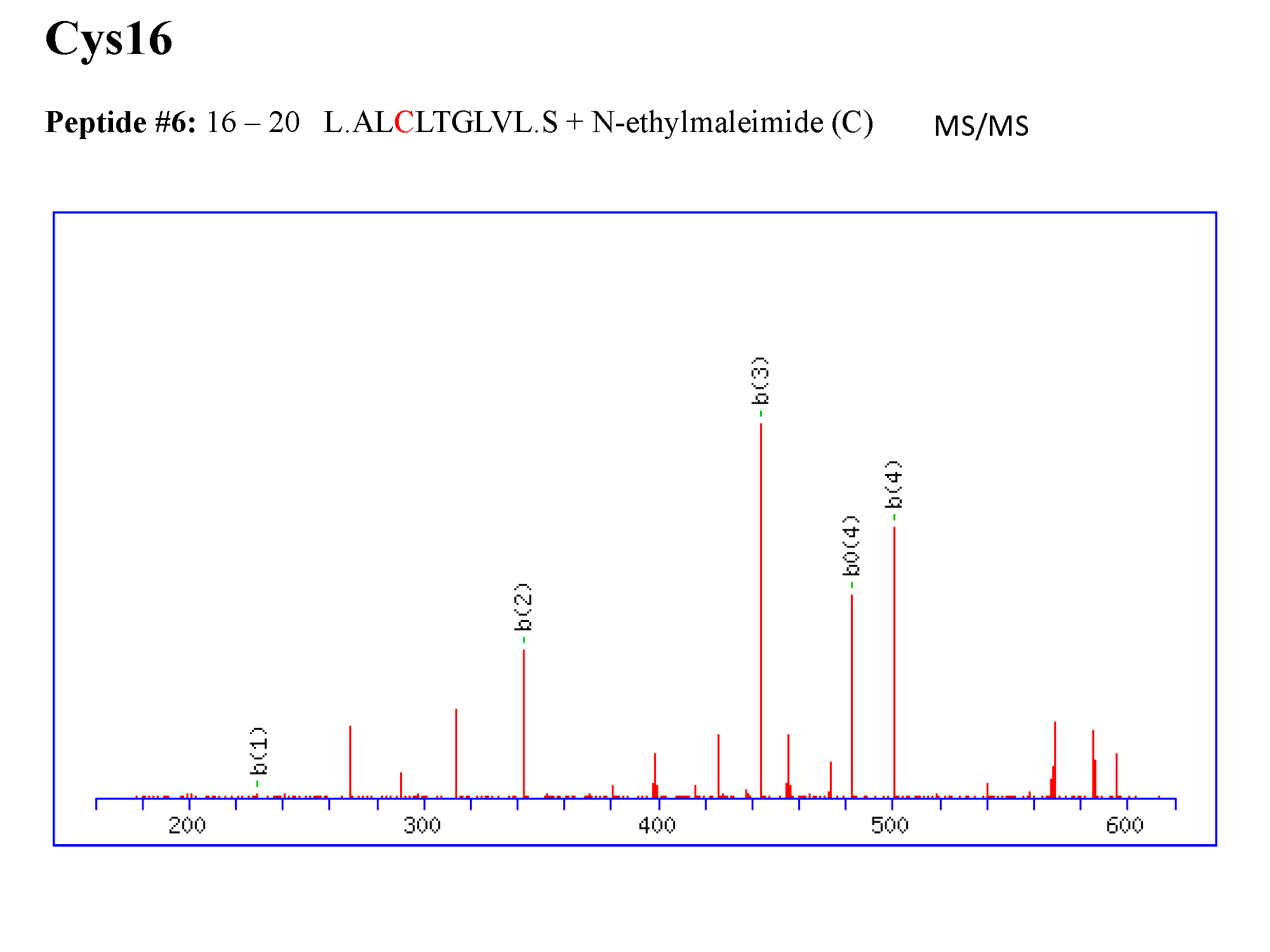

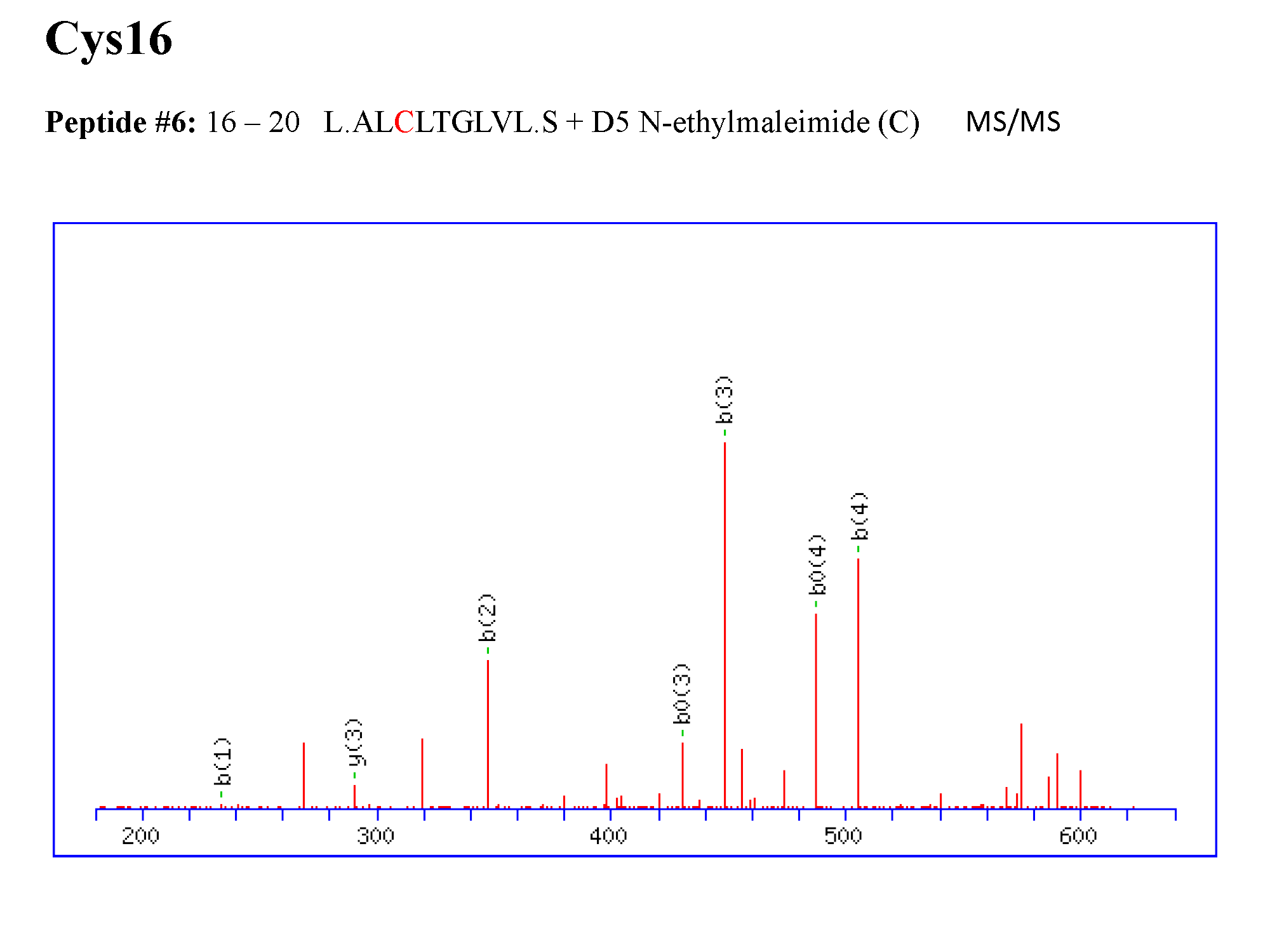

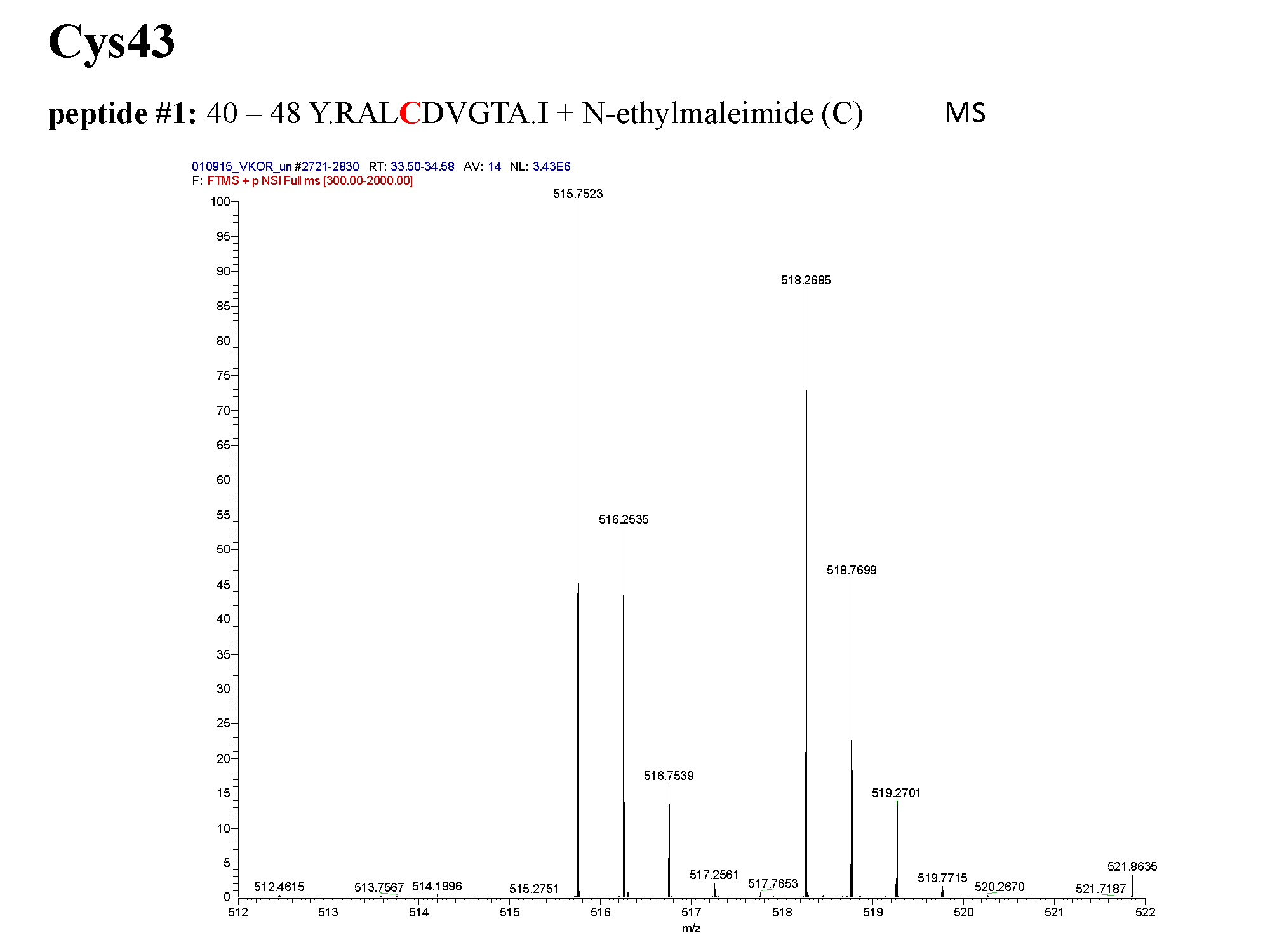

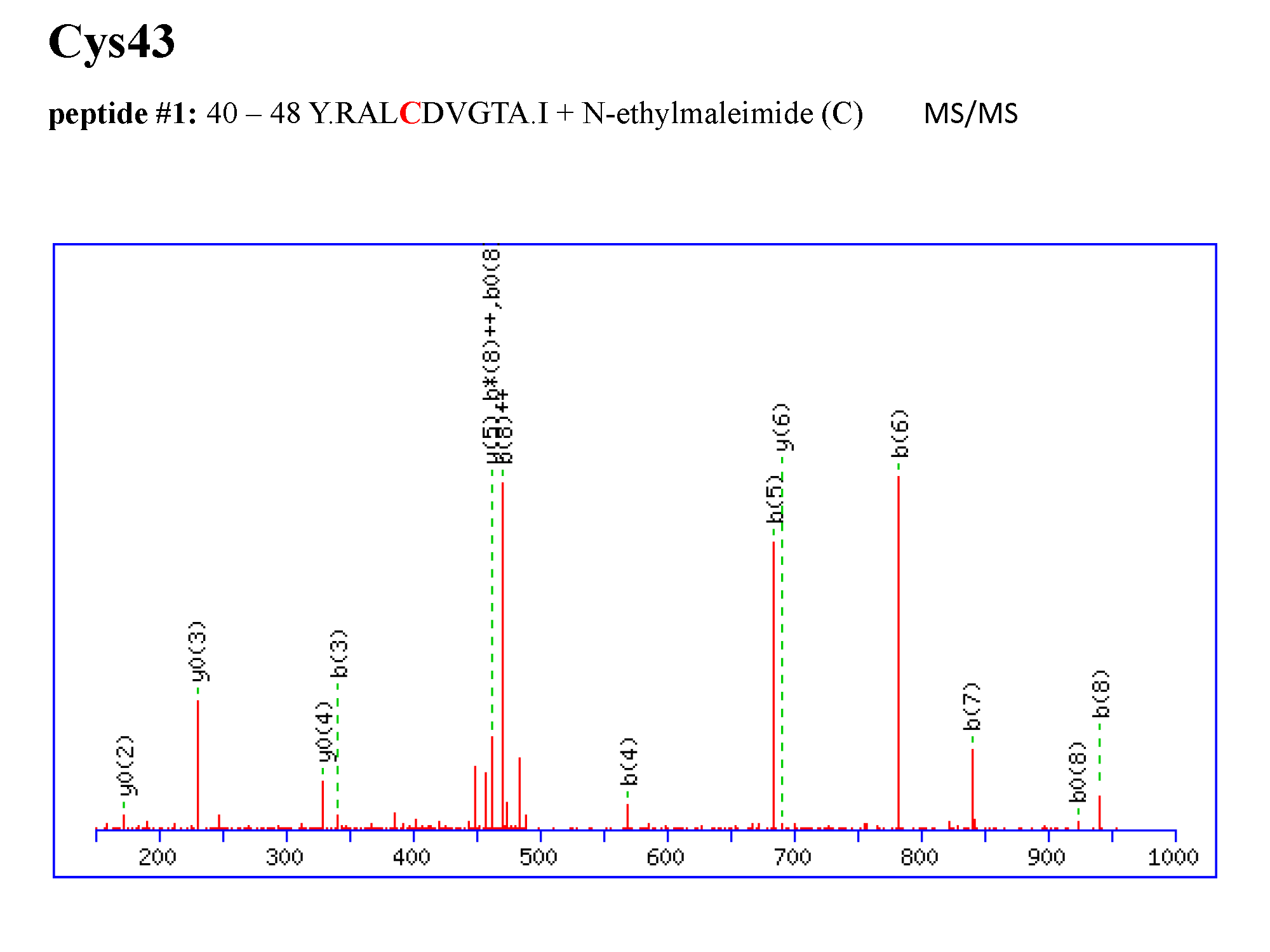

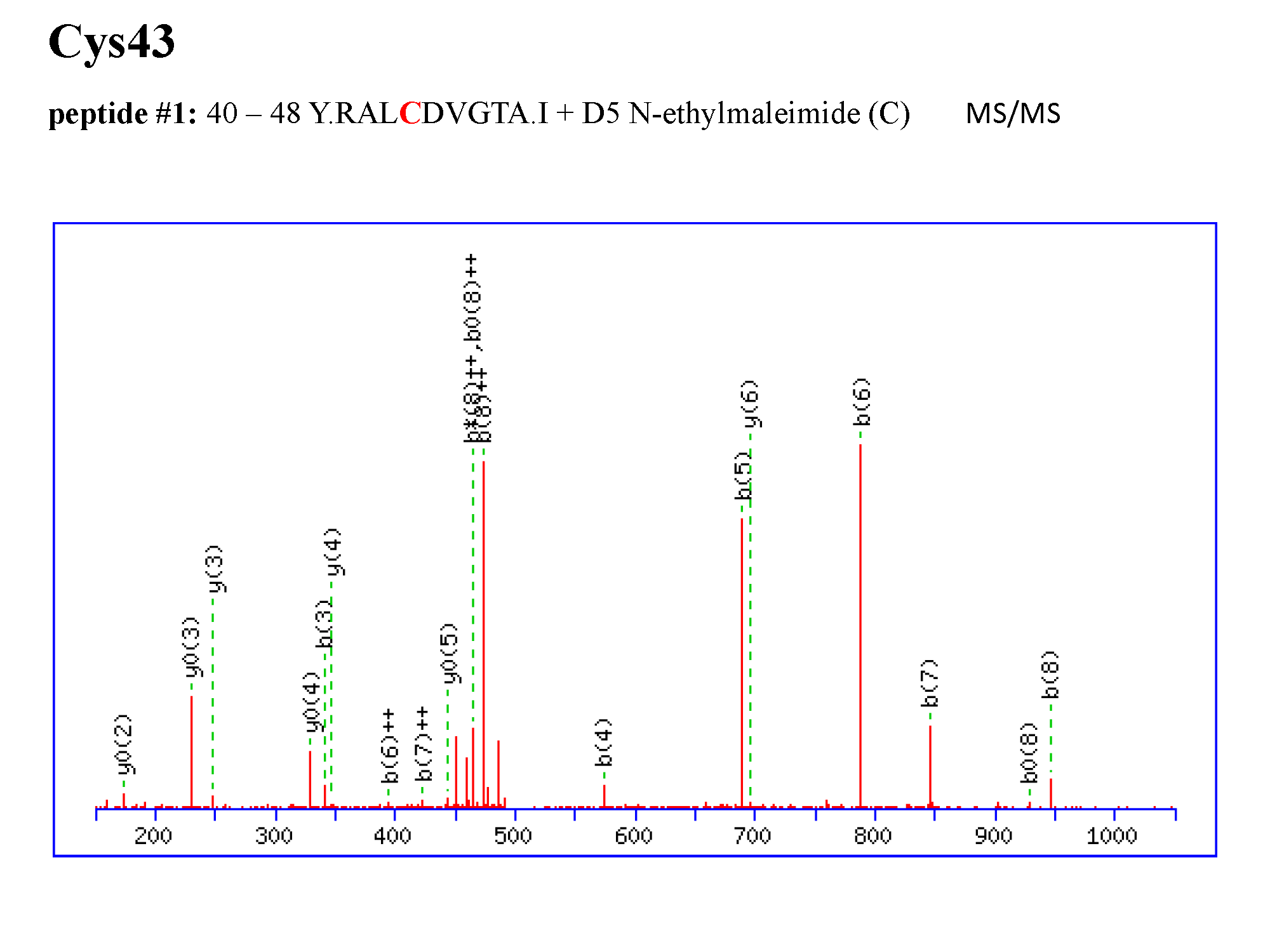

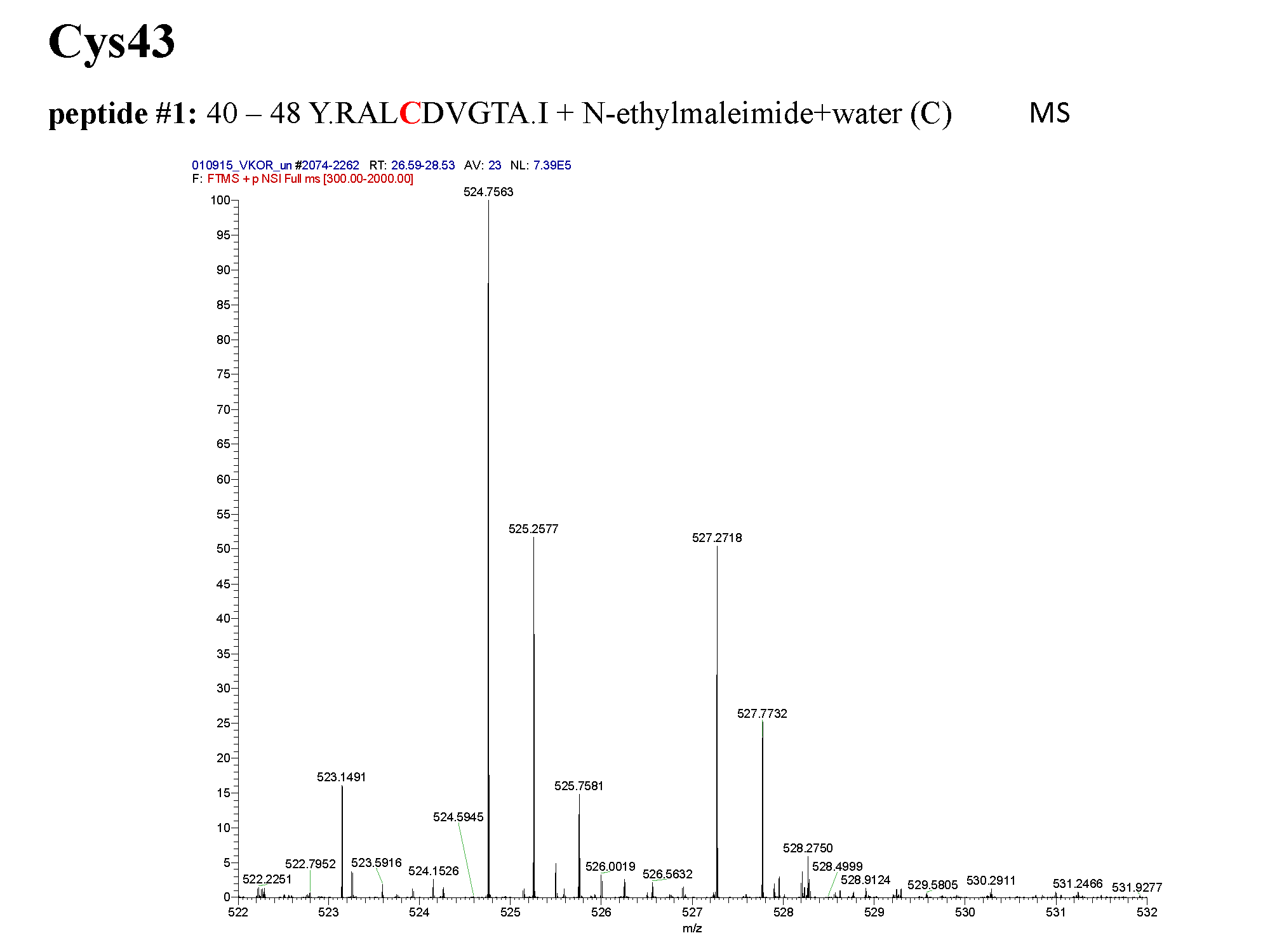

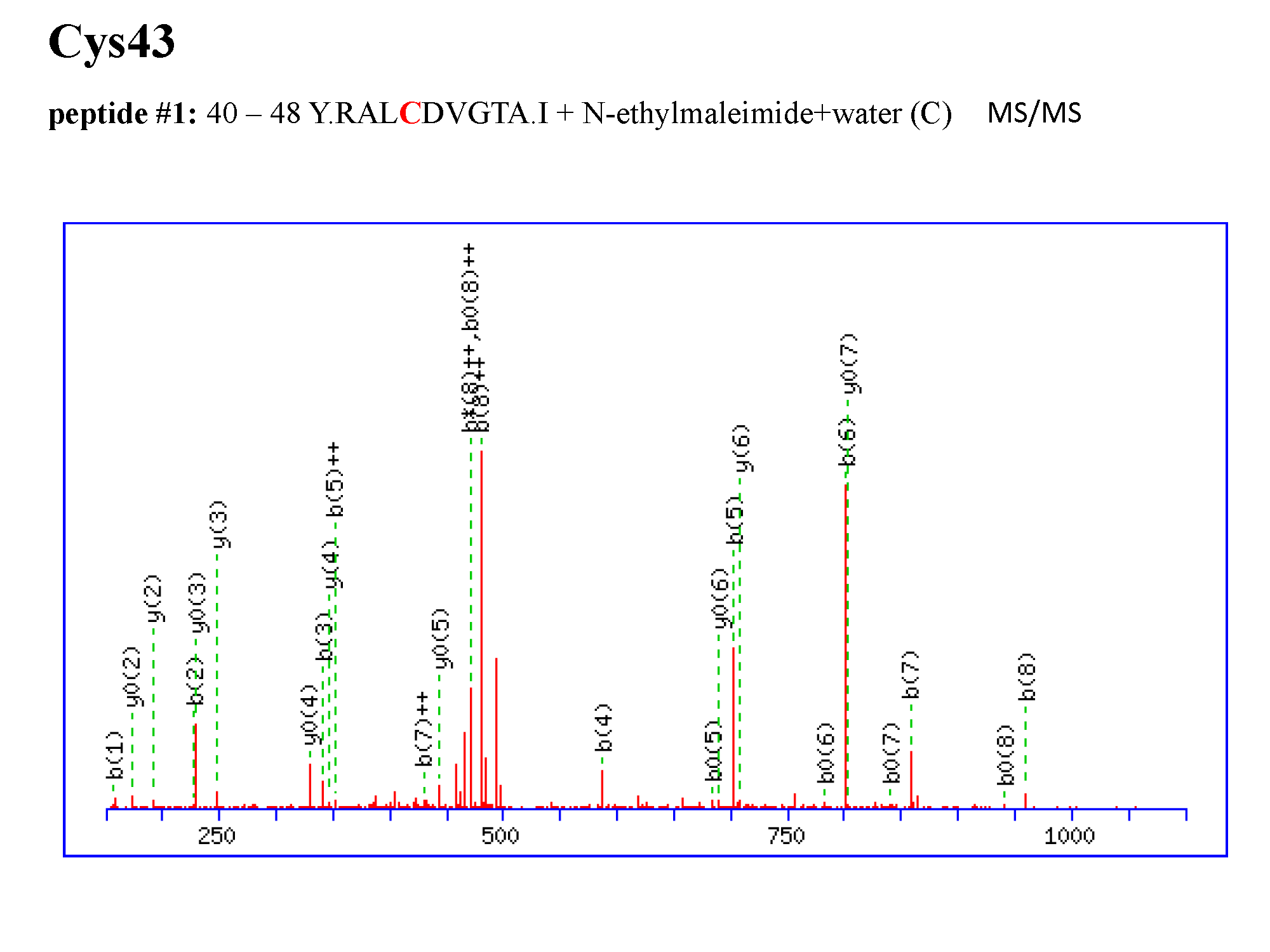

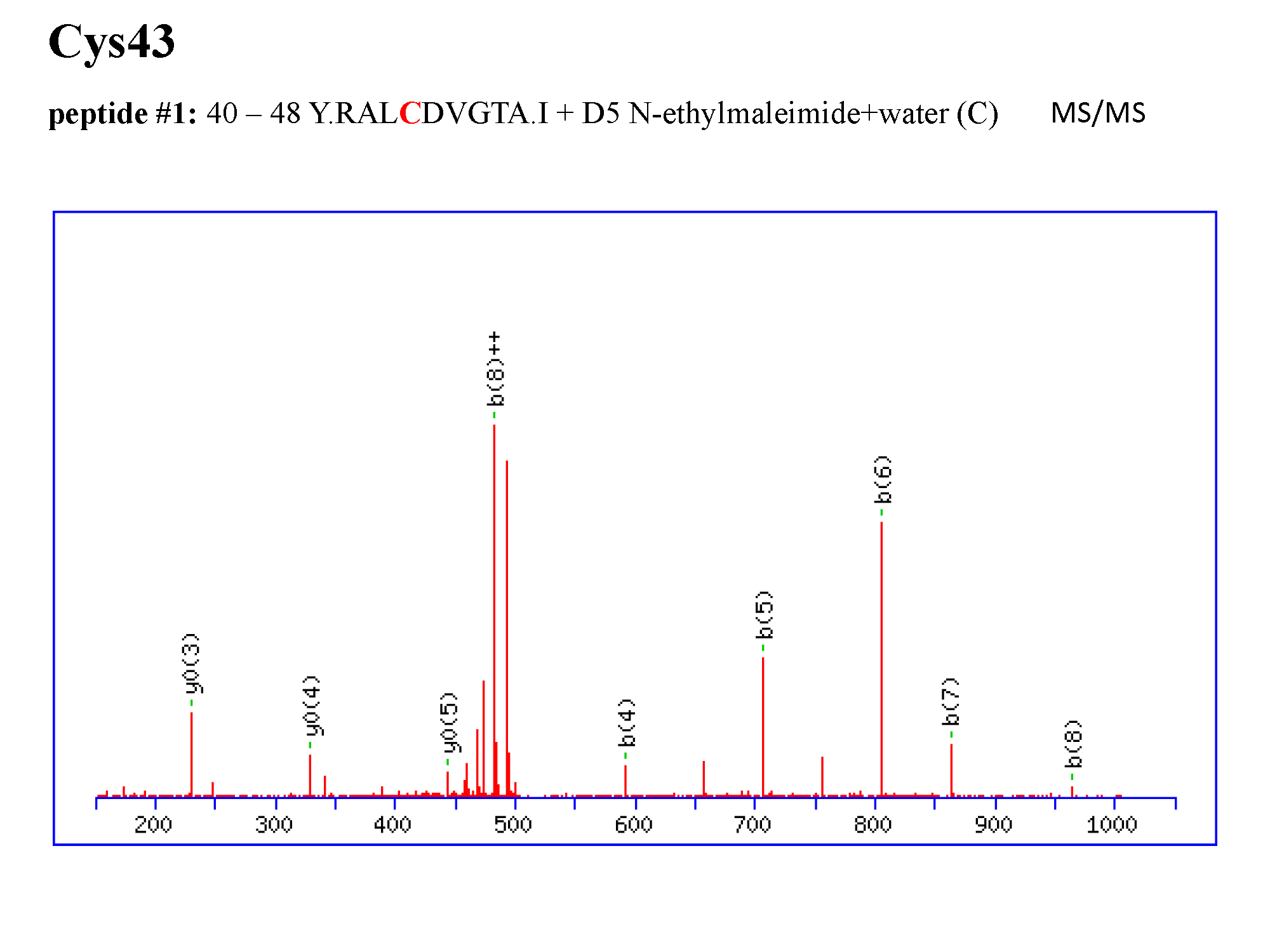

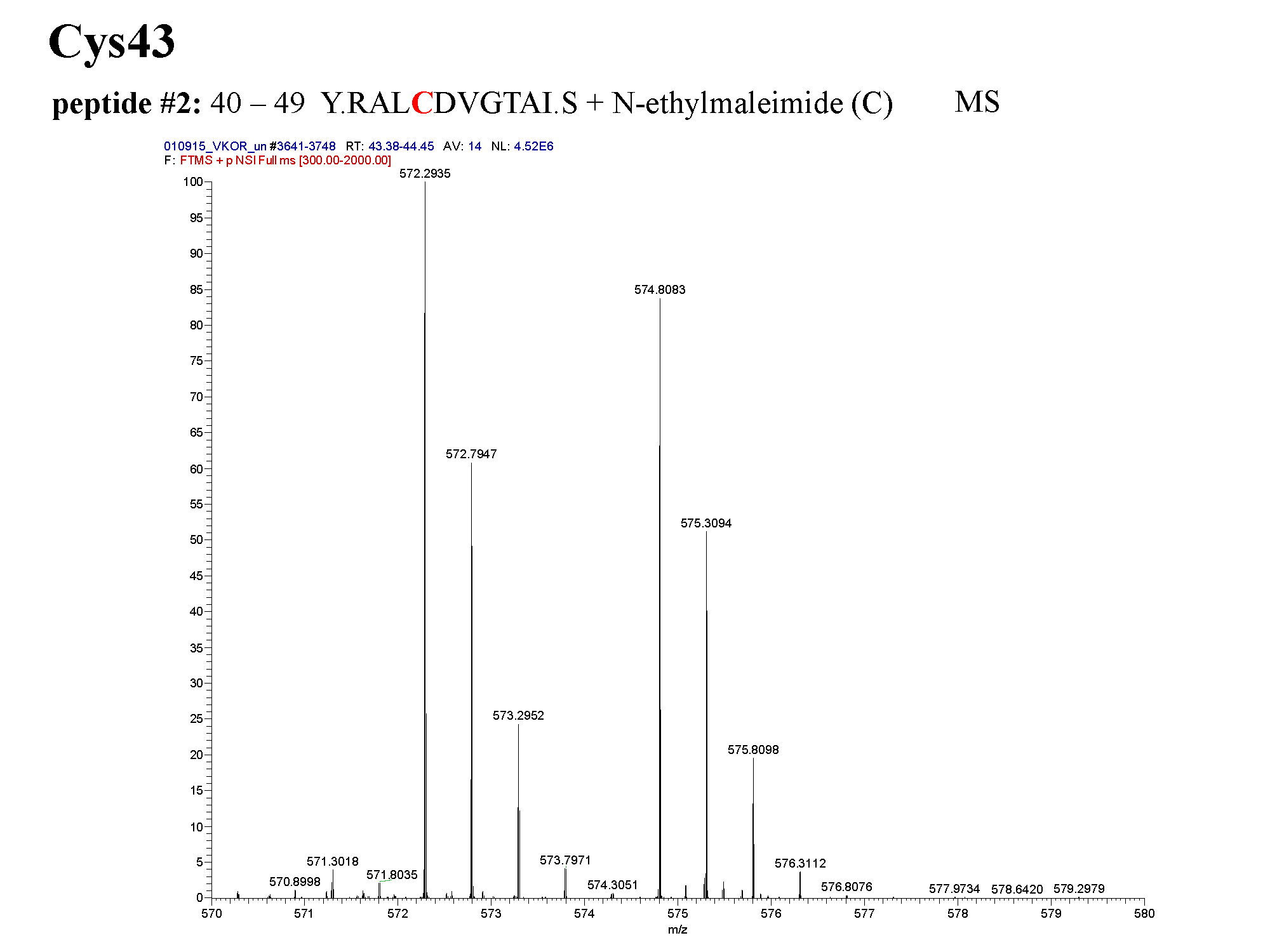

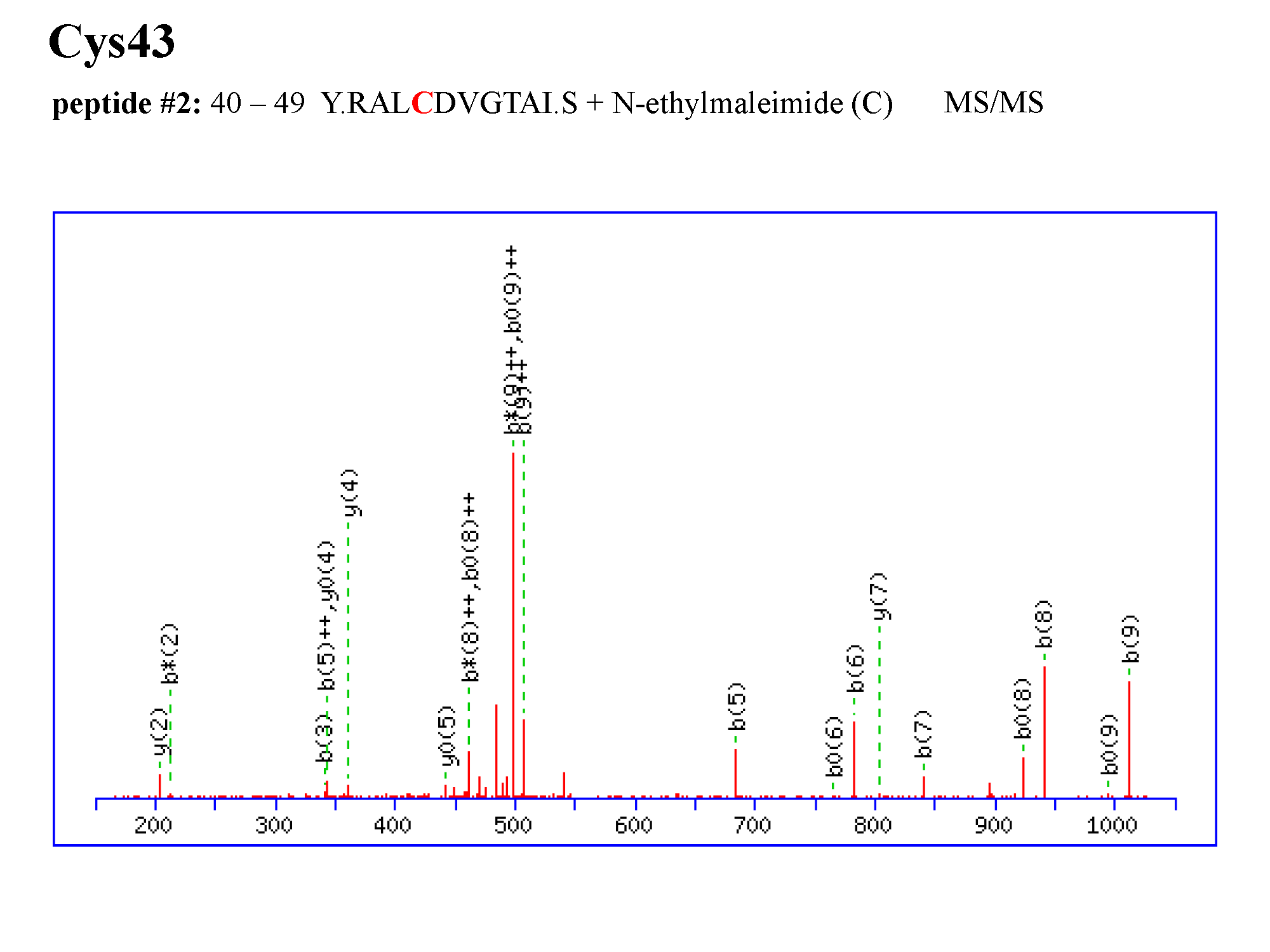

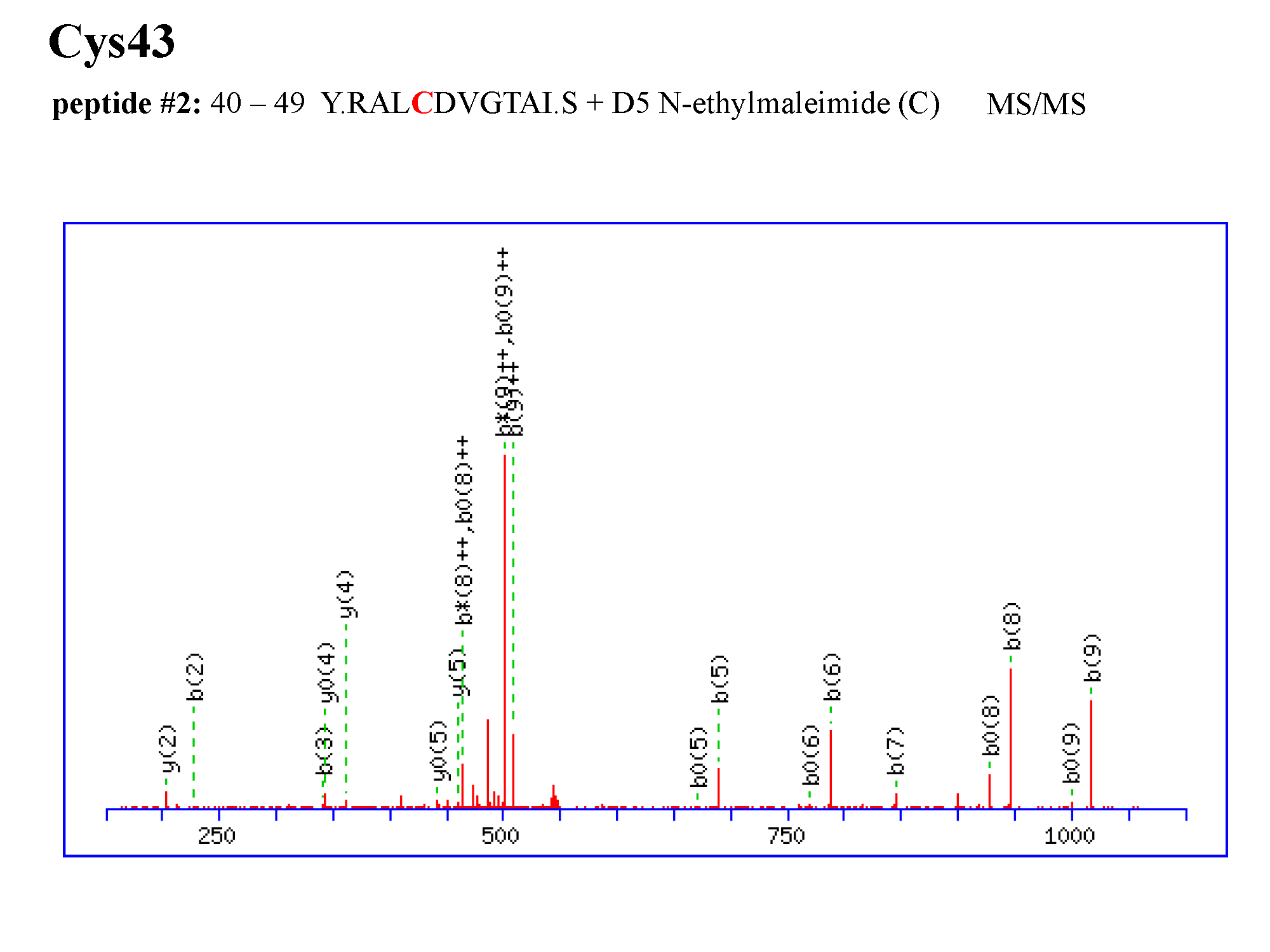

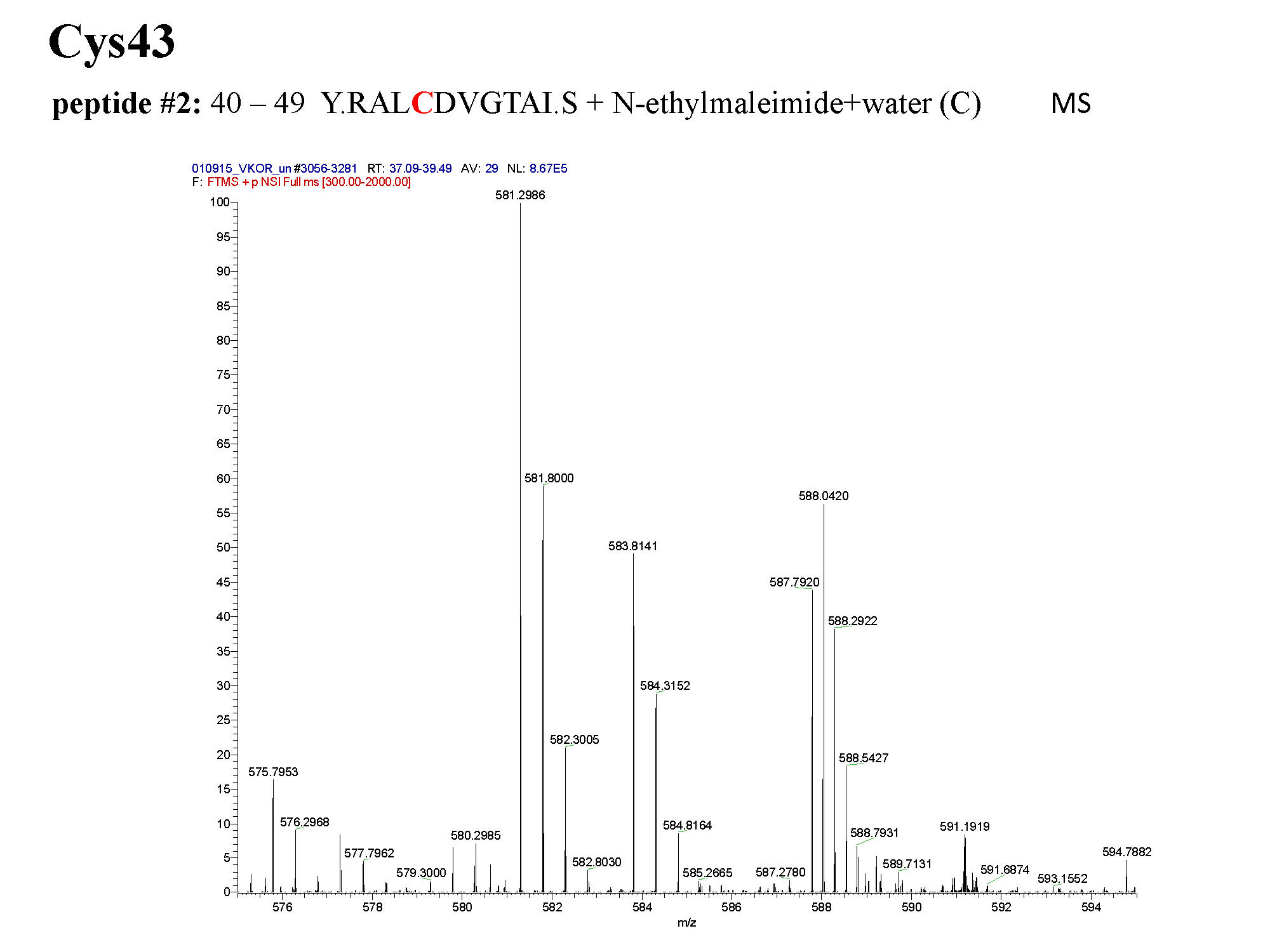

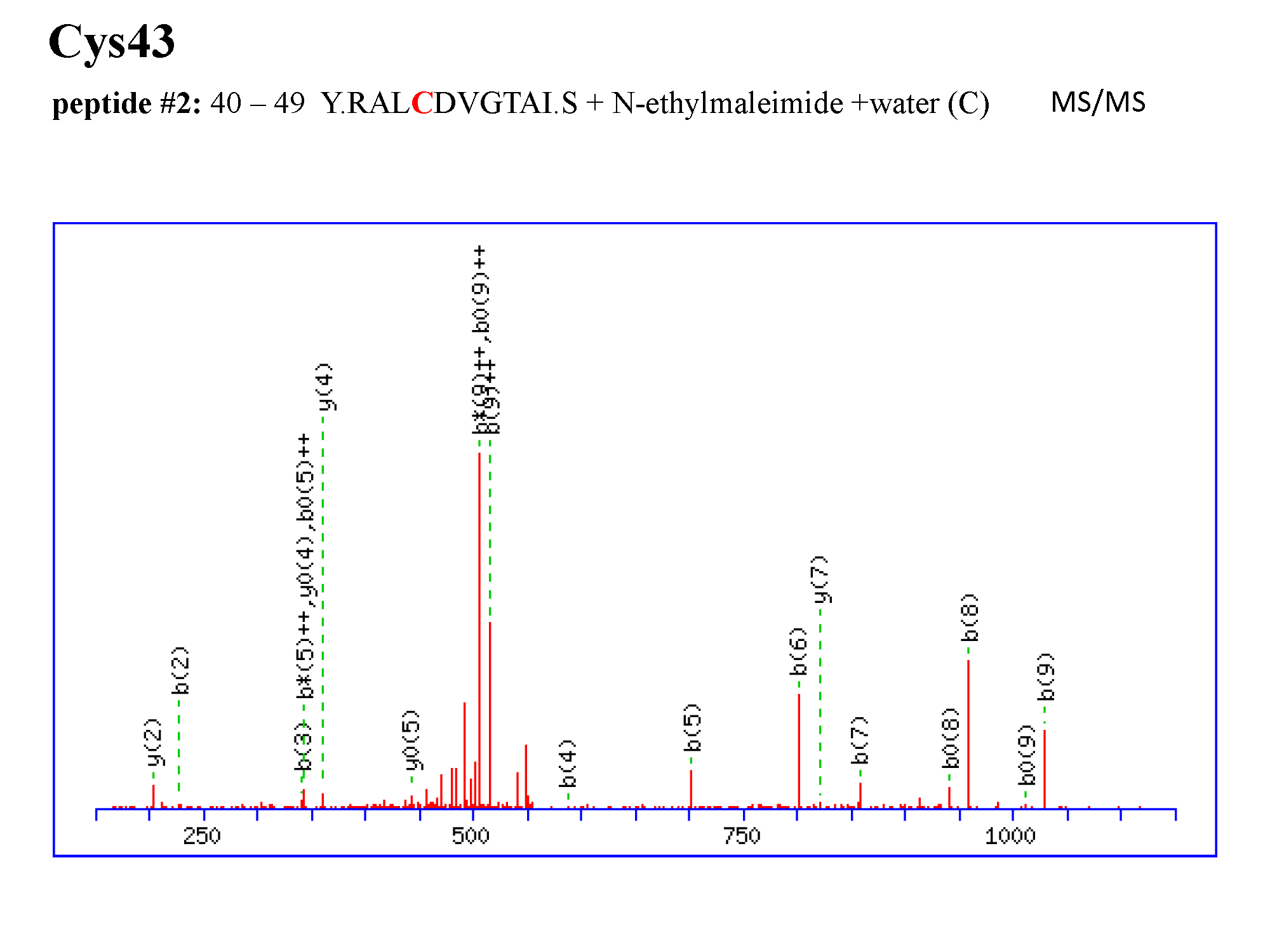

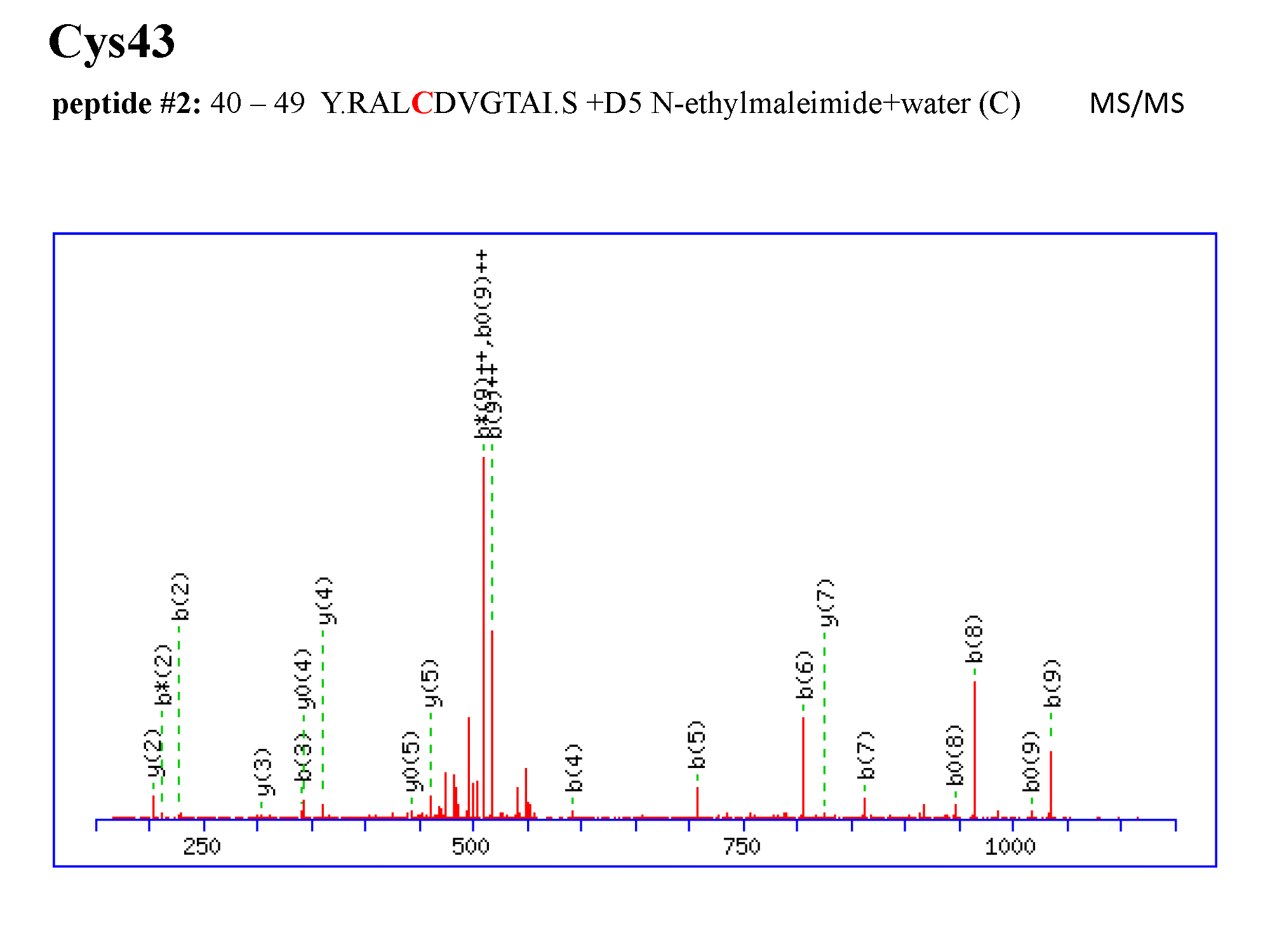

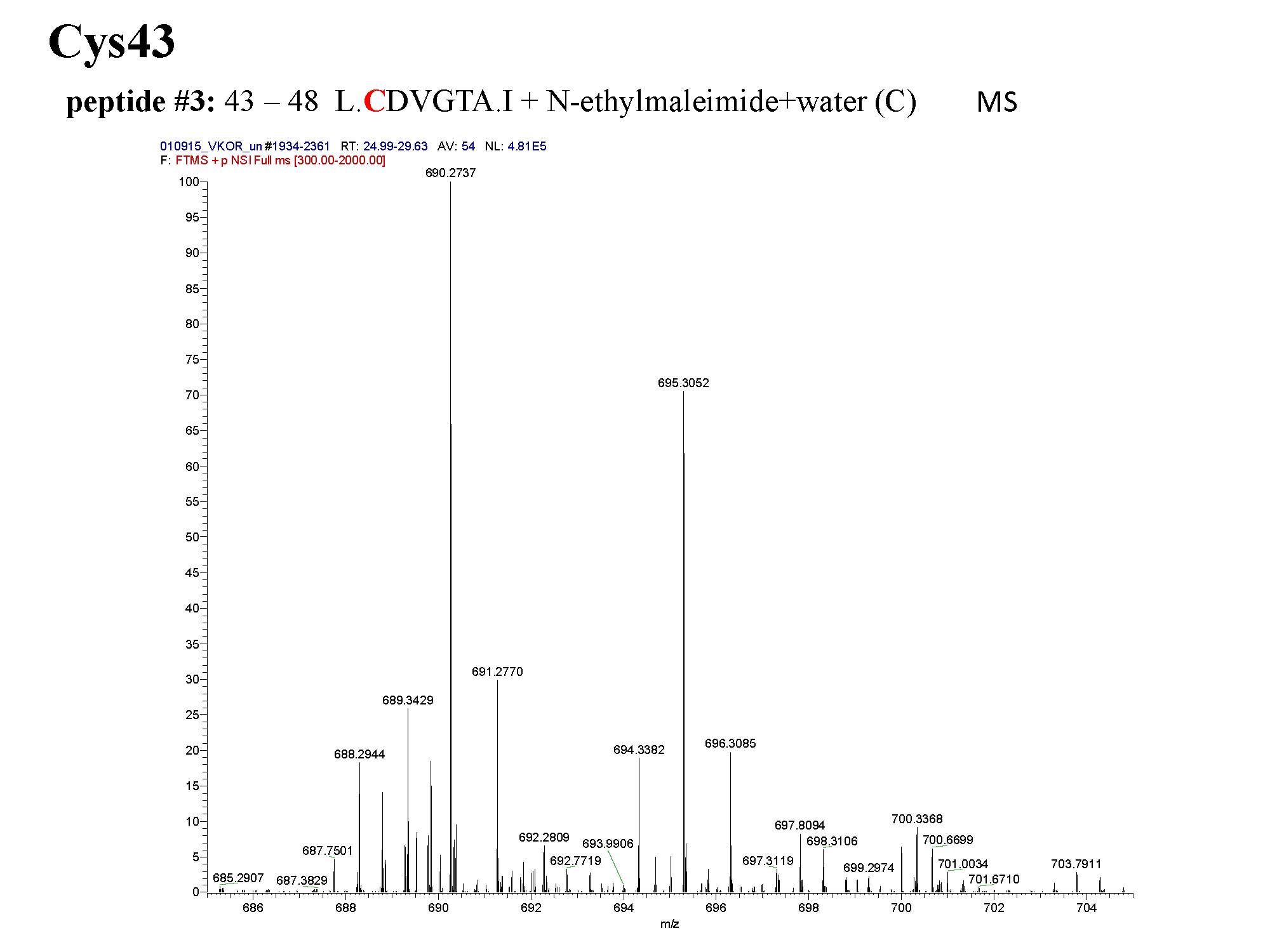

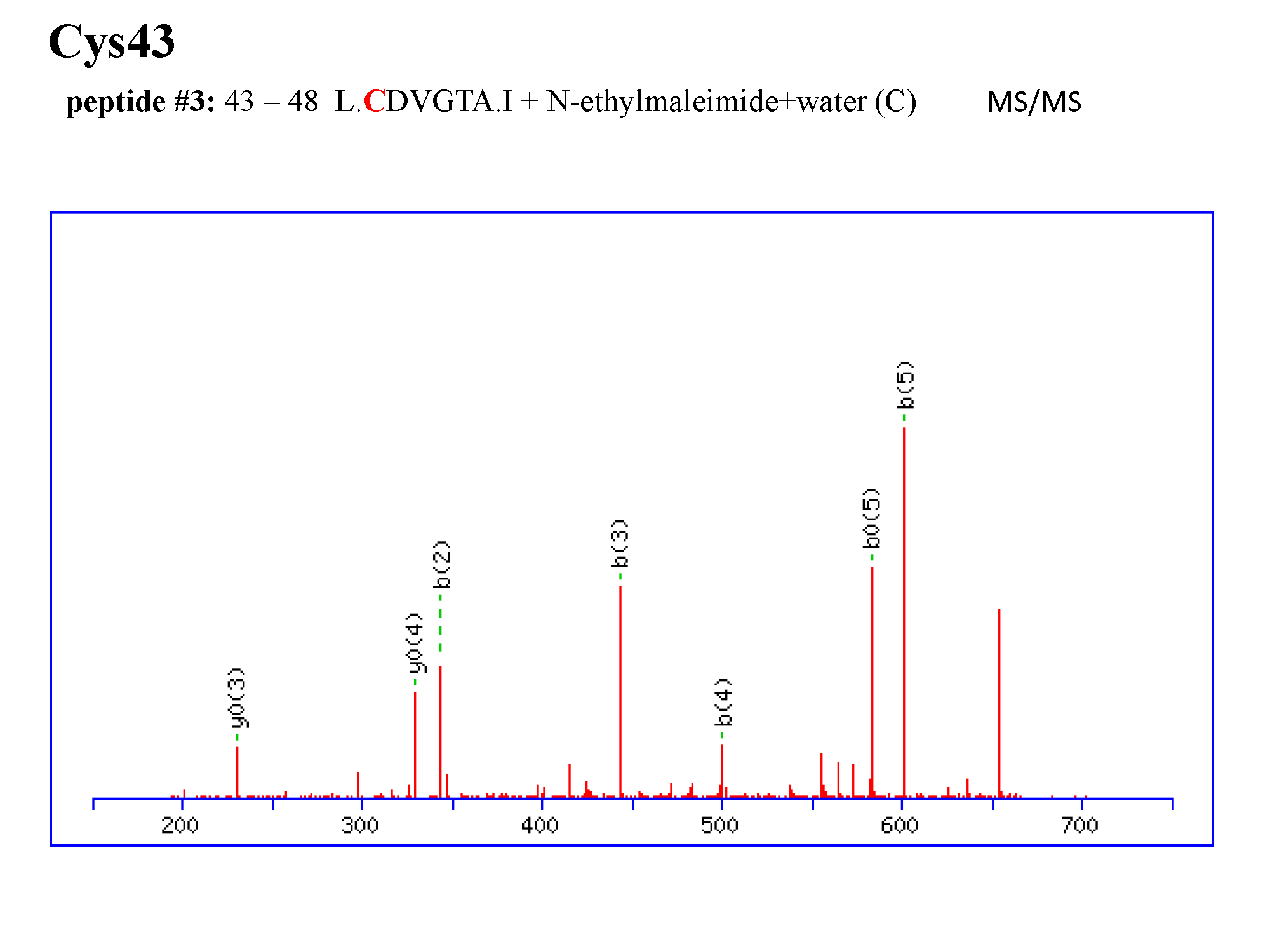

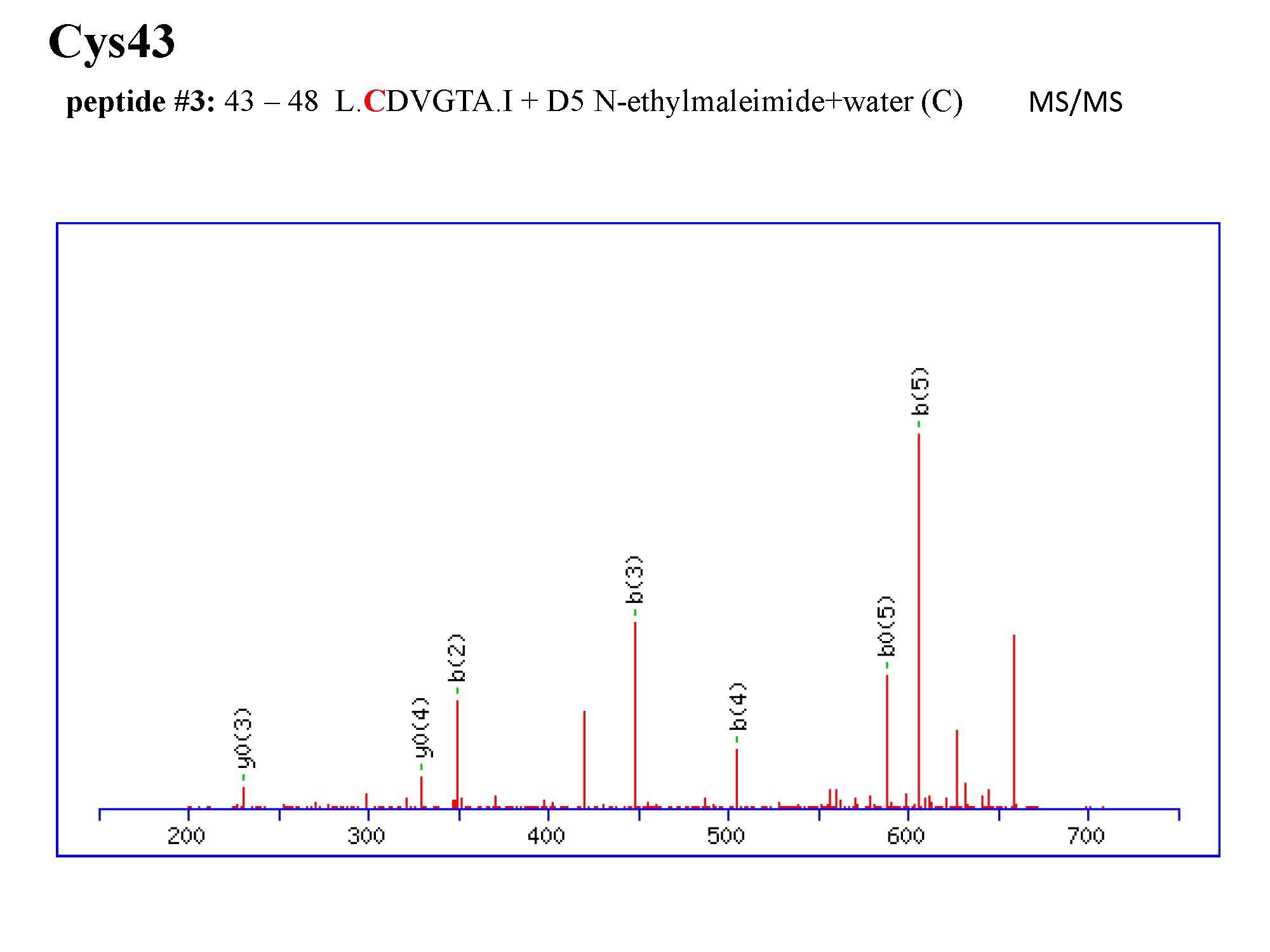

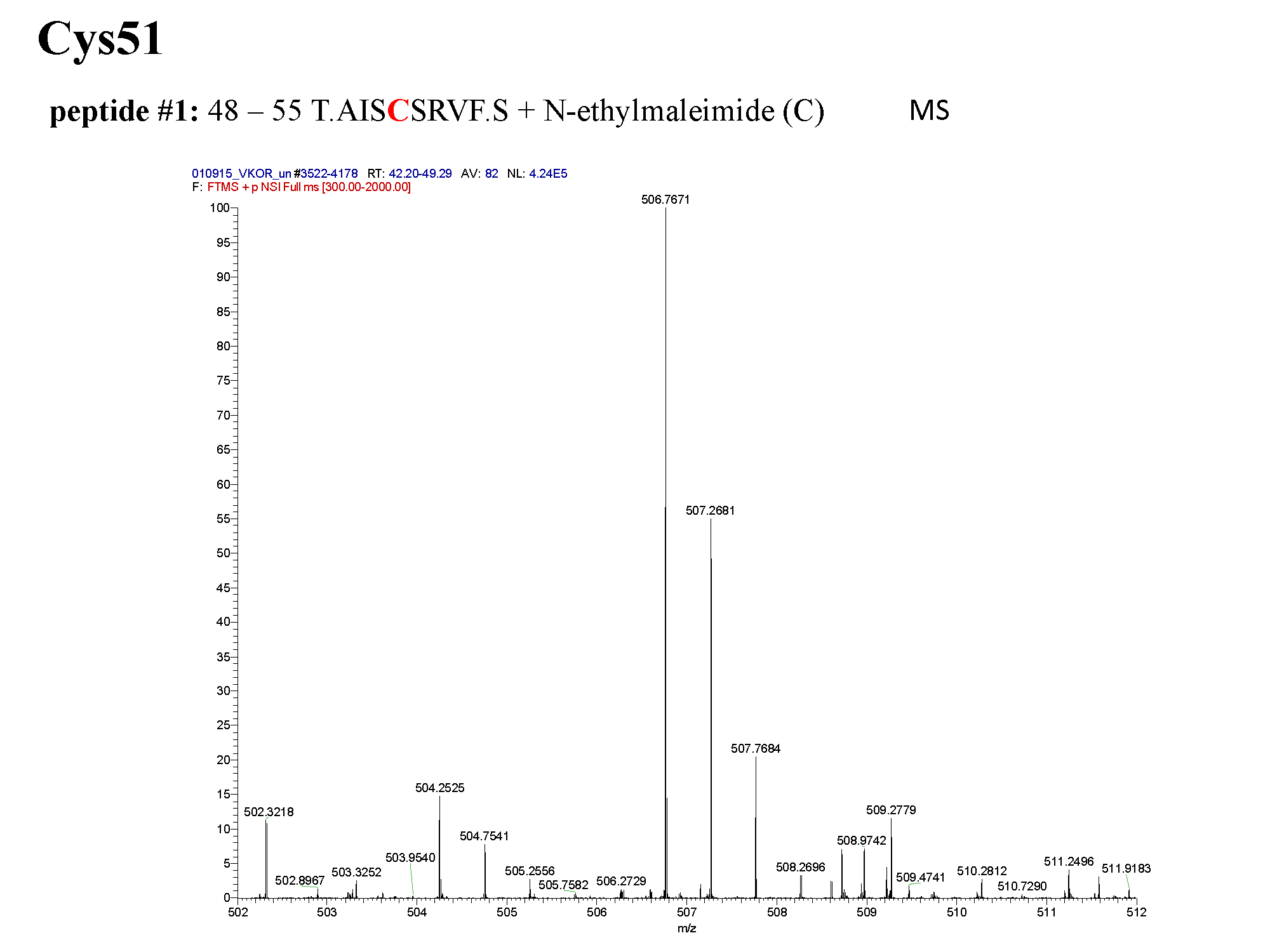

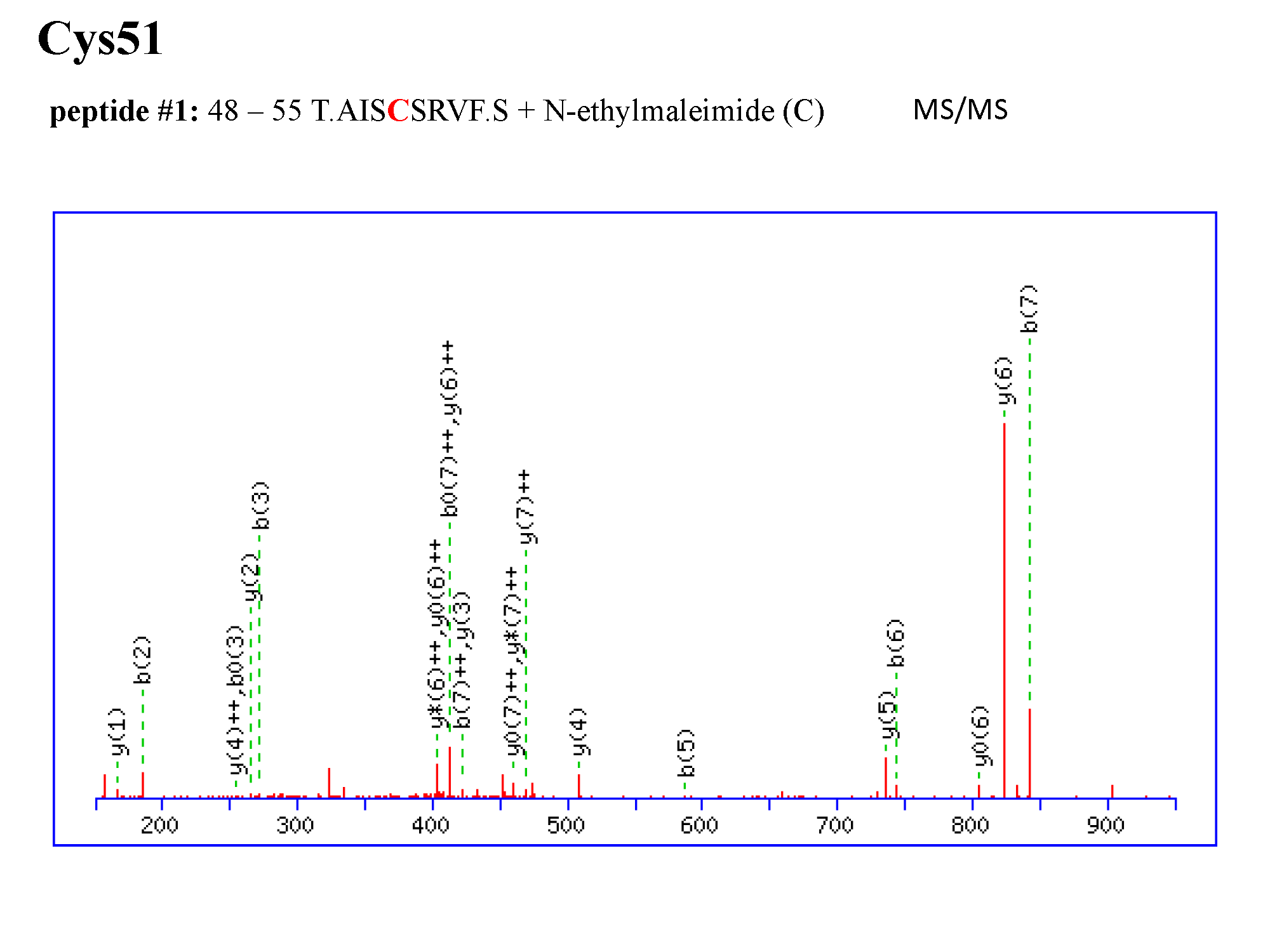

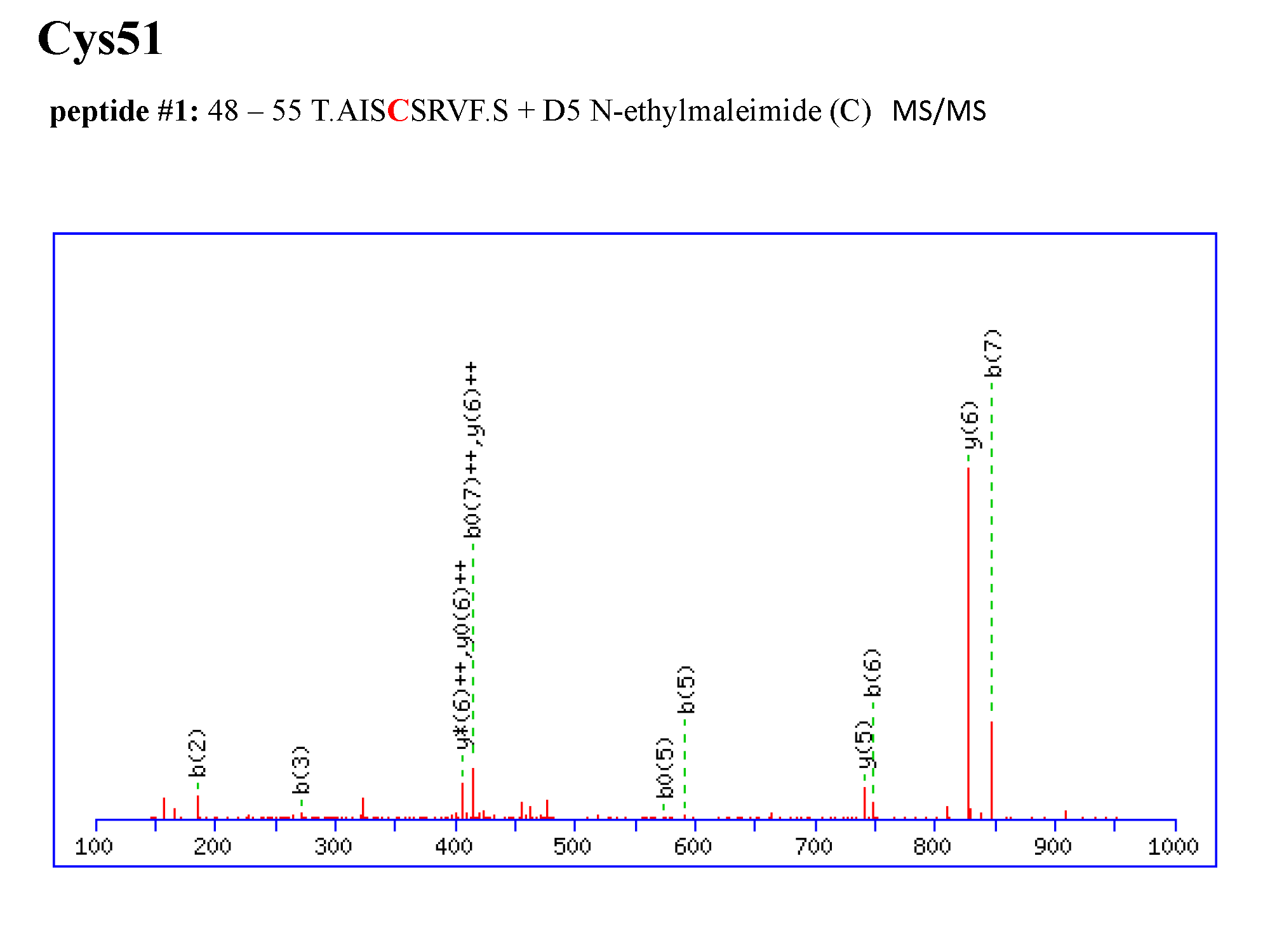

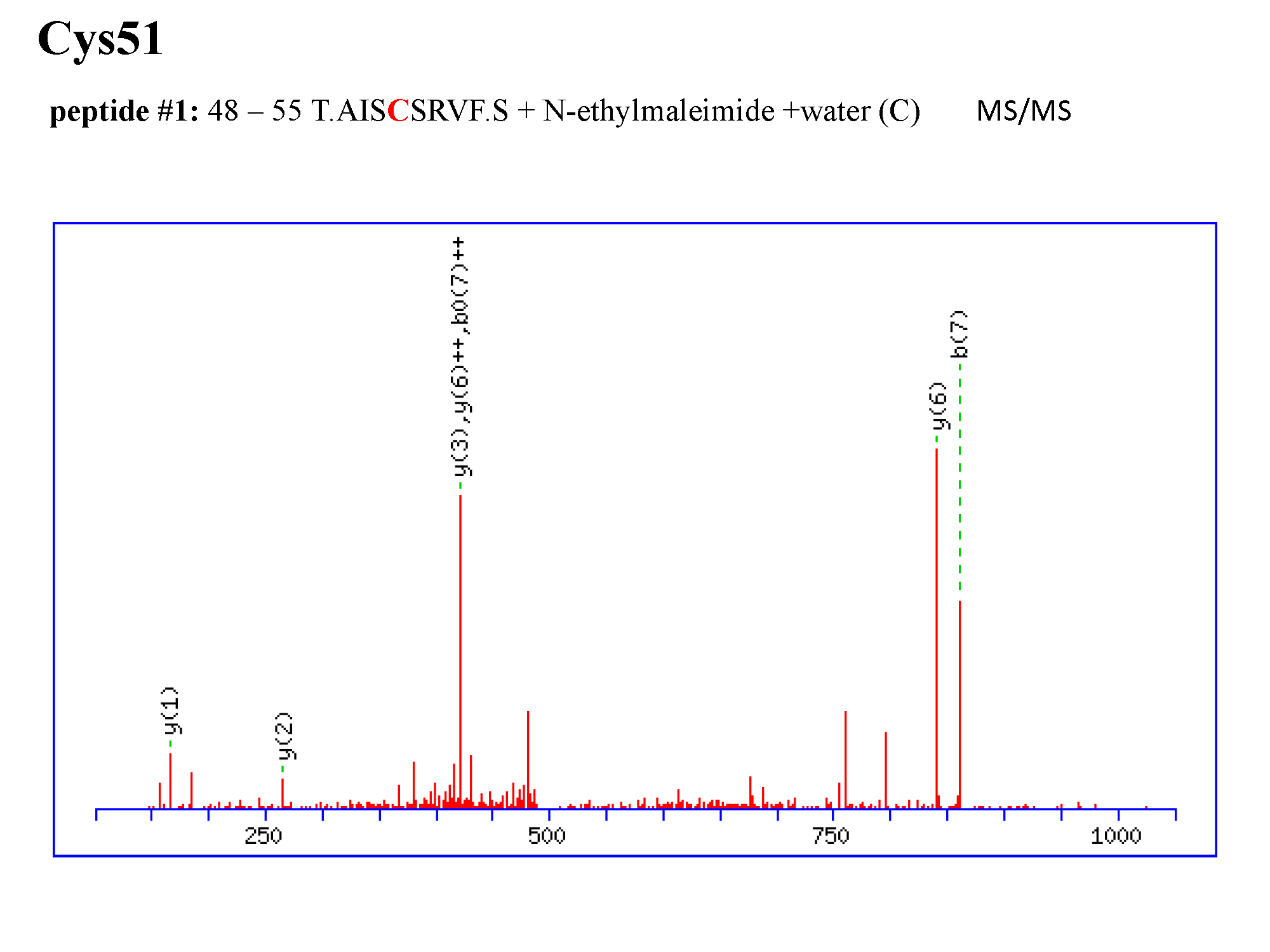

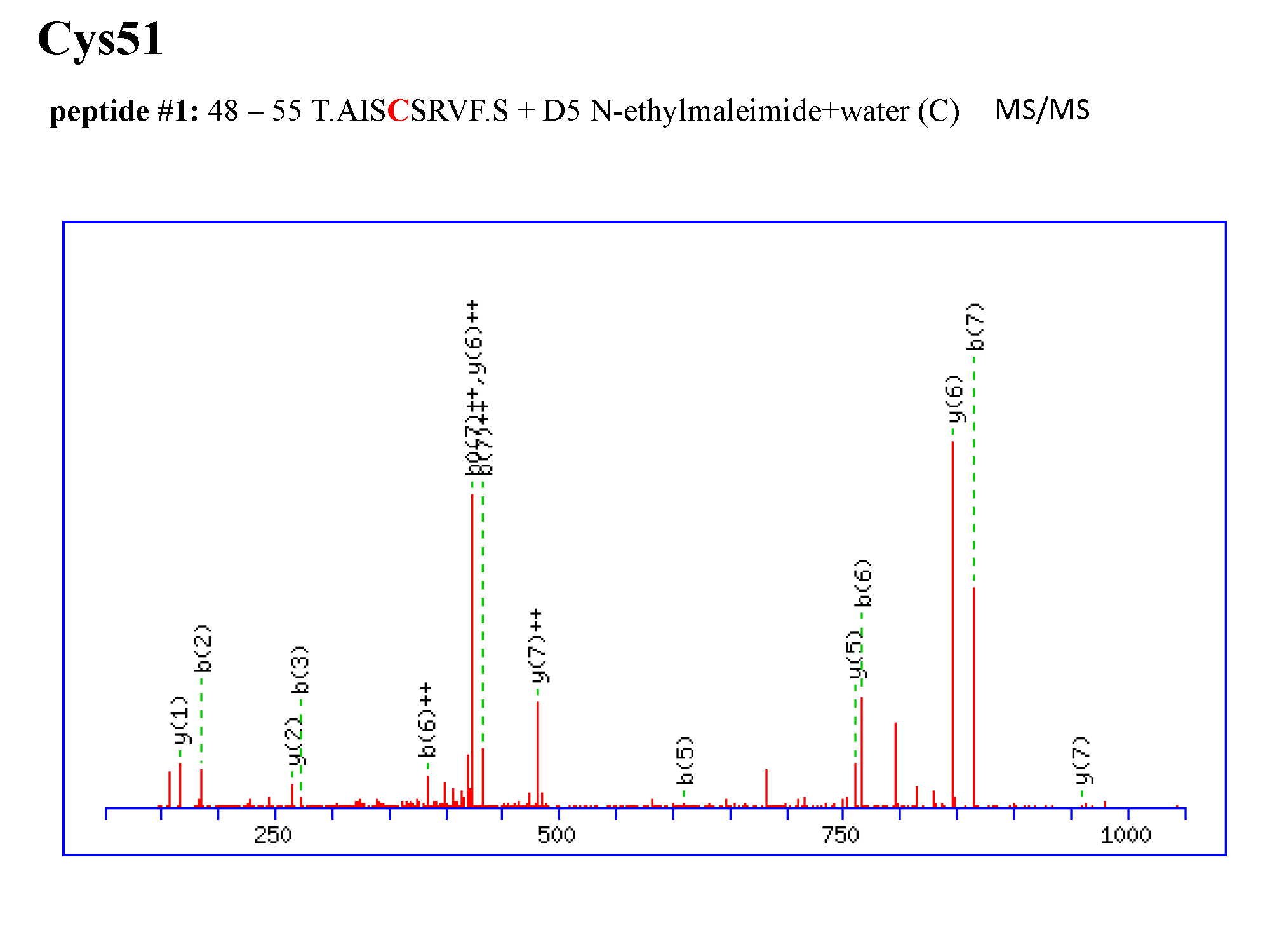

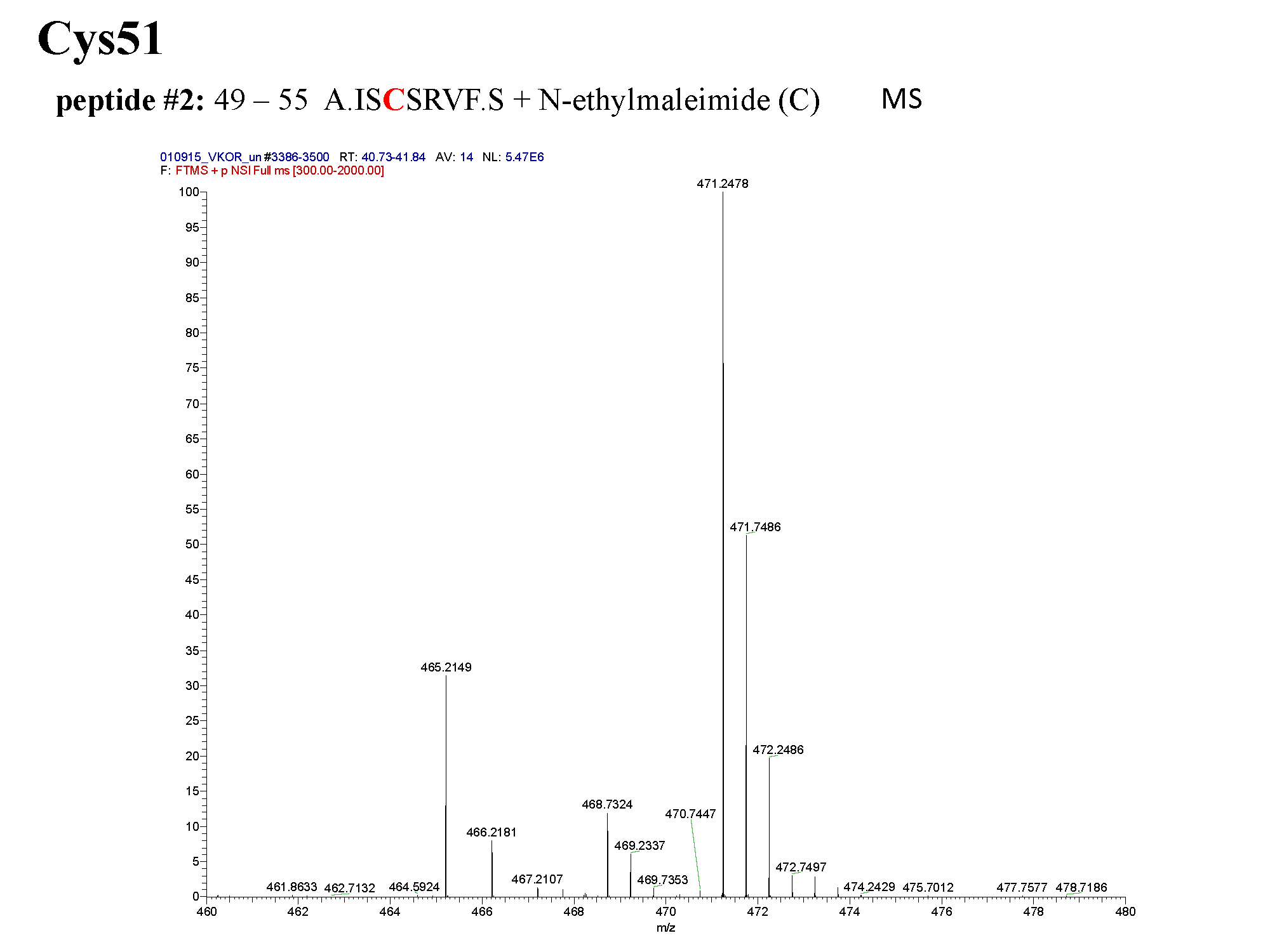

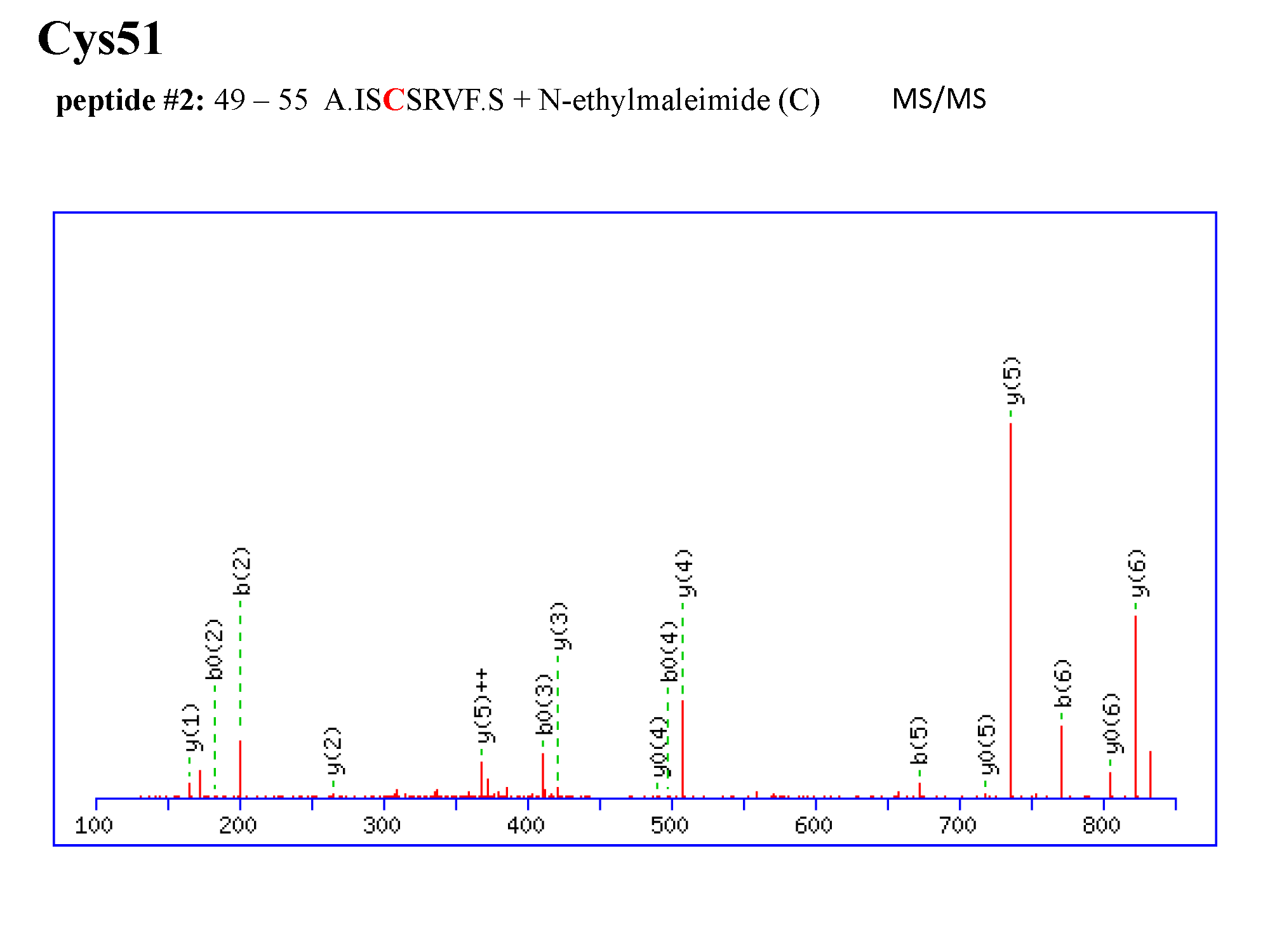

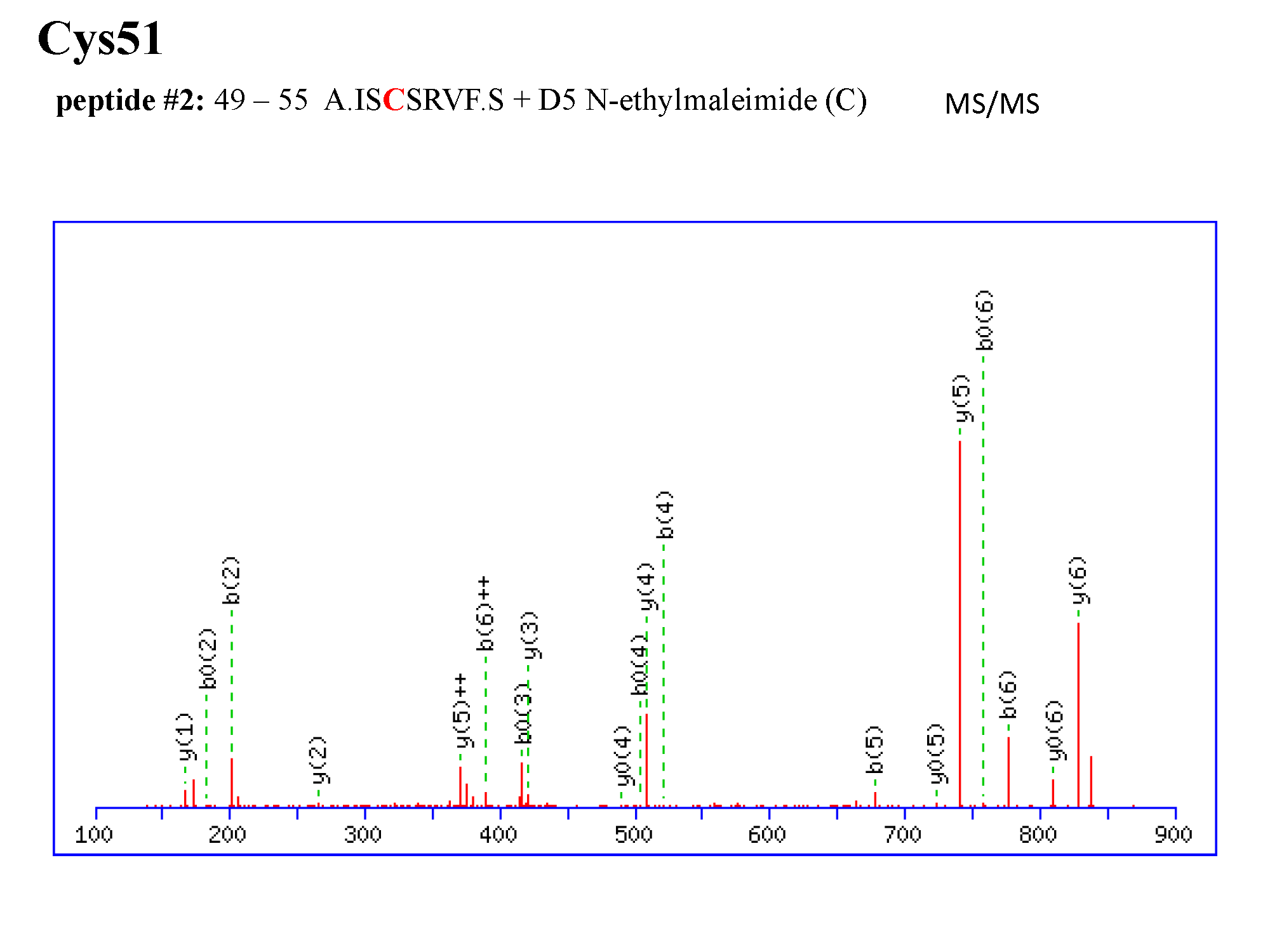

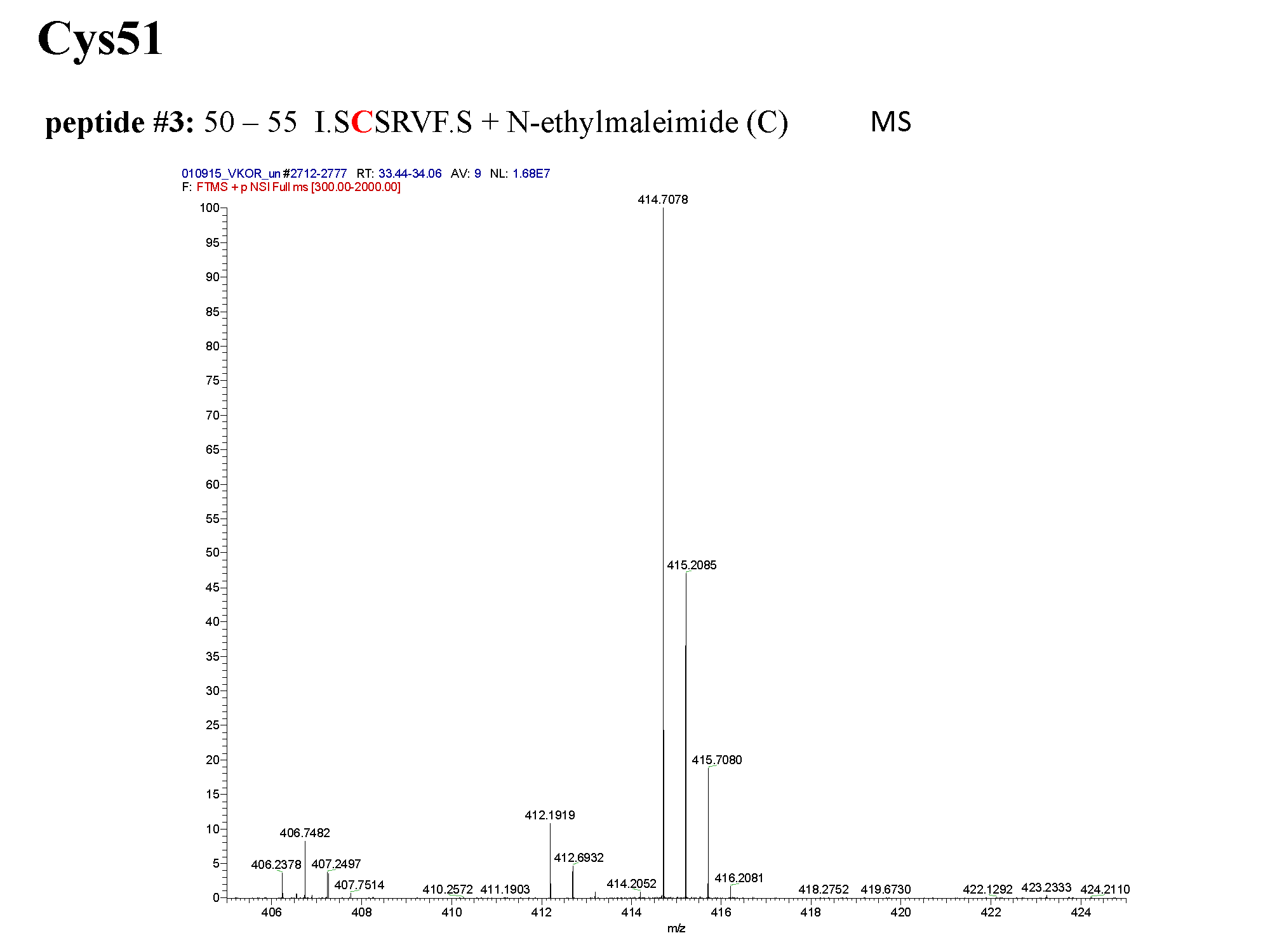

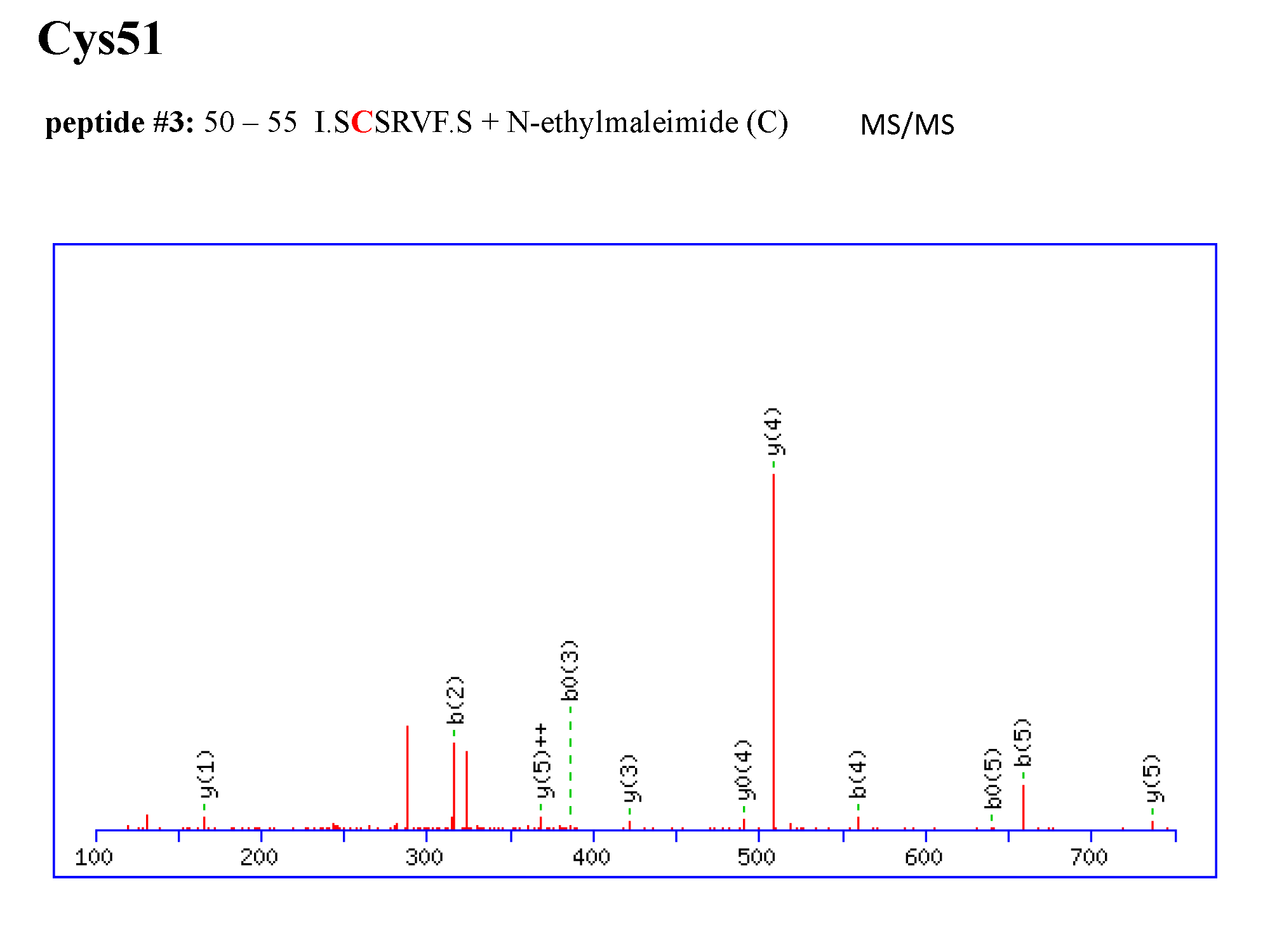

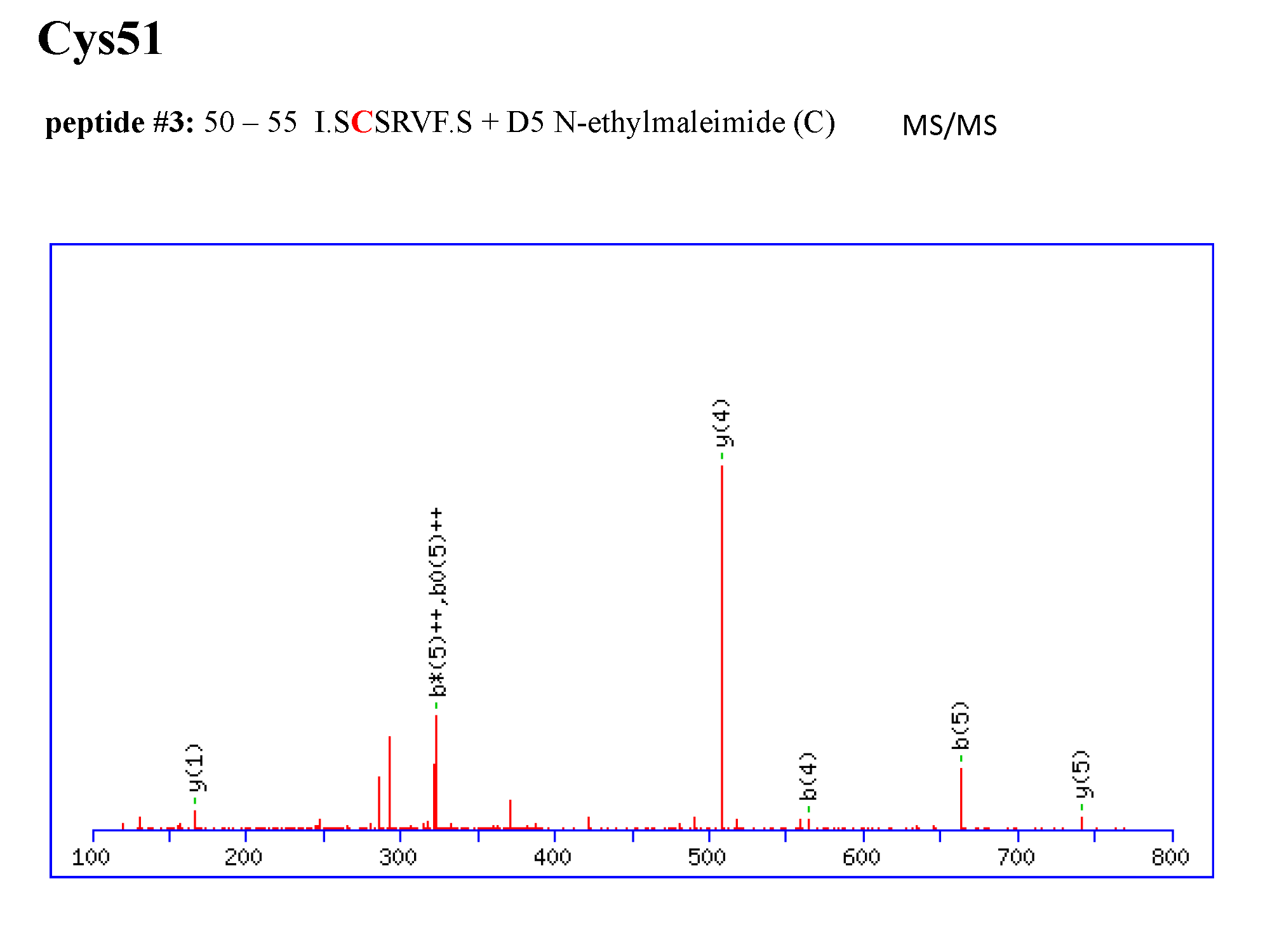

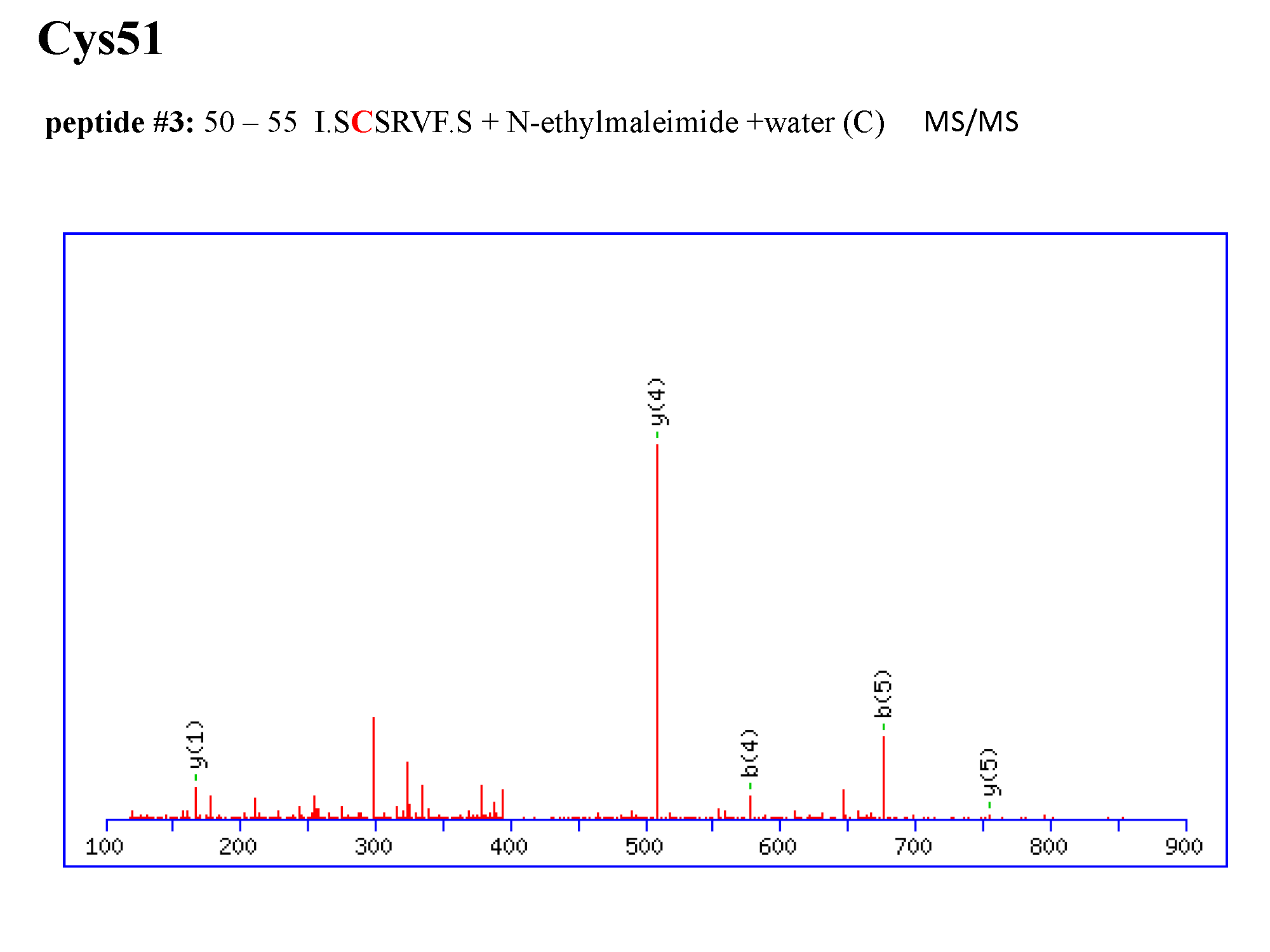

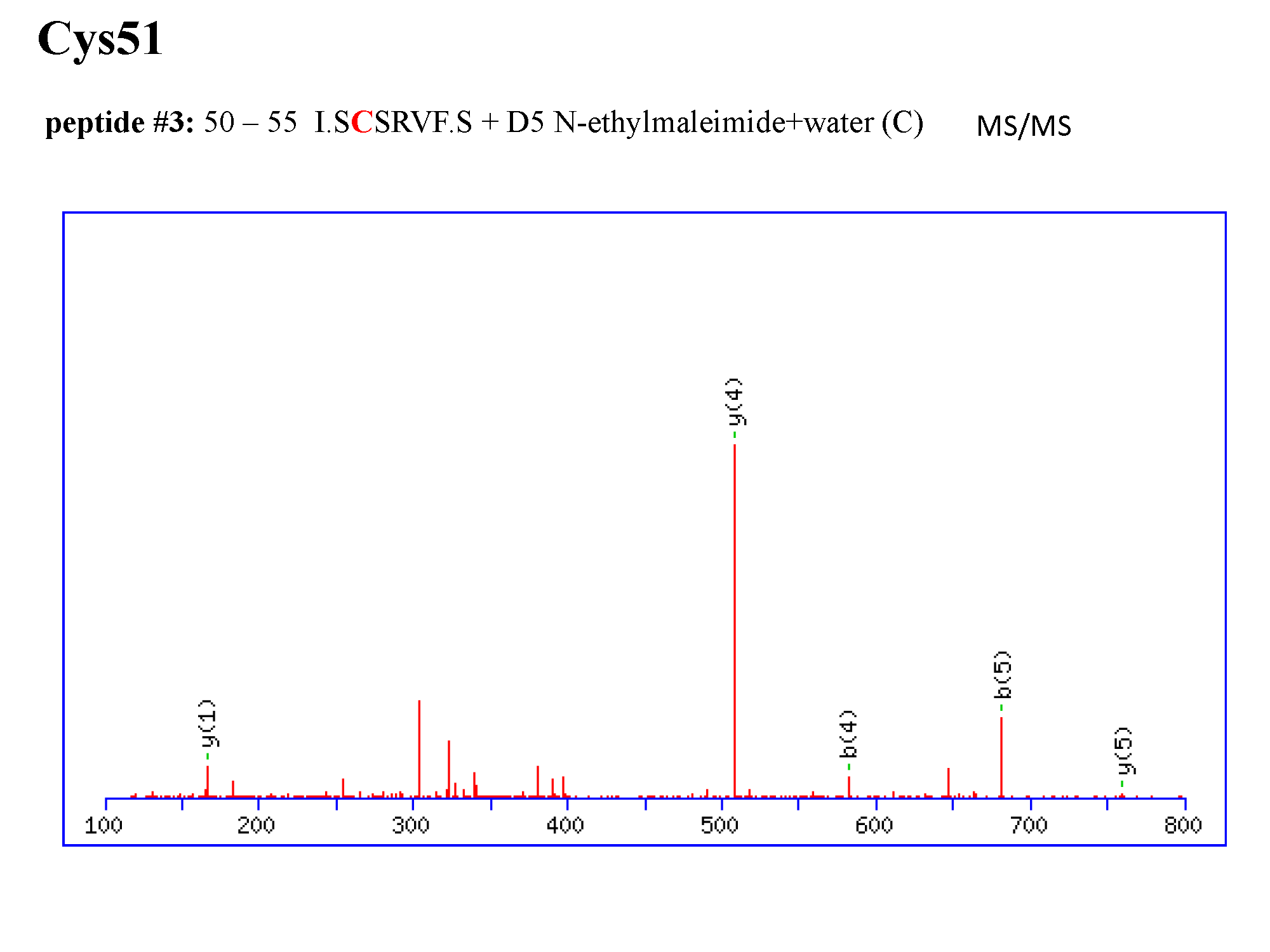

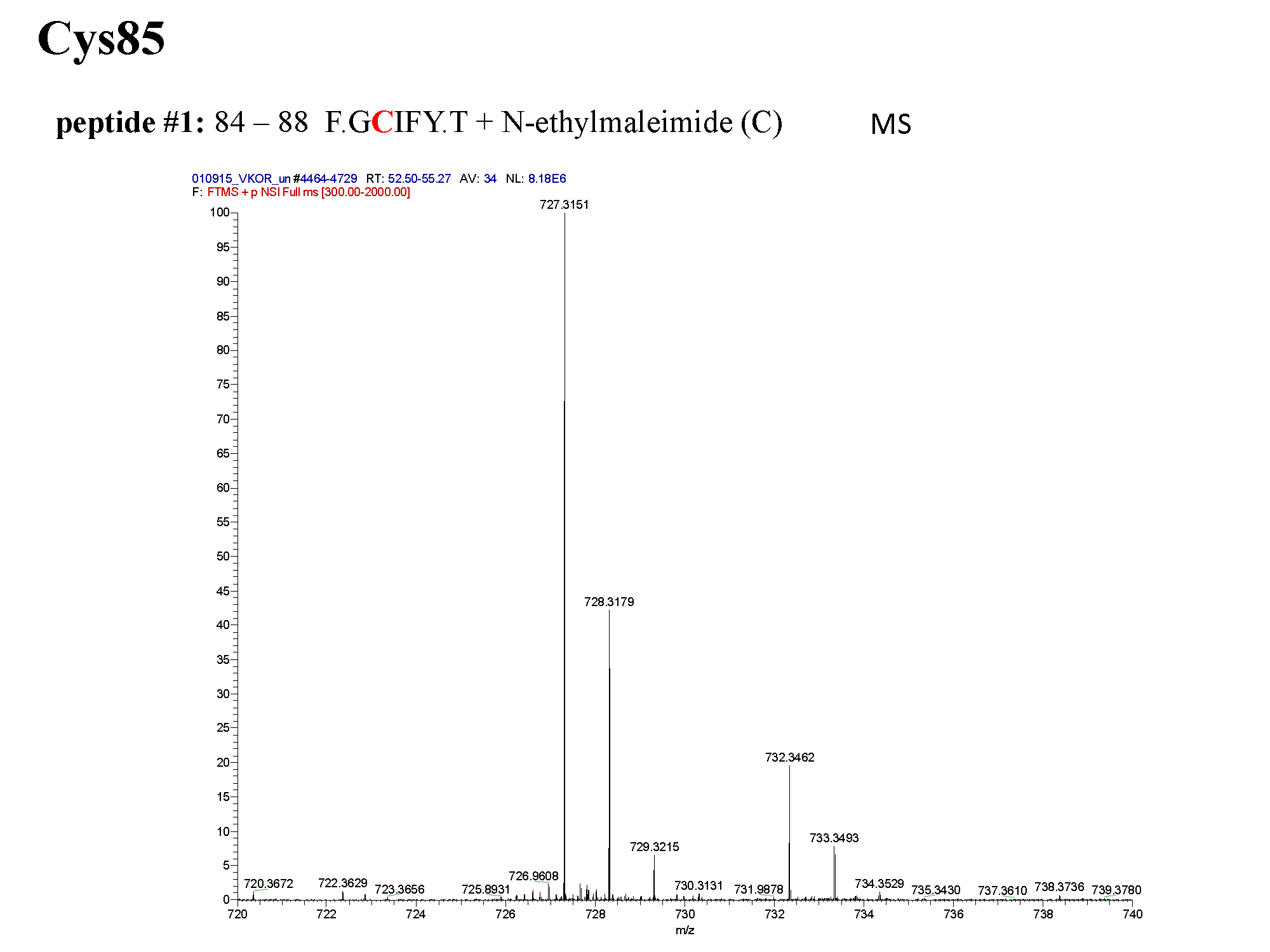

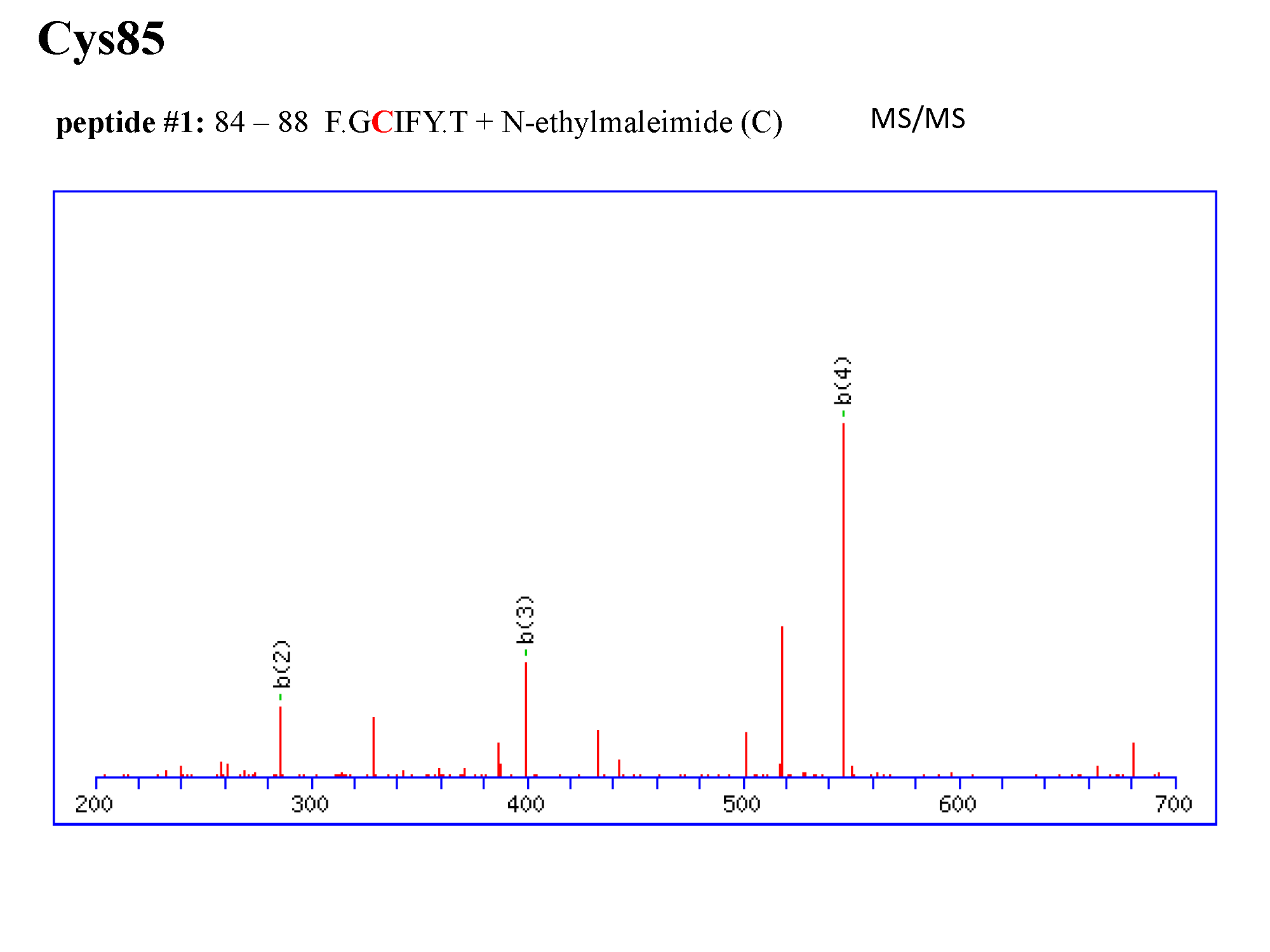

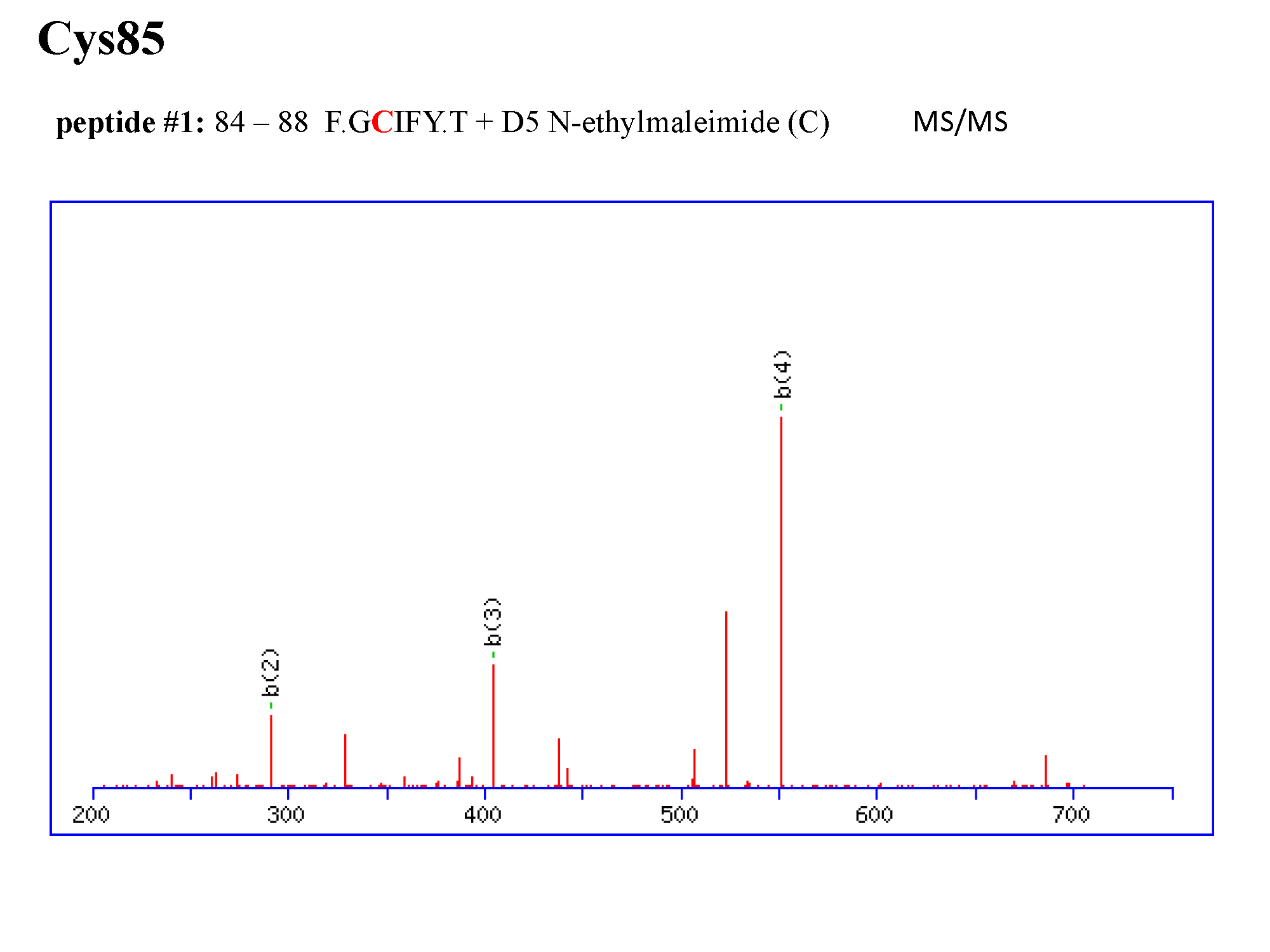

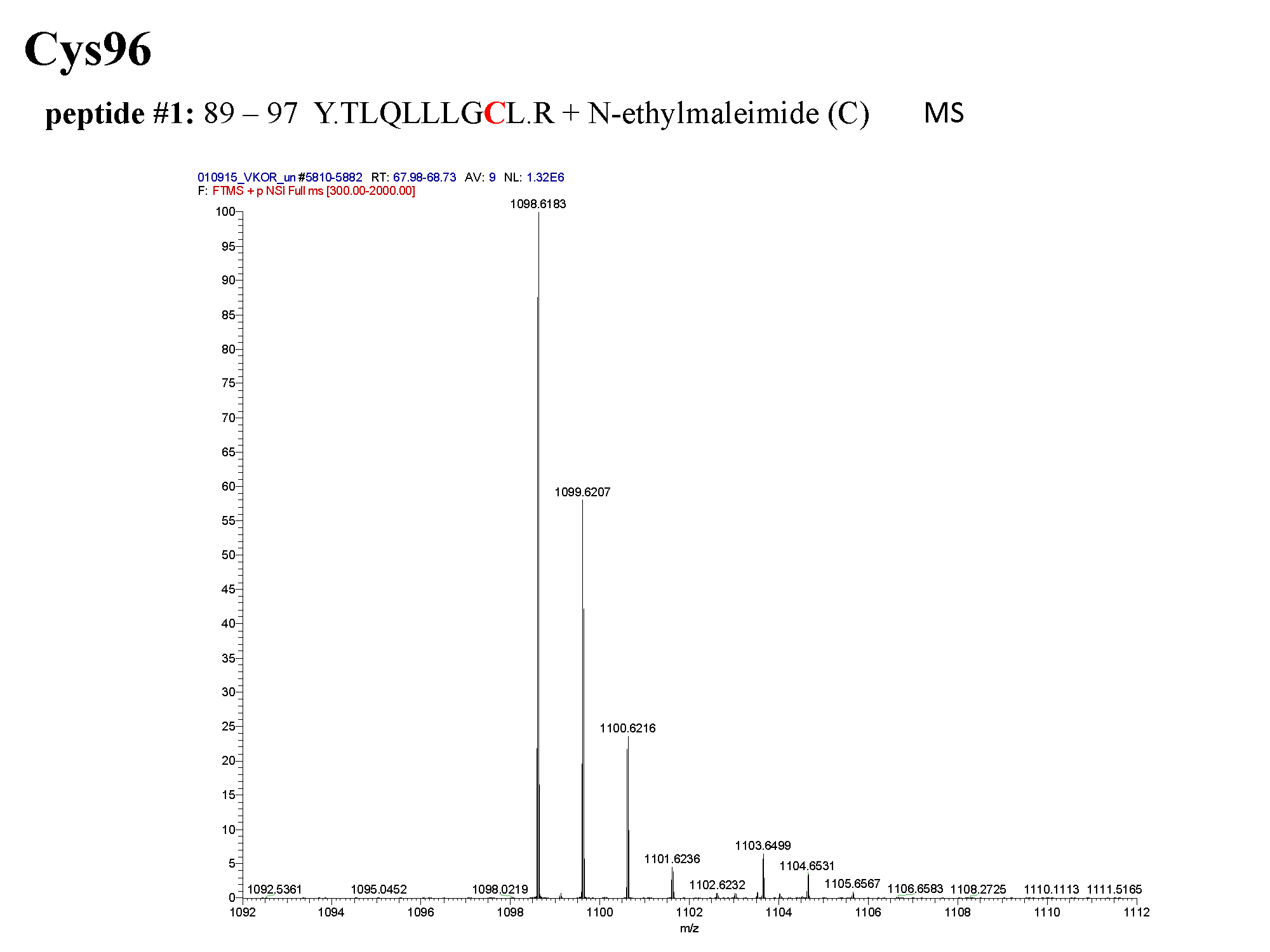

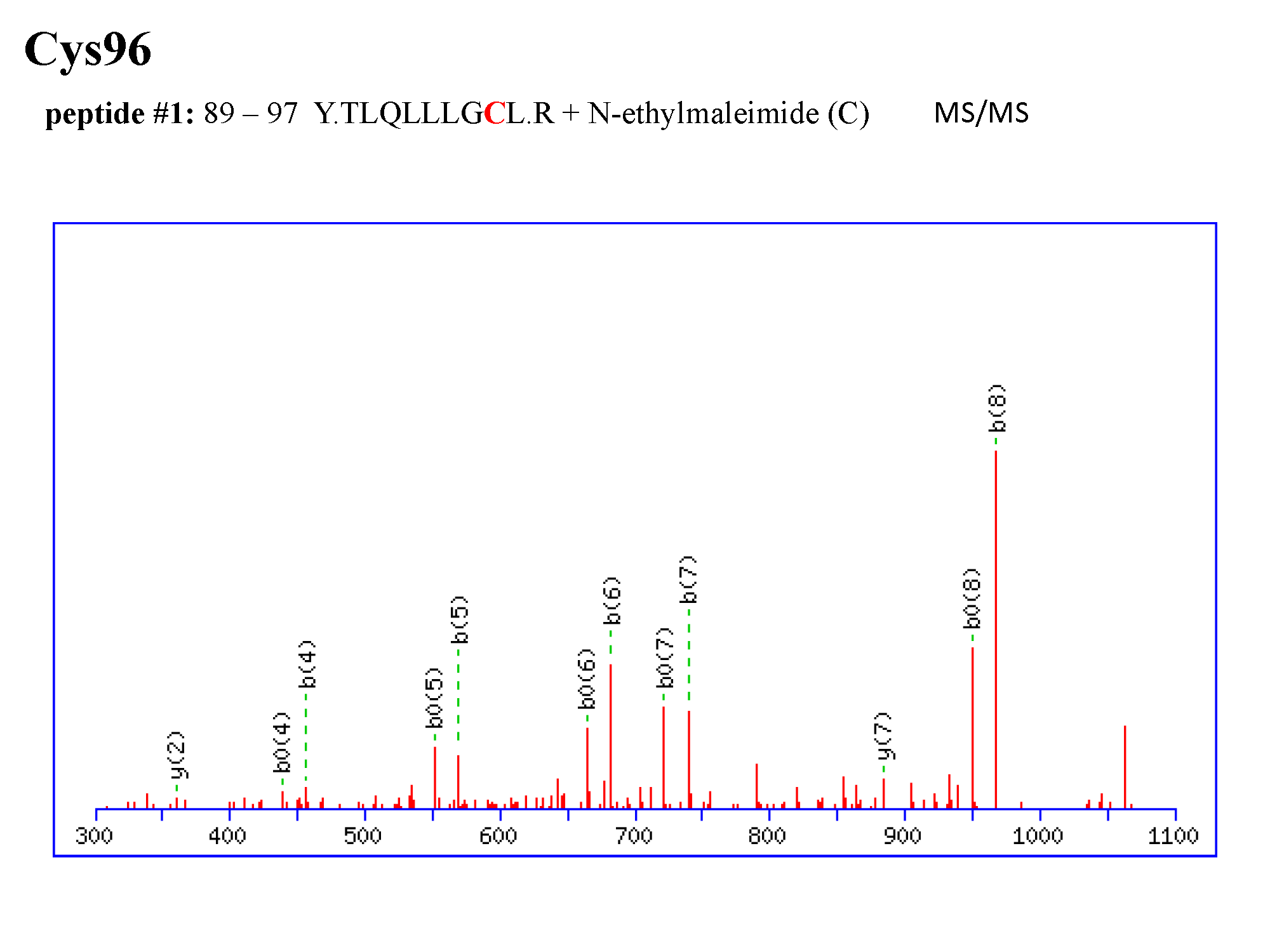

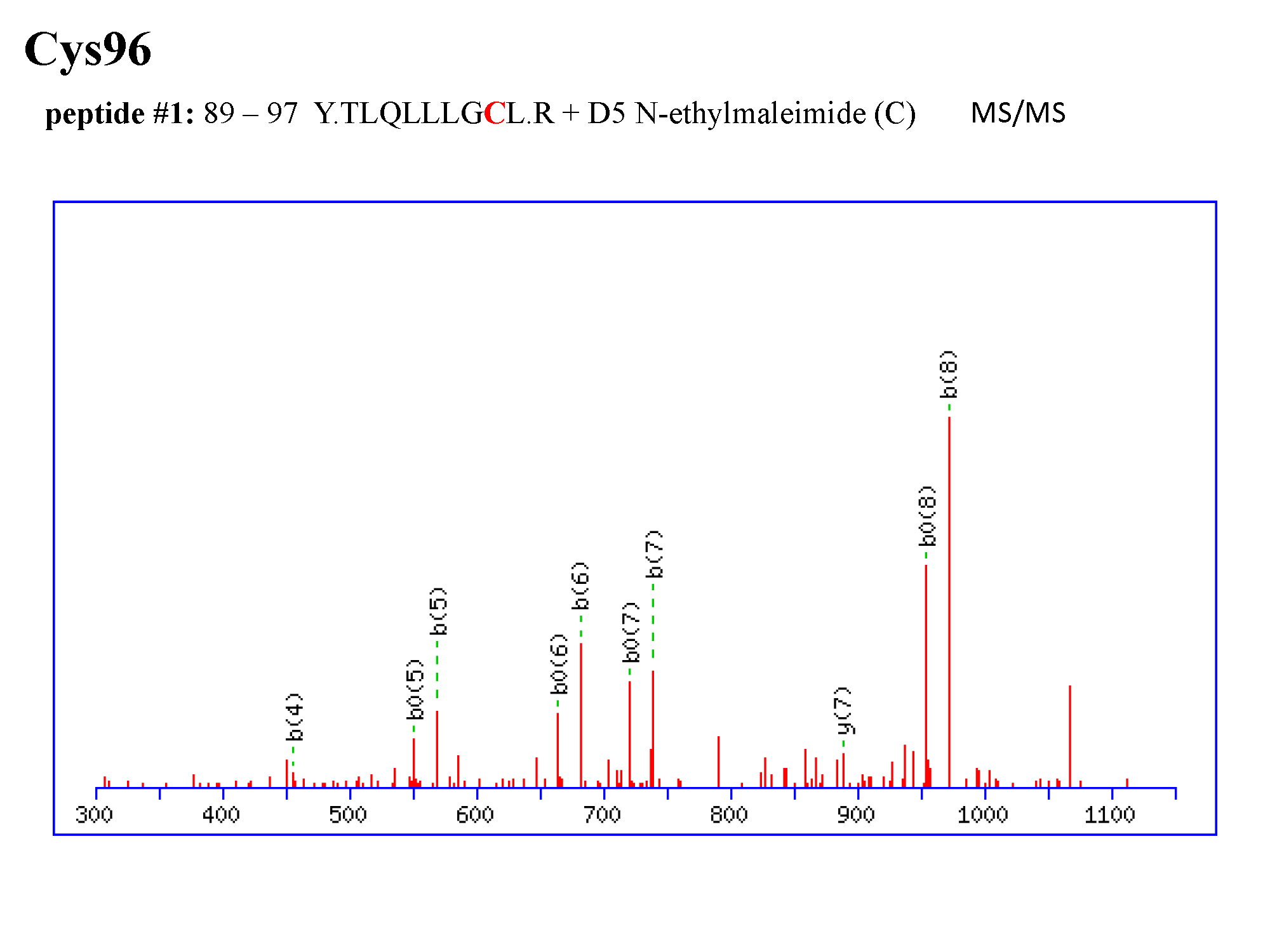

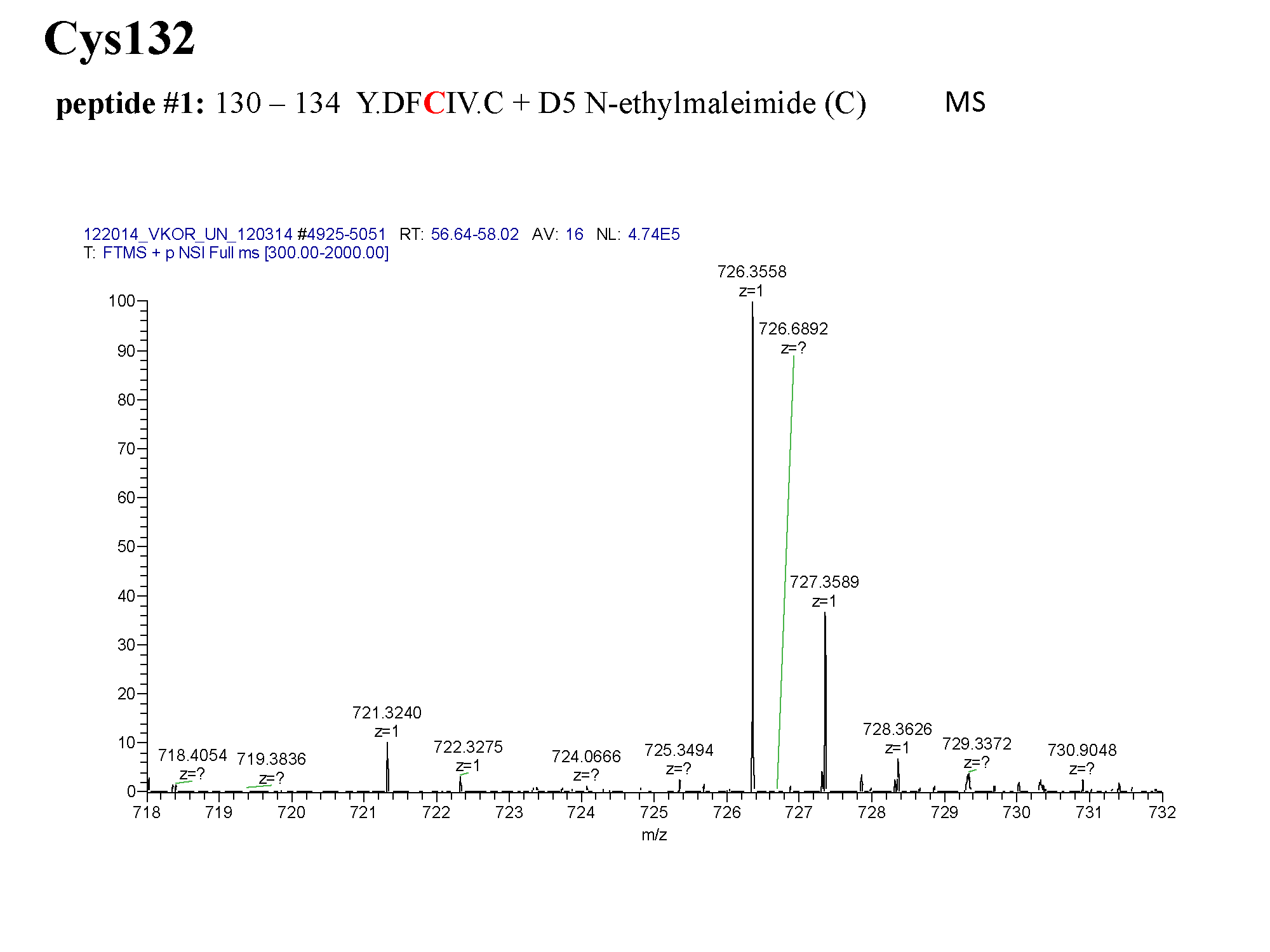

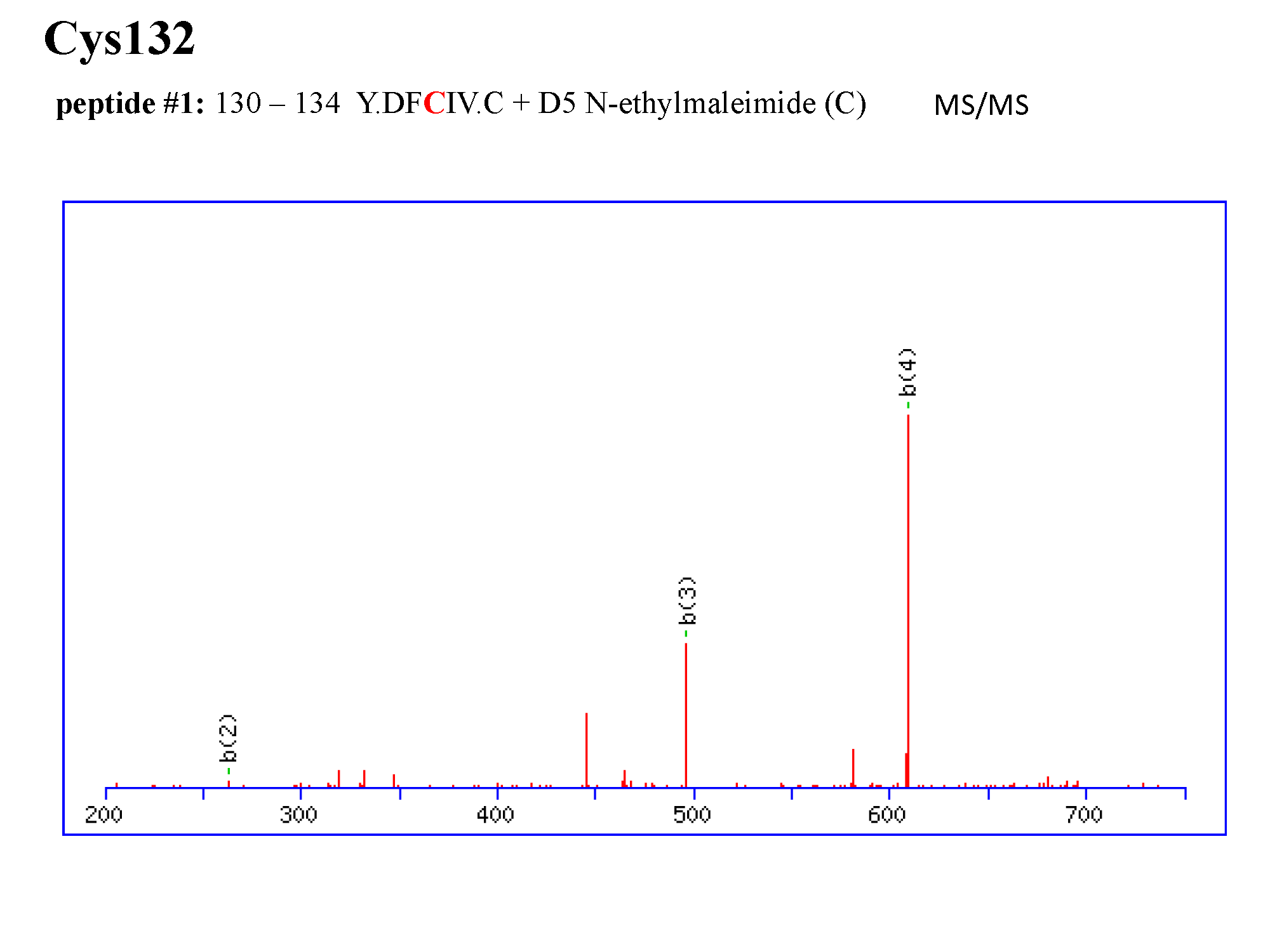

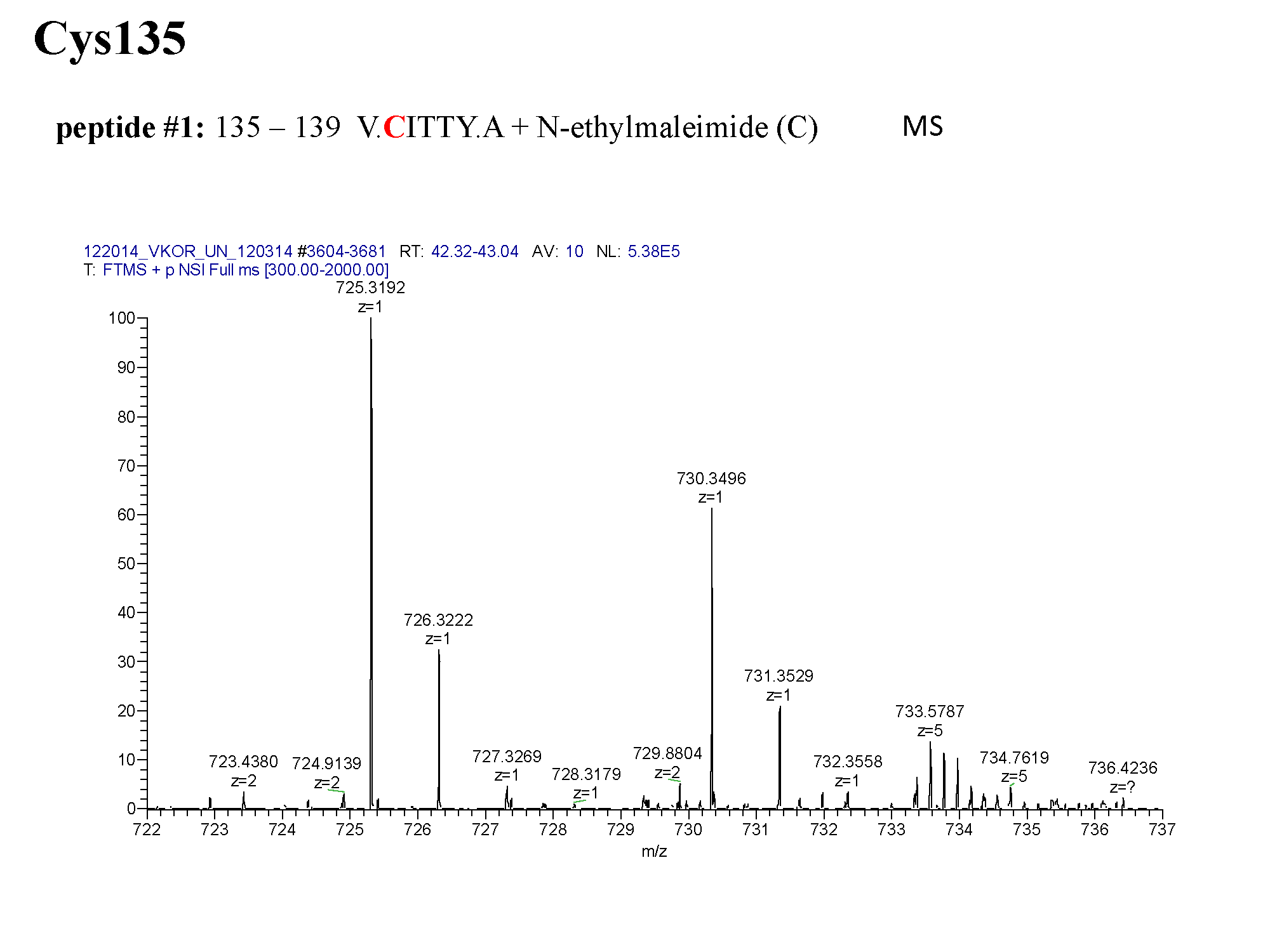

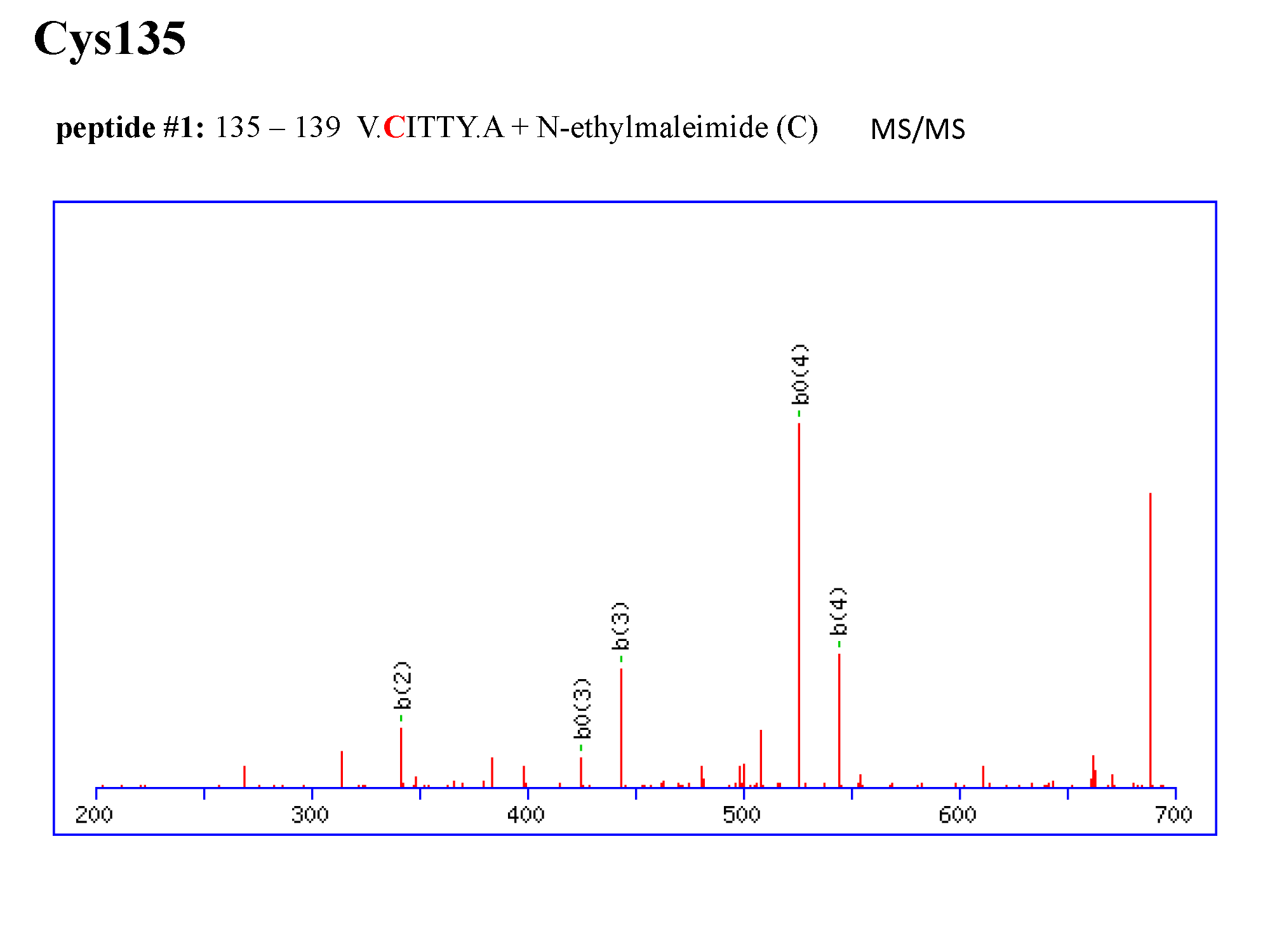

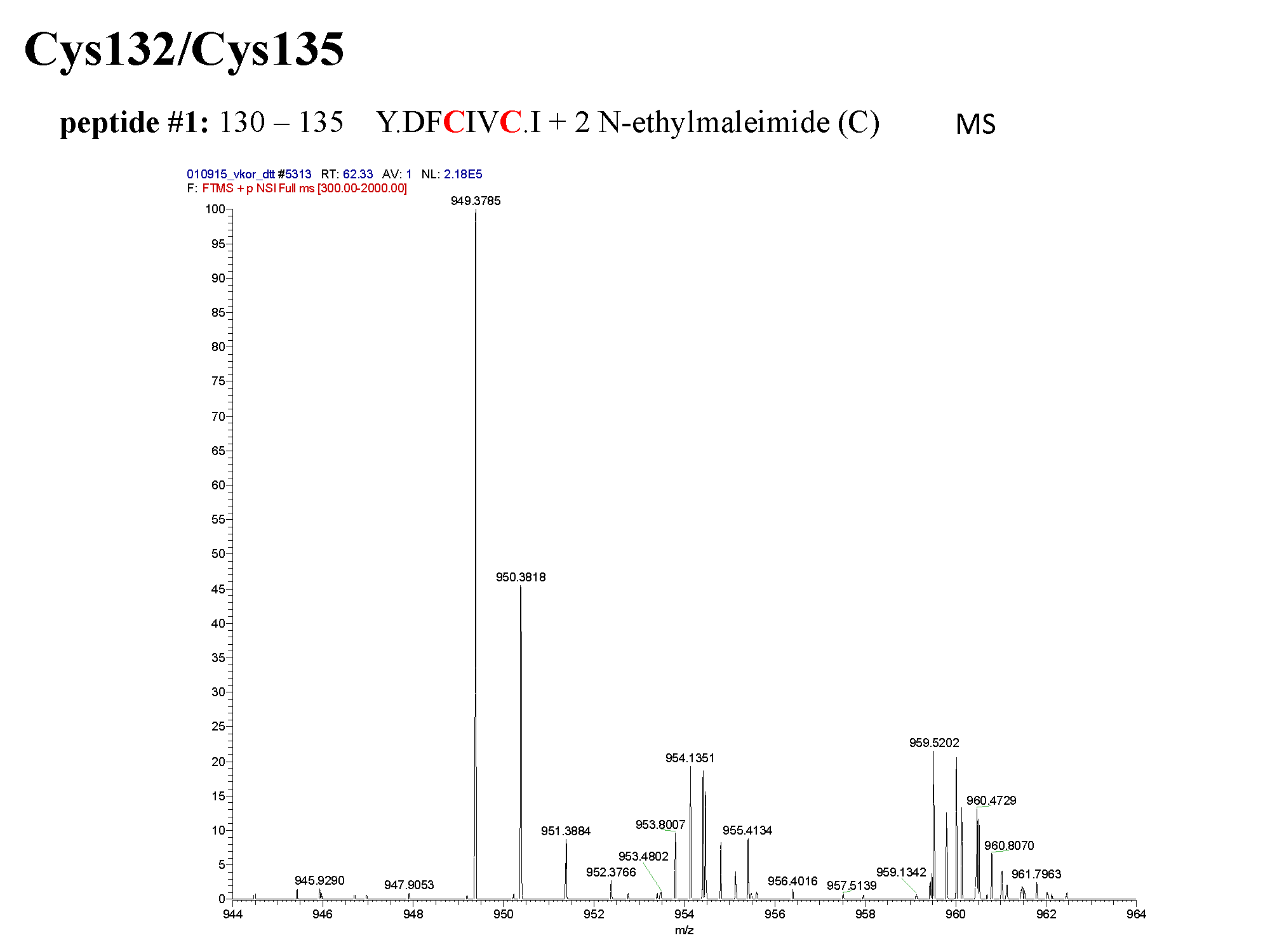

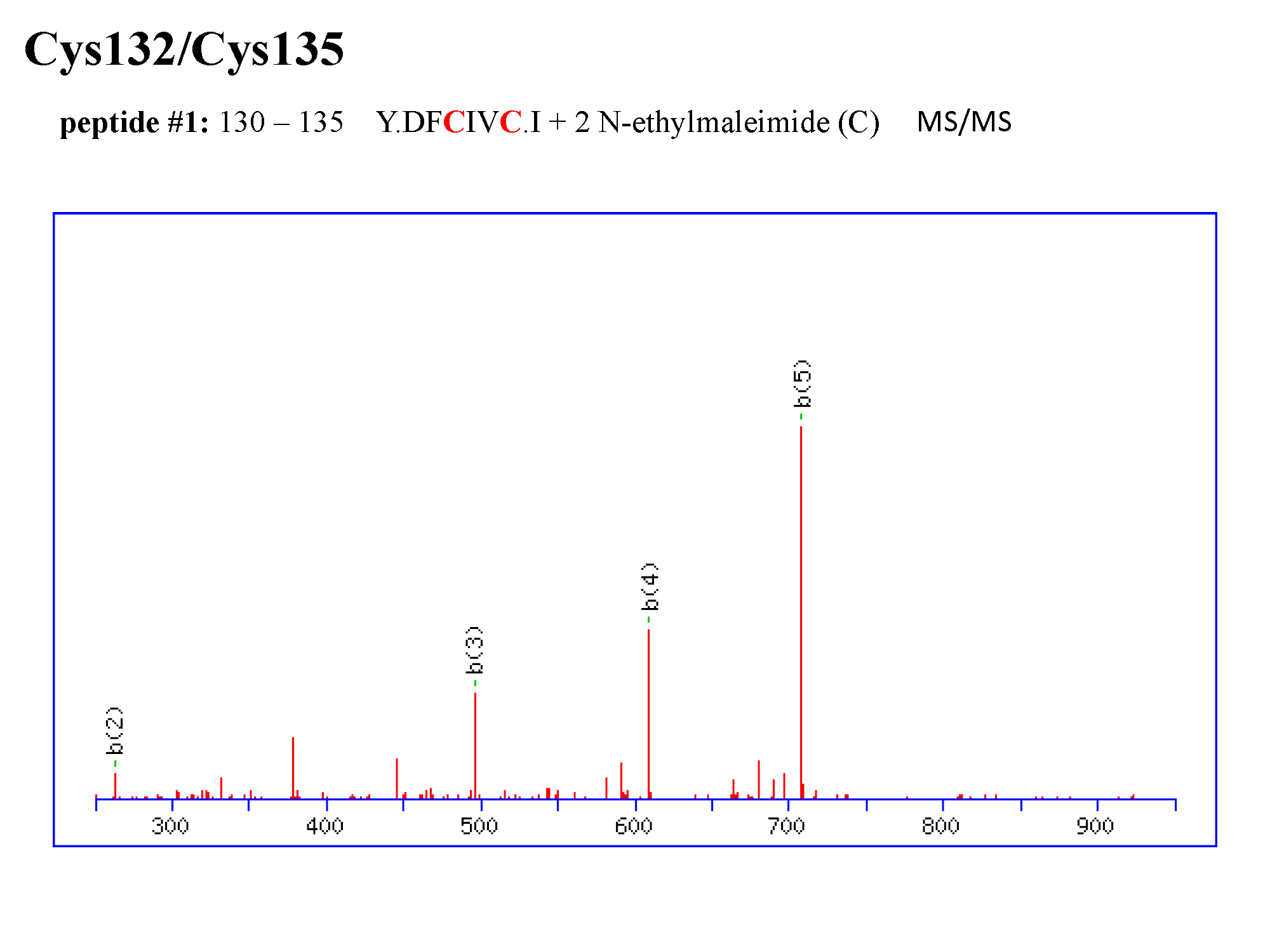

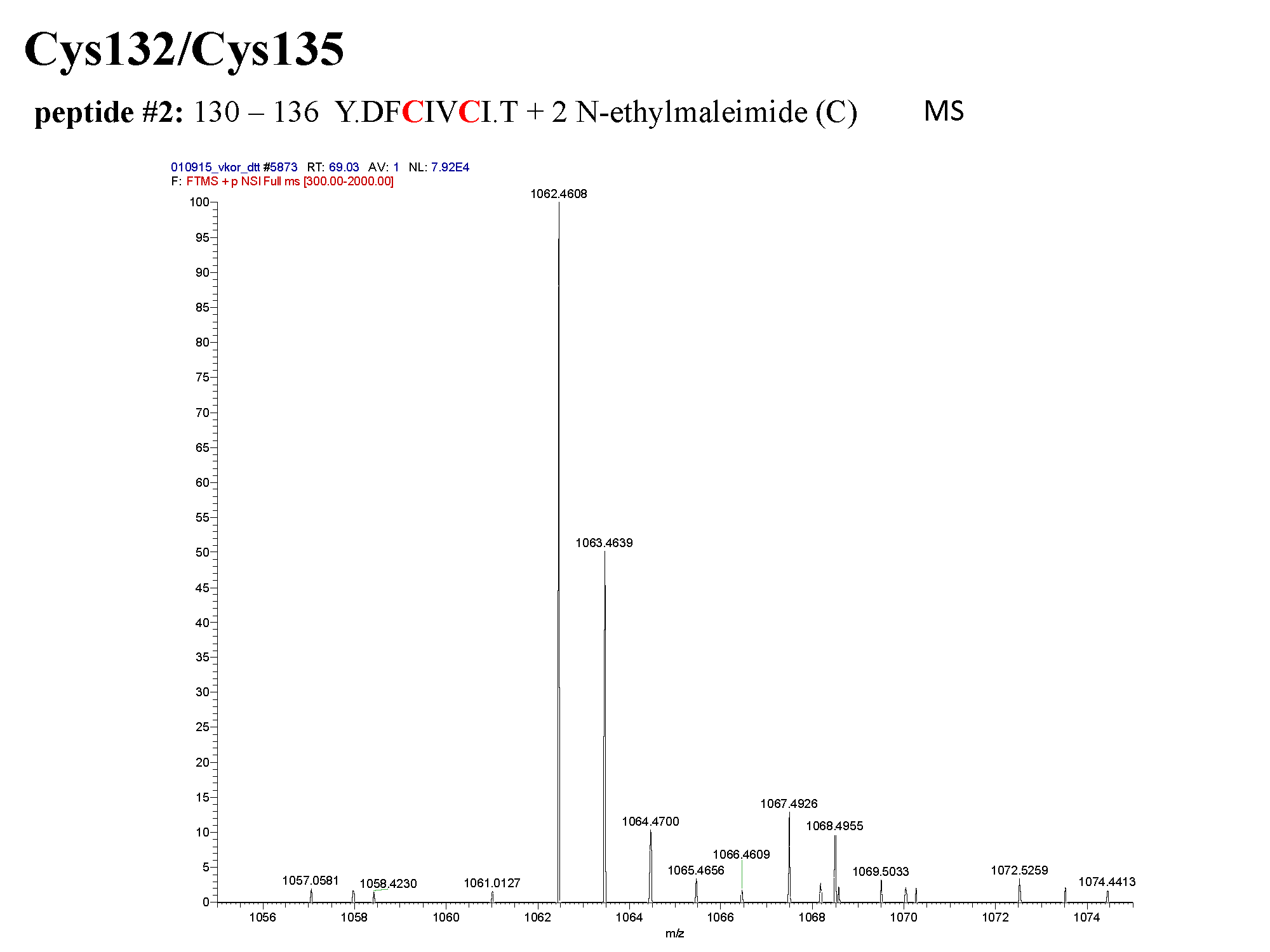

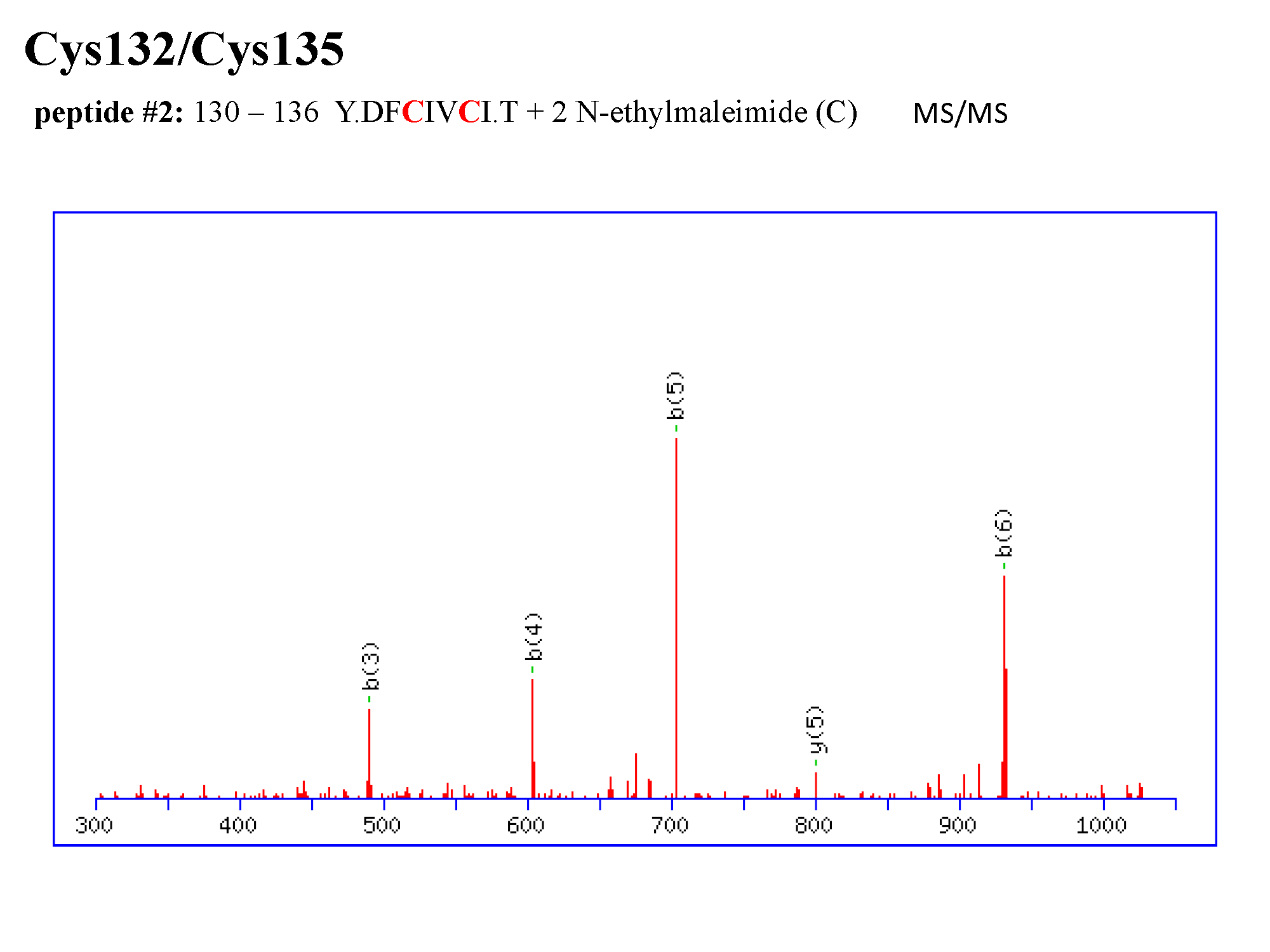

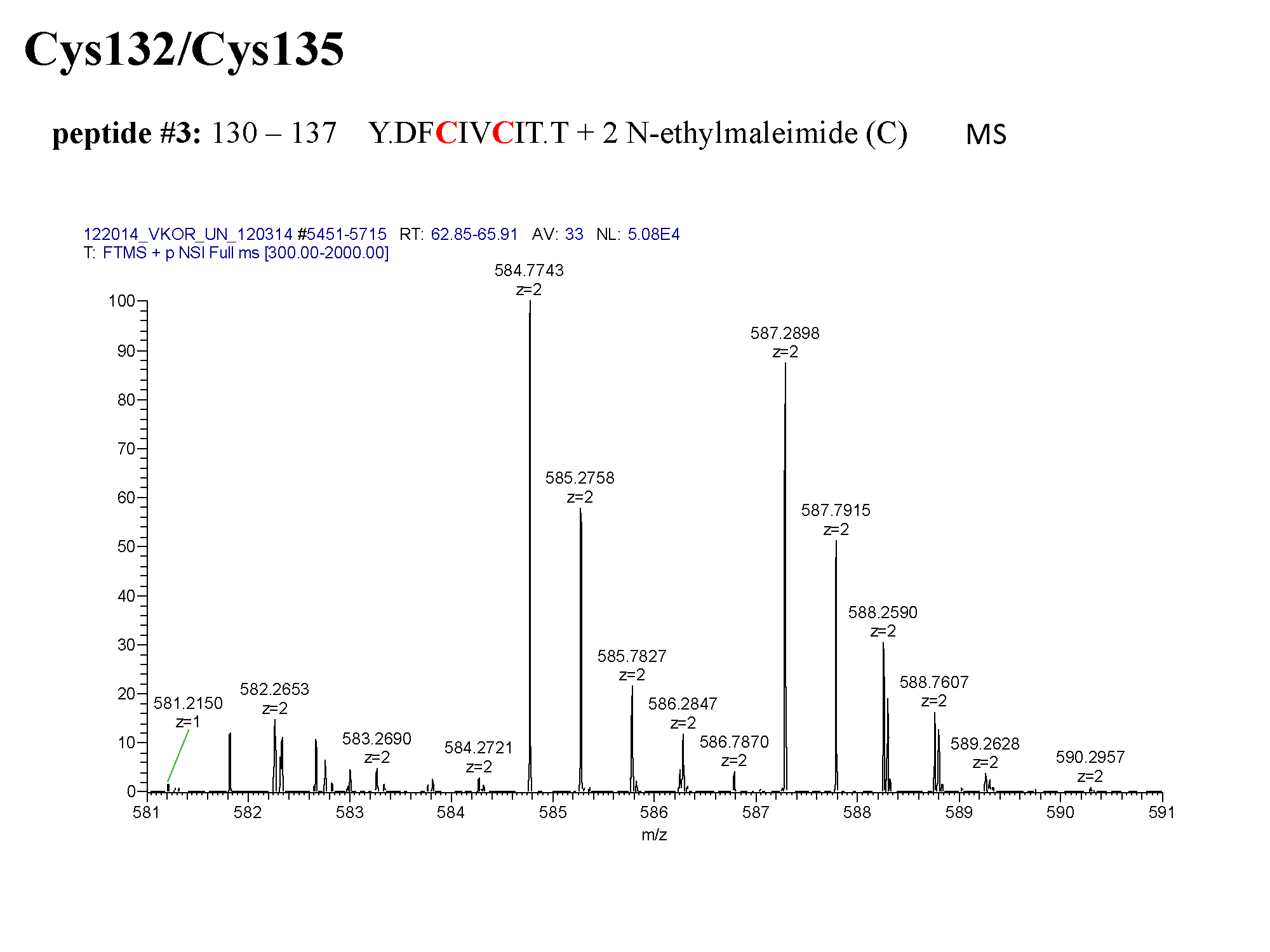

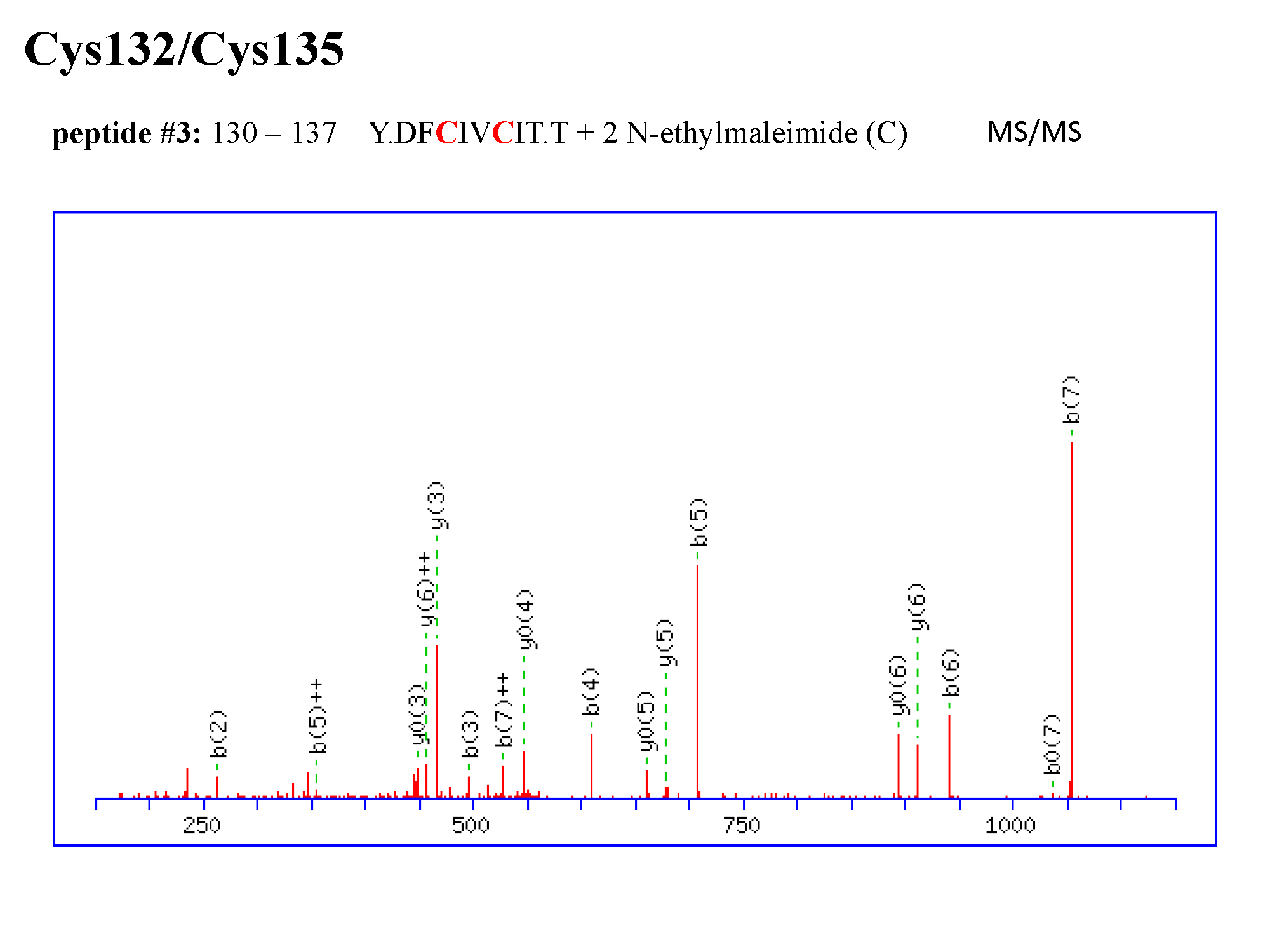

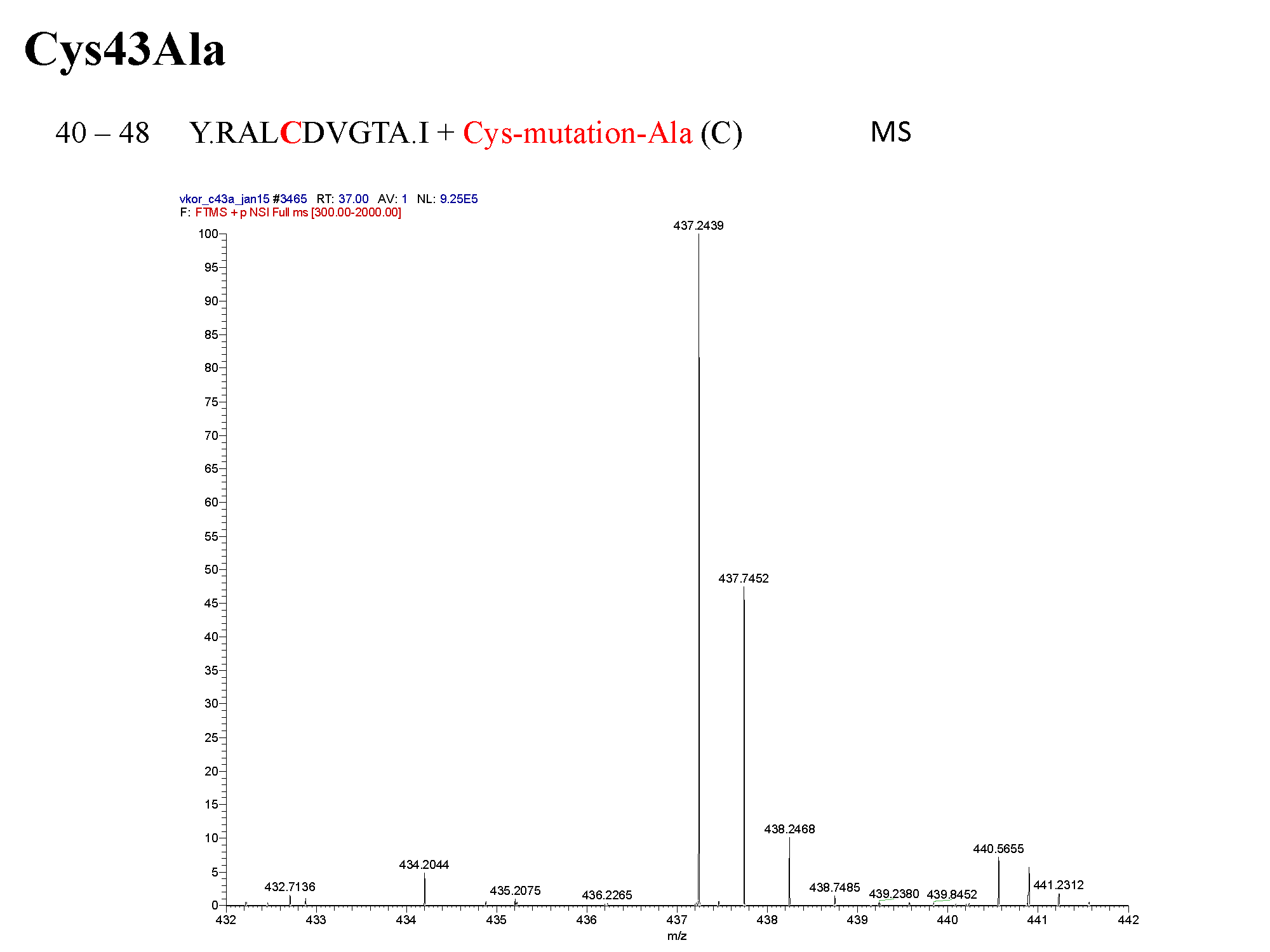

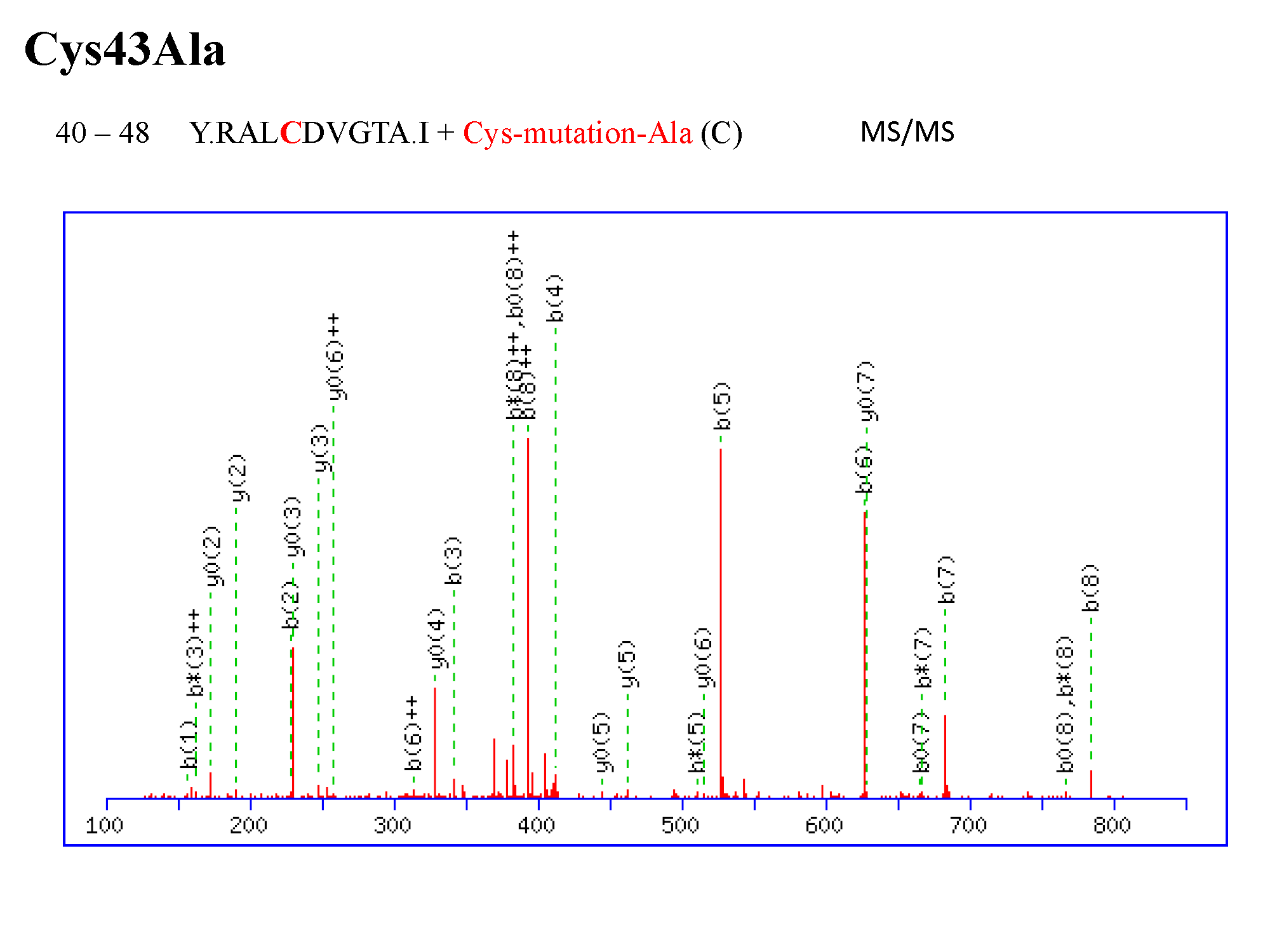

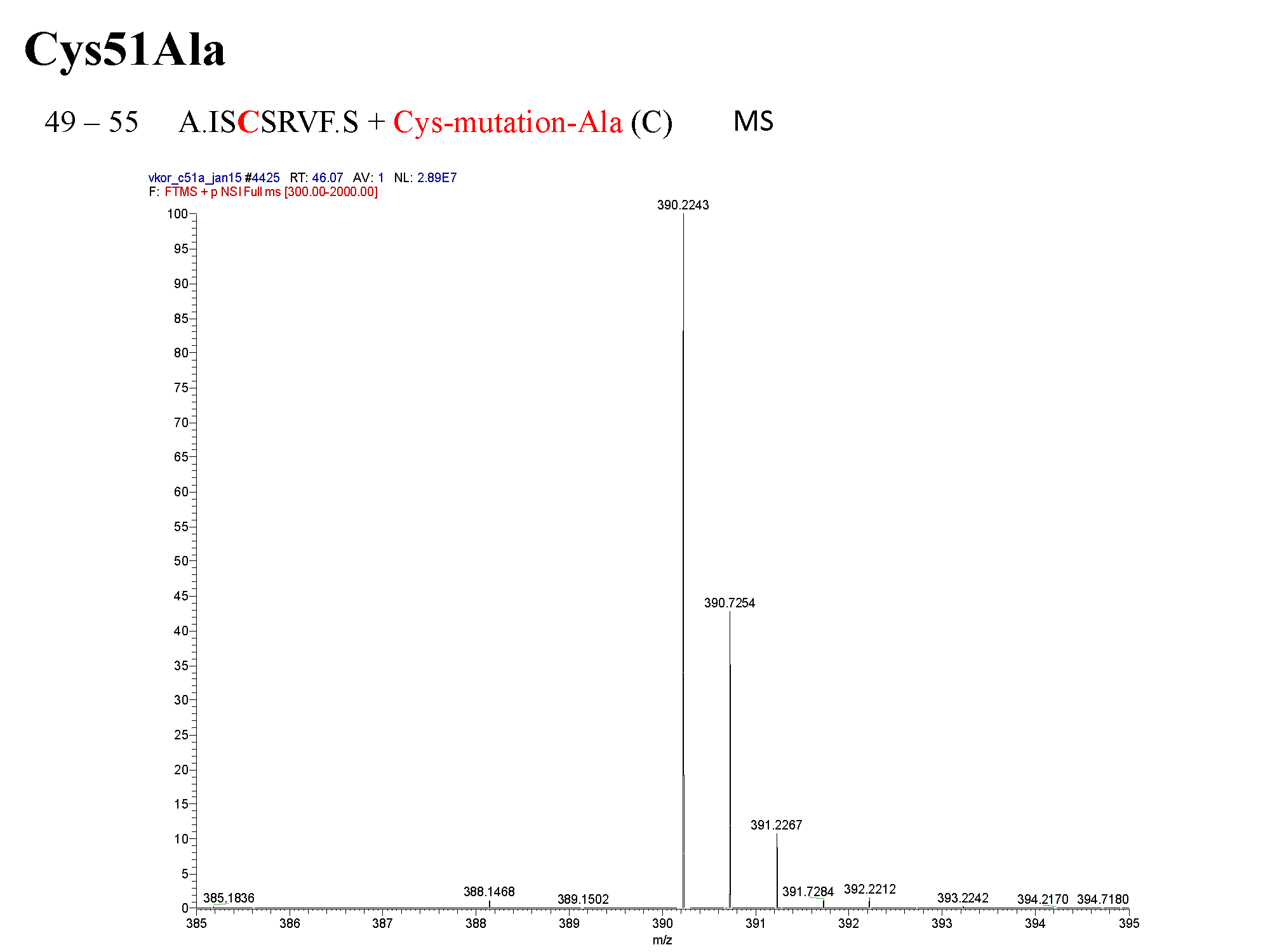

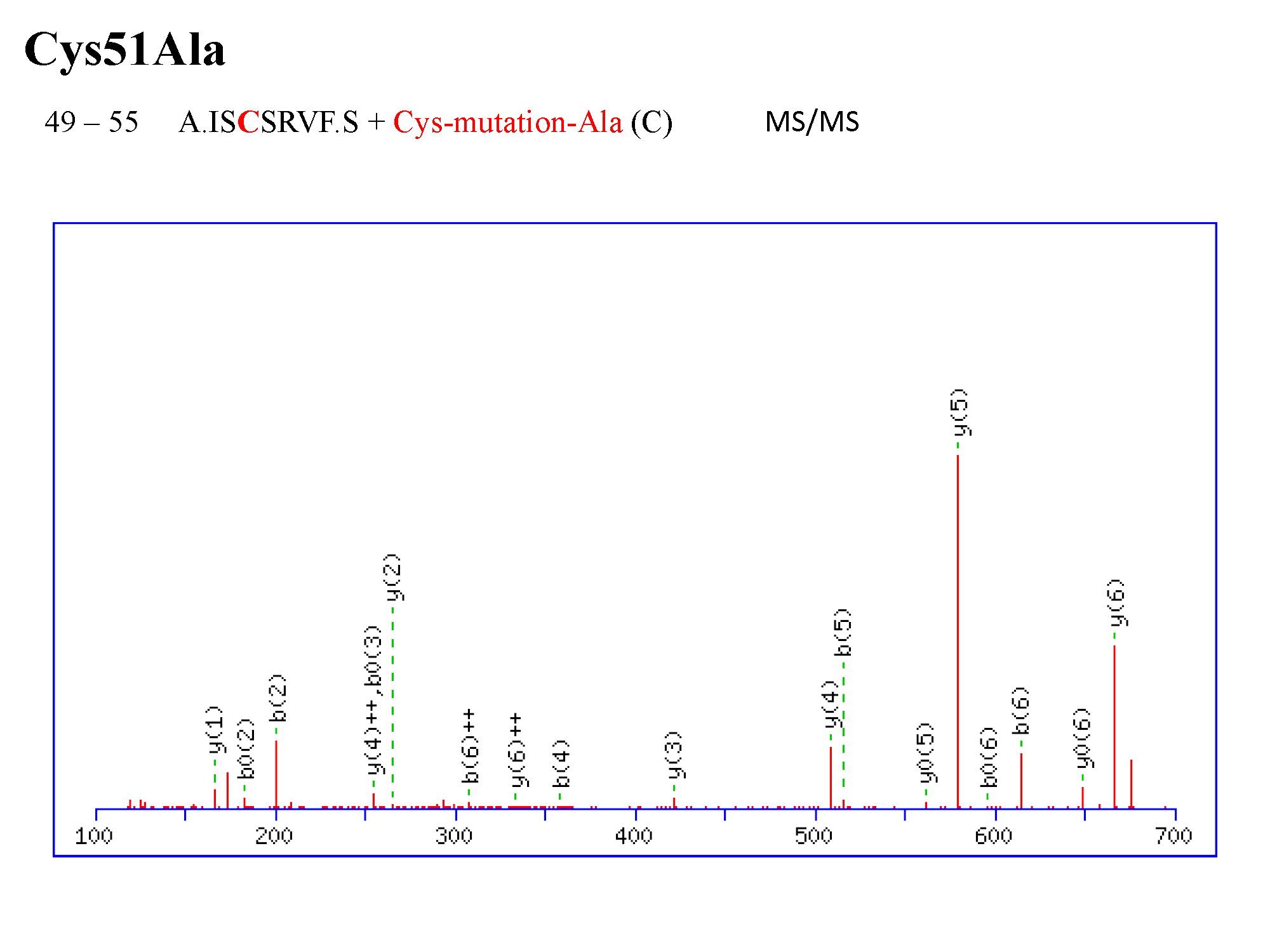

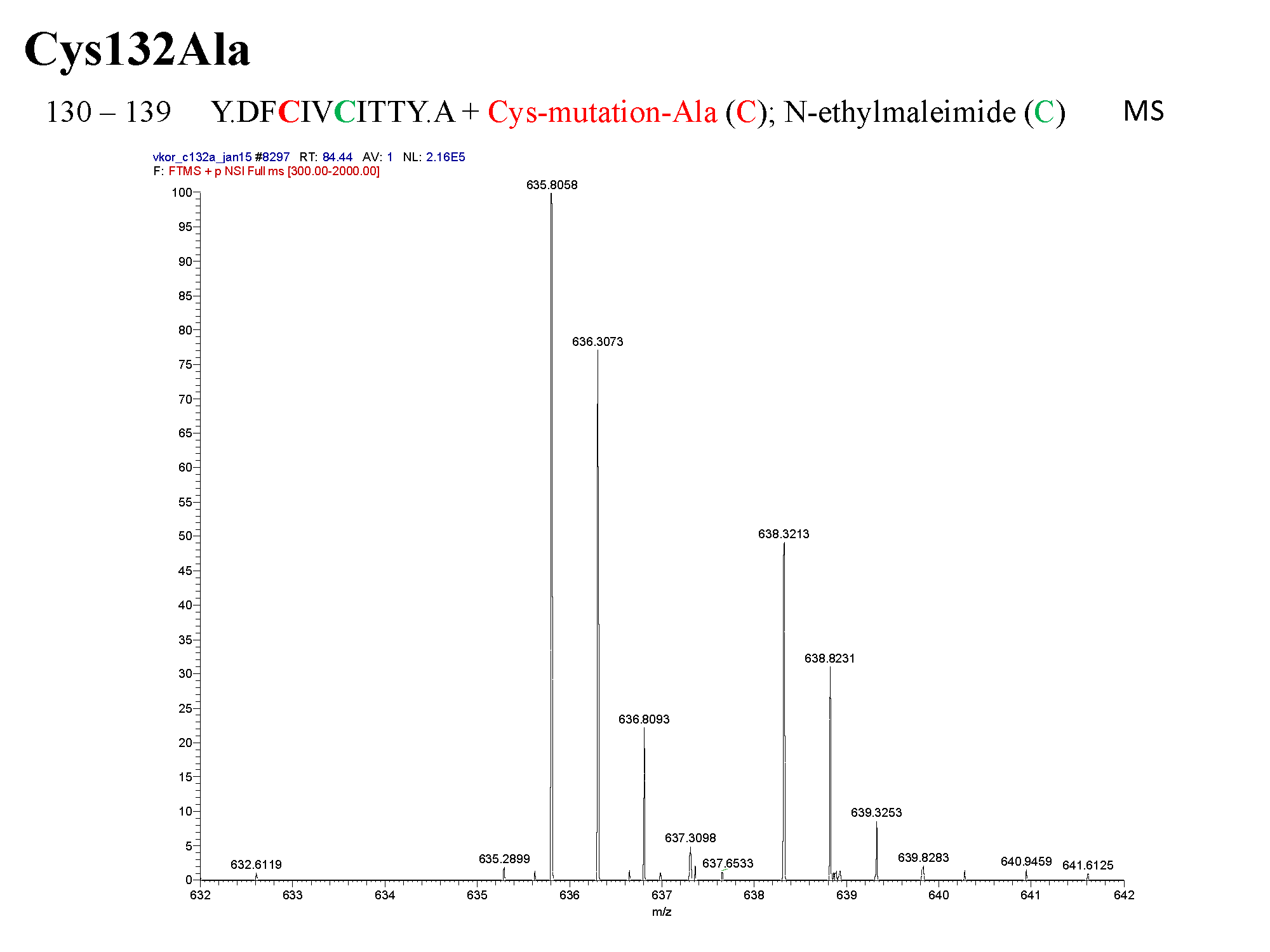

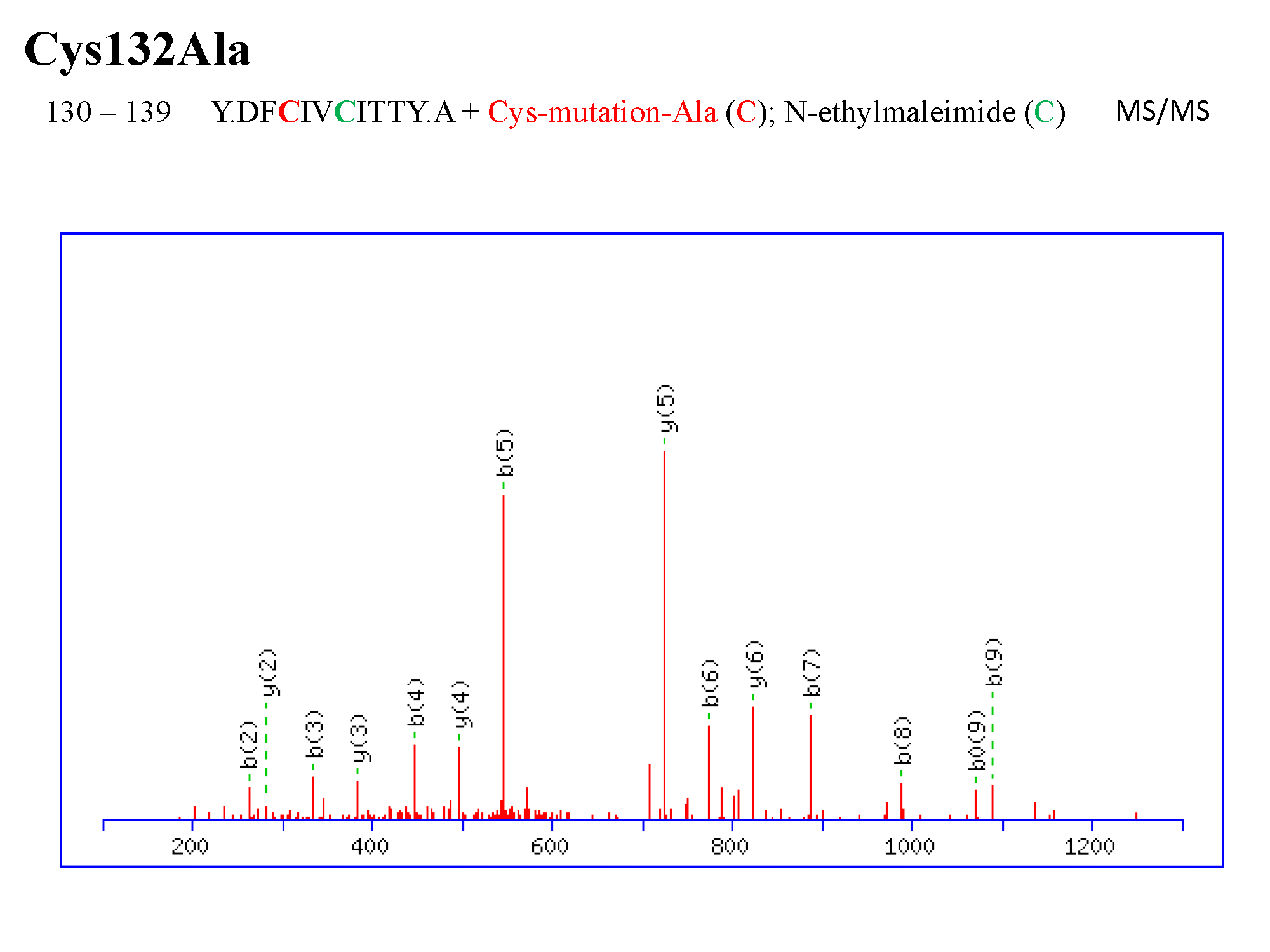

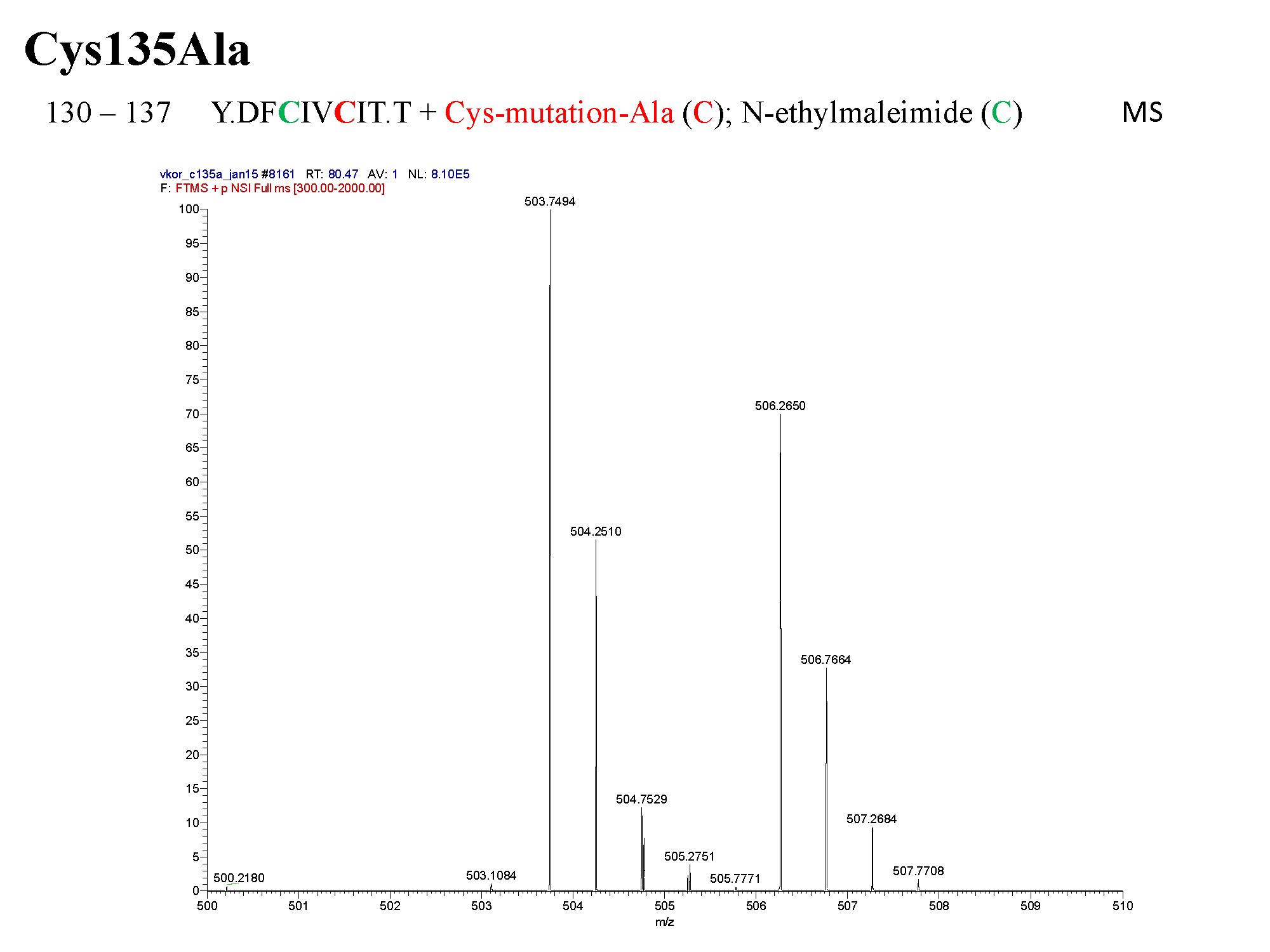

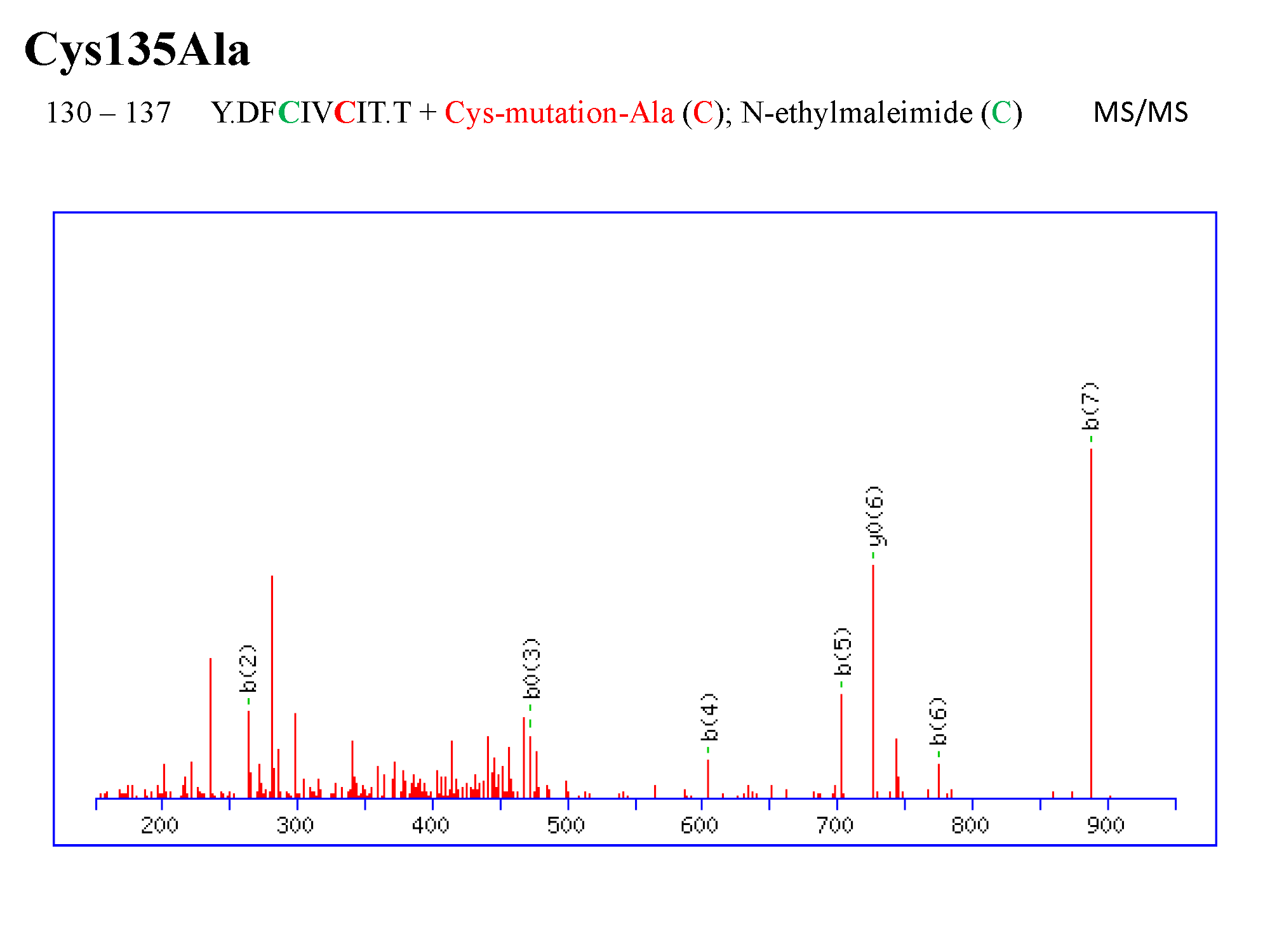

Supplement: Supplementary Figures and Tables [file mmc1.docx]
